# Low-dose statins restore innate immune response in breast cancer cells via suppression of mutant p53

**DOI:** 10.3389/fphar.2025.1492305

**Published:** 2025-05-02

**Authors:** Zi Wang, Meina Shi, Beijia Liu, Xuening Zhang, Wanjun Lin, Yanchao Yang, Zifeng Huang, Dongfan Yang, Tong Chu, Dayuan Zheng, Wenzhe Ma

**Affiliations:** Faculty of Chinese Medicine and State Key Laboratory of Quality Research in Chinese Medicine, Macau University of Science and Technology, Macau, China

**Keywords:** p53, statin, breast cancer, CGAS, STING

## Abstract

**Background:**

Breast cancer’s high recurrence and treatment side effects demand safer, more effective therapies. Mutations in the critical TP53 gene, which normally prevents cancer, can instead promote it. This study explores if low-dose statins can curb mutant p53 activation in breast cancer’s immune signaling, hindering tumor immune evasion.

**Methods:**

The study used diverse breast cancer cell lines with varying p53 statuses. Techniques included Western blot, transfection, qRT-PCR, co-immunoprecipitation, nuclear fractionation, and immunohistochemistry. *In vivo* experiments used BALB/c mice, with bioinformatics analysis via cBioPortal.

**Results:**

The study found that suppressing mutant p53 restores innate immunity and enhances cancer treatment. Low-dose statins promoted IRF3 nuclear translocation by inhibiting mutant p53. Lovastatin treatment *in vivo* increased phosphorylated TBK1 and IRF3 levels and induced CD8^+^ T lymphocyte infiltration in tumors.

**Conclusion:**

The findings suggest low-dose statins can enhance innate immunity in breast cancer by degrading mutant p53, offering new treatment possibilities. Caution is advised, and further research is needed to address limitations and provide solid evidence for clinical use.

## Graphical Abstract

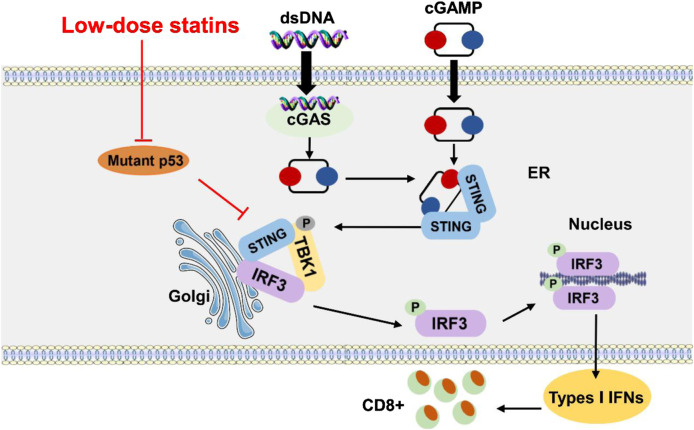

## 1 Introduction

In 2020, breast cancer was diagnosed in 2.3 million women worldwide, with an annual growth rate of approximately 0.6% (Siegel et al., 2024). By 2040, it is projected that there will be over three million new cases and more than one million deaths annually (Arnold et al., 2022). The recurrence rate for breast cancer patients is as high as 10% annually, posing ongoing challenges, especially for those in the first 2 years post-treatment (Colleoni et al., 2016). Breast cancer treatment includes chemotherapy, endocrine therapy, targeted therapy, and immunotherapies. Despite their effectiveness, these treatments often have significant side effects (Ben-Baruch et al., 2015; Groner and Hynes, 2016; Kos et al., 2023; Robson et al., 2023). Therefore, there is a pressing need for safer and more effective breast cancer treatments. However, the development of new drugs involves multiple stages, including theoretical research, laboratory studies, animal trials, clinical research, regulatory approval, and market launch. This process can take several years and cost billions (Singh et al., 2023). Repurposing existing drugs not typically used for breast cancer treatment offers a cost-effective and time-saving strategy for drug development.

Breast cancer is a diverse disease with various biological subtypes, each with unique characteristics and responses to treatment. The TP53 gene is the most commonly mutated gene in breast cancer, found in about 30% of cases (Ungerleider et al., 2018). The protein encoded by the wild-type TP53 gene, p53, plays a vital role in preventing cancer development by interacting with signaling pathways essential for cell division, maintaining genomic stability, apoptosis, autophagy, immune response, and regulating the tumor microenvironment (Hafner et al., 2019). However, mutations in the TP53 gene can compromise this protective function, potentially promoting cancer development. Current research indicates that tumor-associated p53 mutants drive cancer progression, metastasis, and drug resistance. Despite significant progress, no drugs targeting mutant p53 have been clinically approved.

Statins, first used clinically in the late 1980s, have significantly improved the treatment of high cholesterol and ischemic heart disease (Goldstein and Brown, 1990). Recently, researchers have been exploring statins as potential anti-cancer drugs (Wang et al., 2016; Tran et al., 2020). However, some studies do not support the use of statins for cancer treatment or prevention (Seckl et al., 2017). This discrepancy could be due to clinical trial design flaws, such as not selecting patient groups based on vulnerability factors identified in preclinical studies (Abdullah et al., 2018). Another debate concerns the dosage of statins used in cancer management. In the majority of preclinical investigations, statins were administered at concentrations ranging from 2 to 20 μM, a dosage significantly exceeding the levels typically observed in human blood (Tilija Pun and Jeong, 2021). On the other hand, promising results have been obtained using low-dose statins in combination with chemotherapeutic drugs (Janakiram et al., 2013).

Among the strategies targeting mutant p53 for cancer treatment, promoting the degradation of the mutant protein is the most studied and promising (Zawacka-Pankau and Selivanova, 2015). High concentrations of statins have been shown to induce the degradation of p53 mutant proteins and inhibit the growth of cancer cells carrying mutant p53 (Parrales et al., 2016; Ingallina et al., 2018). Furthermore, recent reports suggest that mutant p53, by interfering with the cytoplasmic DNA sensing machinery cGAS-STING-TBK1-IRF3, disrupts both cell-autonomous and non-cell-autonomous signaling, promoting cancer cell survival and evading tumor immune surveillance (Ghosh et al., 2021). Therefore, this study aims to investigate whether low-dose statins can reduce the activation of mutant p53 in breast cancer’s innate immune signaling to inhibit tumor immune evasion.

## 2 Materials and methods

### 2.1 Reagents and antibodies

Fluvastatin, lovastatin, pitavastatin, and simvastatin were obtained from Sigma (St. Louis, MO, United States) and stored at −40°C after dissolution in dimethyl sulfoxide (DMSO) from Acros Organics (Morris Plains, NJ, United States). Puromycin and Tris base were purchased from Sigma Aldrich (St. Louis, MO, United States). Modified Eagle’s medium (MEM), RPMI-1640, and fetal bovine serum (FBS) were obtained from Gibco (NY, United States). Mouse monoclonal p53 antibody (DO-1, SC-126) was sourced from Santa Cruz Biotechnology (Santa Cruz, CA, United States). Antibodies for β-actin, Tubulin alpha, cGAS, TBK1/NAK, Phospho-TBK1/NAK, STING, Phospho-STING, IRF3, Phospho-IRF3, P53, and β-Actin were acquired from Cell Signaling Technology (Danvers, MA, United States). MG132 were purchased from MedChemExpress (Monmouth Junction, NJ, United States). Secondary antibodies anti-rabbit and anti-mouse were obtained from Jackson ImmunoResearch Laboratories (West Grove, PA, United States). The plvx-puro-p53 R280K plasmid was acquired from Huada Gene Co., Ltd. (Shenzhen, China).

### 2.2 Tumor cell culture

Human breast cancer cell lines MDA-MB-231 (p53^R280K^), Sk-Br-3 (p53^R175H^), BT-549 (p53^R249S^), MDA-MB-468 (p53^R273H^), and MCF7 (wild-type p53) and mouse breast cancer cell line 4T1 were procured from ATCC and cultured accordingly in DMEM or RPMI-1640 with 10% FBS and 1% antibiotics. 4T1 cells expressing plvx-puro-p53 R280K were cultured in RPMI-1640 with additional antibiotics, as previously described (Zhang et al., 2020). All cells were maintained at 37°C with 5% CO_2_.

### 2.3 Western blot analysis

Cell samples were collected for Western blot analysis at the end of the expected treatment time. For the cGAS pathway protein expression study, statins were applied to cell lines, and samples were collected for Western blot analysis after the designated treatment periods. The western blot procedure involved protein sample preparation, gel electrophoresis, transfer to a PVDF membrane, blocking, incubation with primary and secondary antibodies, and visualization. Antibodies used included those targeting P53, cGAS, TBK1/NAK, STING, IRF3, β-actin, and Tubulin alpha, each diluted per manufacturer instructions.

### 2.4 Generation of lentiviruses and retroviruses

The shRNA oligonucleotides were designed for knocking down p53 and STING, of which sequences are described as following: shp53-1 sense (CGGCGCACAGAGG AAGAGAAT); shp53-2 sense (GTC​CAG​ATG​AAG​CTC​CCA​GAA); shSTING-1 sense (GCT​GGC​ATG​GTC​ATA​TTA​CAT); shSTING-2 sense (GCAGAGCTATTTC CTTCCACA). Paired oligonucleotides were annealed and inserted into lentiviral expression vectors (pLKO.1). The MISSION non-target shRNA vector, shC002, was used as scrambled controls. Lentivirus production involved transducing 293T cells with the constructs along with psPAX2 and pMD2.G, according to the previously established procedure (Nasri et al., 2014). The culture medium containing lentivirus was added to target cells MDA-MB-231, Sk-Br-3, and 4T1, followed by puromycin selection for 3–6 days. Validation of gene knockdown and overexpression was confirmed through Western blot analysis.

### 2.5 Quantitative RT-PCR analysis

Following cell culture and treatment with fluvastatin or lovastatin, RNA was extracted after 120 h for cDNA synthesis. Quantitative RT-PCR analysis was performed using the ViiA™ 7 Real-Time PCR System, and specific primers (Table 1) were utilized to quantify interferon-stimulated genes (ISGs) expression in the cell lines, in accordance with the published procedure (He et al., 2022).

**TABLE 1:** Primers targeting specific genes.

Target	Primers	Sequences (5′-3′)
Human CXCL10	Forward	GTG​GCA​TTC​AAG​GAG​TAC​CTC
	Reverse	TGA​TGG​CCT​TCG​ATT​CTG​GAT​T
Human ISG15	Forward	AAC​TCA​TCT​TTG​CCA​GTA​CAG​GAG
	Reverse	ATC​TTC​TGG​GTG​ATC​TGC​GCC
mouse IFNB1	Forward	CAG​CTC​CAA​GAA​AGG​ACG​AAC
	Reverse	GGC​AGT​GTA​ACT​CTT​CTG​CAT

### 2.6 Co-immunoprecipitation

Cell lysates were prepared by scraping cells, centrifuging lysates, measuring protein concentration, and storing them at −80°C. Following Cell Lysate Preclearing, Immunoprecipitation was performed by incubating cell lysates with p53 protein and IgG, capturing immune complexes with Protein A/G slurry, washing the beads, and conducting SDS-PAGE electrophoresis for visual analysis. Western blot experiments were carried out using TBK1, P53, and β-actin antibodies on the immunoprecipitated proteins.

### 2.7 Nuclear fractionation

Cells were digested and seeded in culture dishes, treated with fluvastatin, passed, and maintained for up to 120 h. After treatment and incubation, samples were collected by digestion, cell counting, centrifugation, and resuspension in RSB buffer (10 mM NaCl, 1.5 mM CaCl_2_, 10 mM Tris-HCl pH 7.5), as previously described (Udi et al., 2023). The resulting protein samples from cytoplasm and nucleus were subjected to SDS-PAGE and western blot analysis.

### 2.8 Mice and animal care

All animal studies were approved by Macau Food and Animal Inspection Bureau. For the *in vivo* experiments, healthy female BALB/c mice aged 6–8 weeks were prepared for inoculation with 4T1 and 4T1 (P53^R280K^) breast cancer cells. Syngeneic BALB/c mouse models were employed to evaluate tumor growth. On day 0, 4T1 (p53^R280K^) 1 × 10^5^ cells were implanted *in situ* into the mammary fat pad of 6-week-old BALB/c mice. Two days later, the mice were randomly divided into control and treatment groups. The treatment group was given 0.5% CMC-Na lovastatin suspension intragastine 50 mg/kg/2 d, and the control group was given 0.5% CMC-Na intragastine. Starting from the fourth day post-implantation of breast cancer, tumor size was measured using calipers to monitor the growth of *in situ* breast cancer tumors in mice and calculated using the formula volume = (length) (width)^2^/2. Final dosing was on day 22, with tumor sizes measured. On day 23, The mice were euthanized using CO_2_, tumors were excised, volumes recorded, and body weights documented.

### 2.9 Immunohistochemical analysis

Mouse breast cancer tissues in 4% paraformaldehyde underwent immunohistochemical experiments with the following steps: sectioning, dewaxing, hydration, antigen retrieval, endogenous peroxidase removal, blocking, antibody incubation, DAB staining, counterstaining with hematoxylin, dehydration, and cover slipping (Magaki et al., 2019). Slides were observed and photographed under a microscope post-processing.

### 2.10 Bioinformatics

The Cancer Genome Atlas (TCGA) database was used to analyze the different expression of TP53 messenger RNA (mRNA) between normal breast tissue and breast carcinoma tissue, and the clinical data about 7,699 samples were downloaded from the cBioPortal (https://www.cbioportal.org/). The expression level of TP53, co-expression network, and survival were analyzed, as previously described (Donehower et al., 2019). Those with a fold change ≥ 1 and FDR < 0.05 were considered to have statistical significance.

### 2.11 Statistical analysis

Statistical analysis was performed using GraphPad Prism software (v8.0, GraphPad Inc., United States). Sample sizes for *in vivo* experiments were determined based on preliminary data and power analysis conducted with G*Power 3.1.9.7, assuming an effect size of 1.5 (Cohen’s d), α = 0.05, and 80% power, which yielded a minimum requirement of eight mice per group. For *in vitro* experiments, triplicate independent replicates were performed unless otherwise specified, as established in prior studies investigating similar pathways. Data distribution was assessed using Shapiro-Wilk normality tests, and homogeneity of variance was verified via Levene’s test. Parametric tests (Student's t-test for two groups; one-way ANOVA with Tukey’s *post hoc* correction for ≥3 groups) were applied to normally distributed data. Results are presented as mean ± standard deviation (SD) for technical replicates or mean ± standard error of the mean (SEM) for biological replicates, with individual data points plotted where feasible. Multiple comparison adjustments were explicitly predefined, and Tukey’s method was selected to control family-wise error rates while maintaining statistical power. All statistical tests were two-tailed, and significance thresholds were defined as *p < 0.05, **p < 0.01, ***p < 0.001, ****p < 0.0001.

## 3 Results

### 3.1 Mutant p53 inhibits innate immune signaling in breast cancer

**FIGURE 1:**
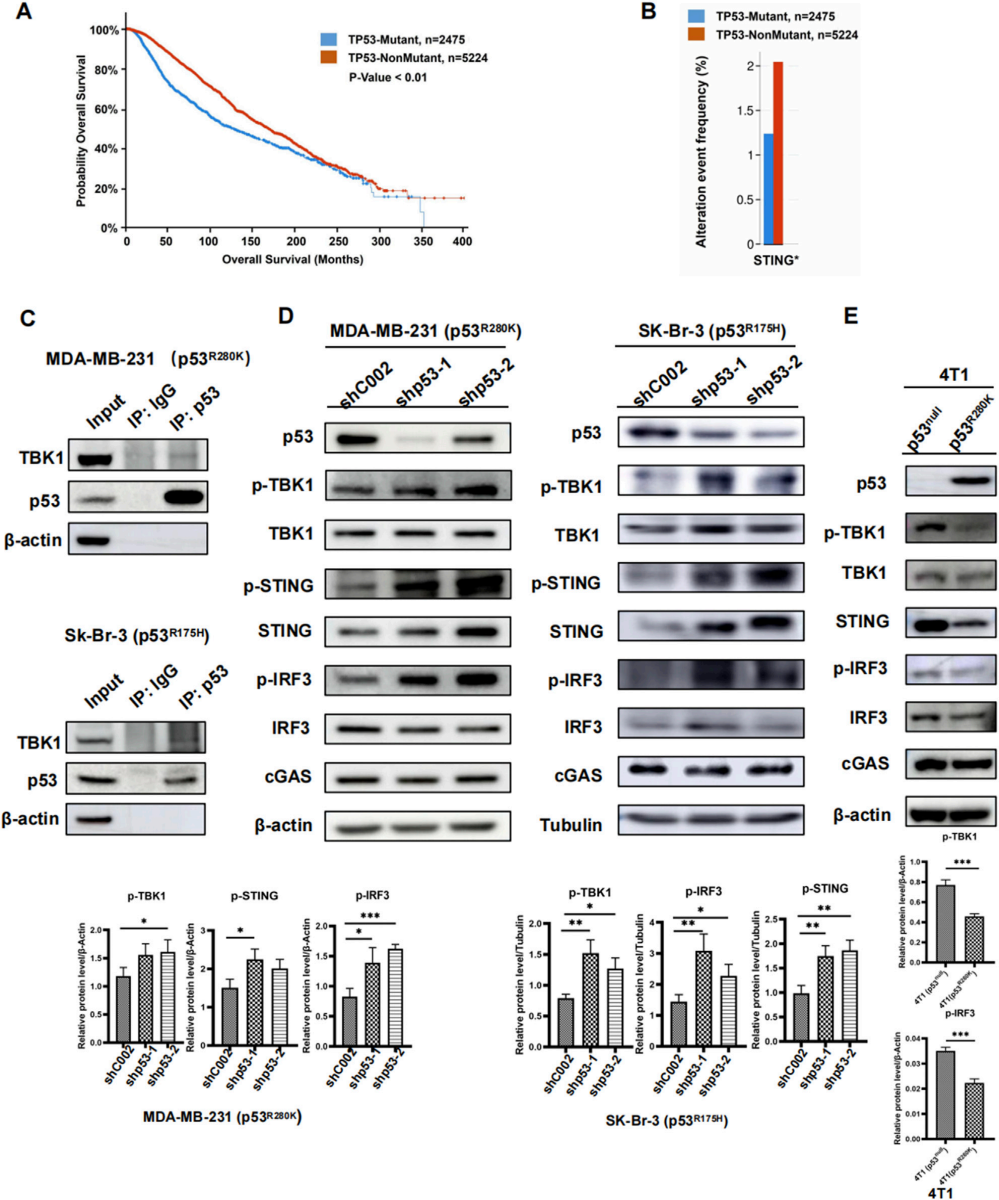
Mutant p53 inhibits innate immune signaling in breast cancer. Survival analysis of TP53-Mutant and TP53-NonMutant groups in breast cancer patients **(A)**. Expression of STING in breast cancer with TP53-Mutant group versus TP53-NonMutant group, *p < 0.05 **(B)**. Immunoprecipitation of mutant P53 in whole-cell lysates from MDA-MB-231 (p53^R280K^) and Sk-Br-3 (p53^R175H^) cells. Immunoblot analysis of lysates and immunoprecipitates **(C)**. Western blot analysis of cGAS-STING pathway proteins in the human breast cancer cell lines MDA-MB-231 (P53^R280K^) and Sk-Br-3 (P53^R175H^) following shp53 **(D)**. Western blot analysis of cGAS-STING pathway proteins in 4T1 cells transfected with p53R280K, using 4T1 (p53^null^) cells transfected with shCOO2 as the control **(E)**.

Approximately 30% of breast cancers harbor p53 mutations (Siegel et al., 2023). To explore the impact of these mutations on patient prognosis and survival rates, we analyzed TP53-Mutant and TP53-NonMutant samples using the cBioPortal System on databases including Breast Cancer (MSK, Cancer Cell 2018), Breast Cancer (MSK, Nature Cancer 2020), Metastatic Breast Cancer (MSK, Cancer Discovery 2022), and Non-CDH1 Invasive Lobular Carcinoma (MSK, 2023). Our findings revealed that the median survival period for the TP53-Mutant group was 126.47 months, compared to 169.23 months for the TP53-NonMutant group, indicating a poorer prognosis associated with p53 mutations in breast cancer patients (Figure 1A).

Furthermore, mutant p53 has been implicated in inhibiting innate immune signaling by disrupting the cGAS-STING-TBK1-IRF3 pathway (Ghosh et al., 2021). Analyzing data from various databases, we observed a significant decrease in STING expression in TP53-Mutant samples compared to TP53-NonMutant samples (Figure 1B). However, no significant differences were found in the expression of cGAS, TBK1, and IRF3 between the two groups (Supplementary Figure S1A). It was reported that the interaction between mutant p53 and TBK1 responses for the comprised innate immune activity (Ghosh et al., 2021). To validate this interaction, we conducted Co-IP experiments in MDA-MB-231 (p53^R280K^) and Sk-Br-3 (p53^R175H^) cells, demonstrating that mutant p53 interacts with TBK1 regardless of mutation sites (Figure 1C).

To investigate its impact on innate immune signaling, we knocked down mutant p53 using short hairpin RNA (shRNA) in human breast cancer cells MDA-MB-231 and Sk-Br-3. Consistent with our bioinformatic analysis, the knockdown of mutant p53 led to a significant increase in cellular STING levels. Notably, there were no significant alterations in TBK1, IRF3, and cGAS protein expression (Figure 1D). Additionally, the levels of phosphorylated TBK1 (p-TBK1), the active form of the protein, and its substrates (p-STING and p-IRF3) significantly increased after mutant p53 knockdown (Figure 1D). To further investigate the impact of mutant p53 on innate immunity, we generated cell lines in mouse breast cancer 4T1 cells with a p53 null background. These cell lines stably expressed either R280K mutant p53 (p53^R280K)^ or an empty vector (p53^null^). Compared to the p53^null^ cells, p53^R280K^ cells exhibited significant reduction in p-TBK1, p-STING, p-IRF3, and STING, while overall protein levels of TBK1, IRF3, and cGAS remained unchanged (Figure 1E). Taken together, these results suggest that suppressing mutant p53 may restore the cell’s innate immune response and contribute to more effective cancer treatment.

### 3.2 Prolonged low-dose statins reduce mutant p53 and activate innate immunity in breast cancer cells

**FIGURE 2:**
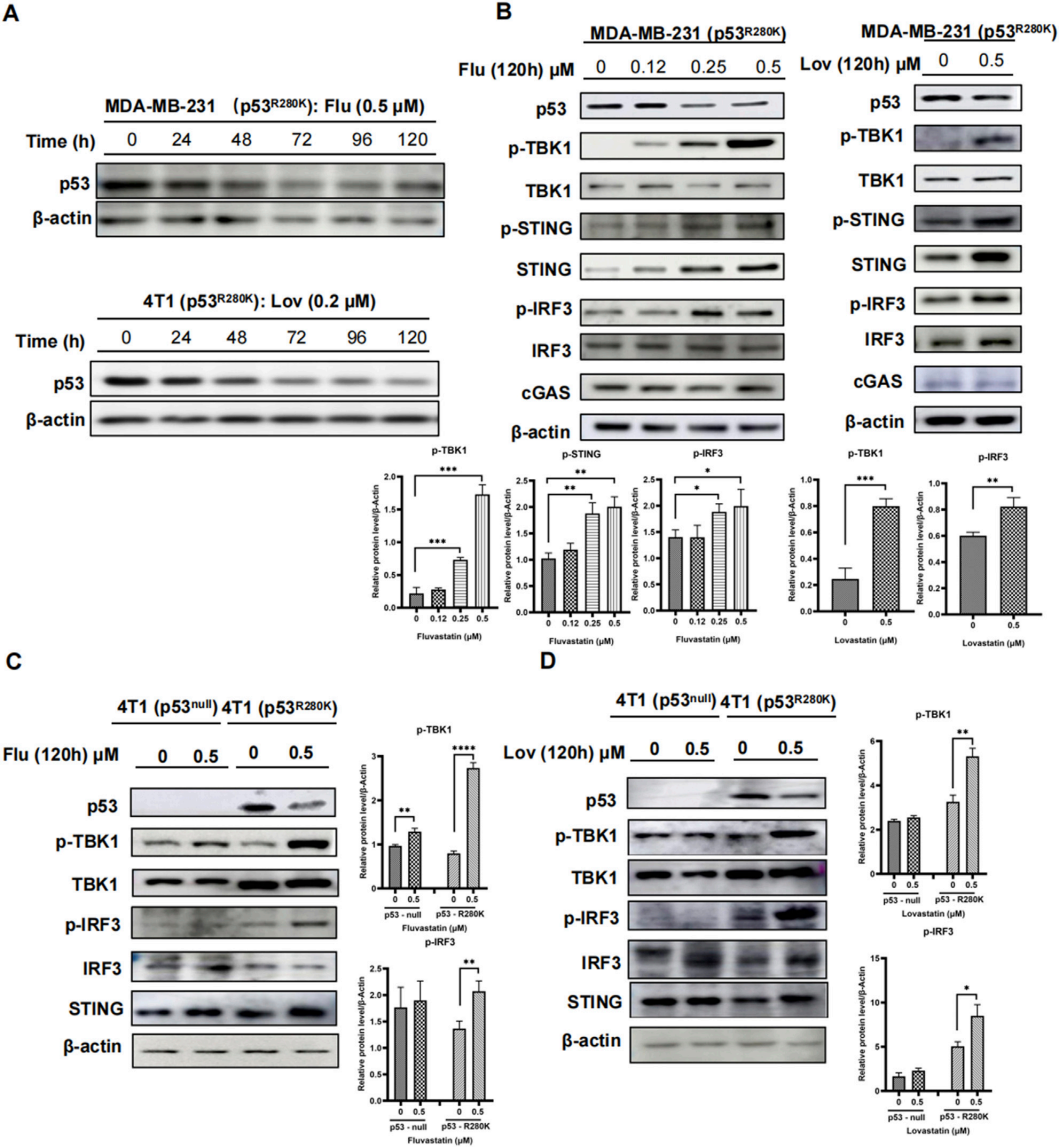
Prolonged low-dose statins reduce mutant p53 and activate innate immunity in breast cancer cells. Western blot analysis of MDA-MB-231 (p53^R280K^) and 4T1 (p53^R280K^) cells treated with low-dose statins collected at 0, 24, 48, 72, 96, and 120 h **(A)**. MDA-MB-231 (p53^R280K^) cells were treated with indicated concentrations of fluvastatin (0, 0.12, 0.25, 0.5 μM) and lovastatin (0, 4 μM) for 120 h, respectively, and cell samples were collected for western blot experiments **(B)**. Empty vector plasmid shC002 transfected 4T1 (p53^null^) cells and mutant p53-transfected 4T1 (p53^R280K^) cells were treated with indicated concentrations of fluvastatin (0, 0.5 μM) and lovastatin (0, 0.5 μM) for 120 h, respectively, and cell samples were collected for western blot experiments **(C, D)**.

Statins, commonly used to lower cholesterol, have recently emerged as potential anticancer agents due to their ability to degrade mutant p53 proteins (Parrales et al., 2016; Ingallina et al., 2018). We first confirmed this selective impact of statins on mutant p53 proteins in Sk-Br-3 and MDA-MB-231 cells (Supplementary Figures S1B, C), while wild type p53 in MCF7 cells remained unaffected (Supplementary Figure S1D). Notably, similar to most *in vitro* studies, statins were administrated at the micromolar range (2–20 μM) in these experiments for a period of up to 48 h, which exceeds the possible concentration of statins in human serum (Tilija Pun and Jeong, 2021). Therefore, we examined the effect of low-dose statins on mutant p53 proteins. As illustrated in Figure 2A, the application of 0.5 μM fluvastatin resulted in a decline in mutant p53 protein after 48 h in MDA-MB-231 cells. This reduction stabilized at 72 h and did not exhibit further significant decreases up to 120 h. Similar effects were observed with 0.2 μM lovastatin in 4T1 cells stably transfected with mutant p53 R280K (Figure 2A).

Subsequently, we explored the activation status of the cGAS-STING pathway in MDA-MB-231 cells following the notable decrease in mutant p53 induced by statins. As expected, high-dose statins decreased mutant p53 levels and concomitantly increased phosphorylated TBK1, STING, and IRF3 levels in MDA-MB-231 cells. The MG132 proteasome inhibitor can counteract the effects of high-concentration lovastatin on p53 protein regulation in MDA-MB-231 cells. Treatment with the proteasome inhibitor MG132 rescues p53 from the suppressive effects of high-dose lovastatin in MDA-MB-231 cells (Supplementary Figure S2A). In contrast, these effects were absent in wild type p53 MCF7 cells (Supplementary Figure S2B). Intriguingly, low-dose fluvastatin treatment led to a modest decrease in mutant p53 over an extended period (up to 120 h), accompanied by marked dose-dependent increase in phosphorylated TBK1, STING, and IRF3 (Figure 2B). Similar effects were observed with prolonged lovastatin treatment at 0.5 μM (Figure 2B). This was recapitulated in 4T1 (p53^R280K^) cells treated either by fluvastatin or lovastatin, while not in 4T1 (p53^null^) cells (Figures 2C, D). These results indicate that extended low-dose statin treatment can activate innate immunity in breast cancer cells harboring mutant p53.

### 3.3 Low-dose statins boost IRF3 nuclear translocation and interferon-stimulated genes expression in mutant p53 breast cancer cells

**FIGURE 3:**
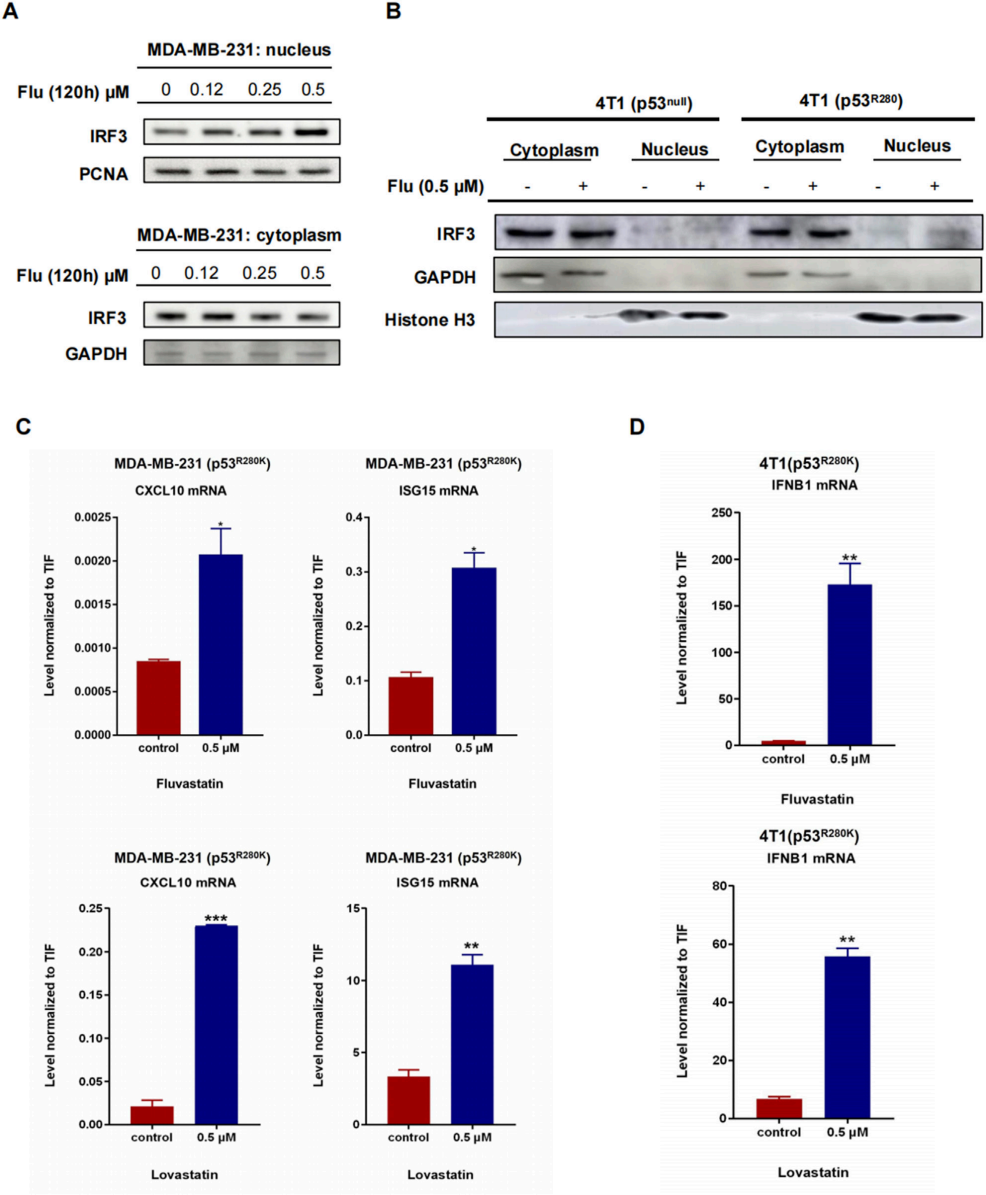
Low-dose statins boost IRF3 nuclear translocation and ISGs in mutant p53 breast cancer. MDA-MB-231 (p53R280K) cells were treated with indicated concentrations of fluvastatin (0, 0.12, 0.25, 0.5 μM) for 120 h, followed by nuclear-cytoplasmic fractionation experiments and western blot analysis to assess IRF3 protein expression **(A)**. 4T1 (p53^null^) cells transfected with empty vector plasmid shC002 and mutant p53-transfected 4T1 (p53^R280K^) cells were treated with indicated concentrations of fluvastatin (0, 0.5 μM) for 120 h, then subjected to nuclear-cytoplasmic fractionation experiments and western blot analysis to evaluate IRF3 protein expression **(B)**. MDA-MB-231 (p53^R280K^) cells were treated with 0.5 μM fluvastatin and 0.5 μM lovastatin for 120 h, respectively, followed by cell sample collection for RT-PCR detection of CXCL10 and ISG15 mRNA. N = 3, *p < 0.05, **p < 0.01, ***p < 0.001 **(C)**. Mutant p53-transfected 4T1 (p53^R280K^) cells were treated with 0.5 μM fluvastatin and 0.5 μM lovastatin for 120 h, respectively, and cell samples were collected for RT-PCR analysis of IFNB1 mRNA. N = 3, **p < 0.01 **(D)**.

In resting cells, the transcription factor IRF3 predominantly resides in the cytoplasm. However, activation of the STING/TBK1/IRF3 pathway triggers its migration to the nucleus, where it regulates the expression of type I interferons (Zhang et al., 2021). To investigate whether low-dose statins facilitate IRF3 nuclear translocation, we conducted nuclear-cytoplasmic fractionation experiments in MDA-MB-231 cells. Treatment with low-dose fluvastatin for 120 h resulted in a dose-dependent increase in nucleus IRF3 protein, accompanied by a corresponding decline in the cytoplasm (Figure 3A). Similarly, 4T1 (p53^R280K^) cells exhibited the same trend, while 4T1 (p53^null^) cells did not (Figure 3B). These findings suggest that low-dose statins effectively promote IRF3 nuclear translocation by inhibiting mutant p53 in breast cancer cells. To validate this effect, we performed RT-PCR analysis of the interferon-stimulated genes, including CXCL10, ISG15 and IFNB1, in MDA-MB-231 and 4T1 (p53^R280K^) cells treated with 0.5 μM statins for 120 h. Remarkably, all these interferon-stimulated genes exhibited a robust increase following statin treatment (Figures 3C, D).

### 3.4 STING knockdown reduces low-dose statin-induced innate immunity activation

**FIGURE 4:**
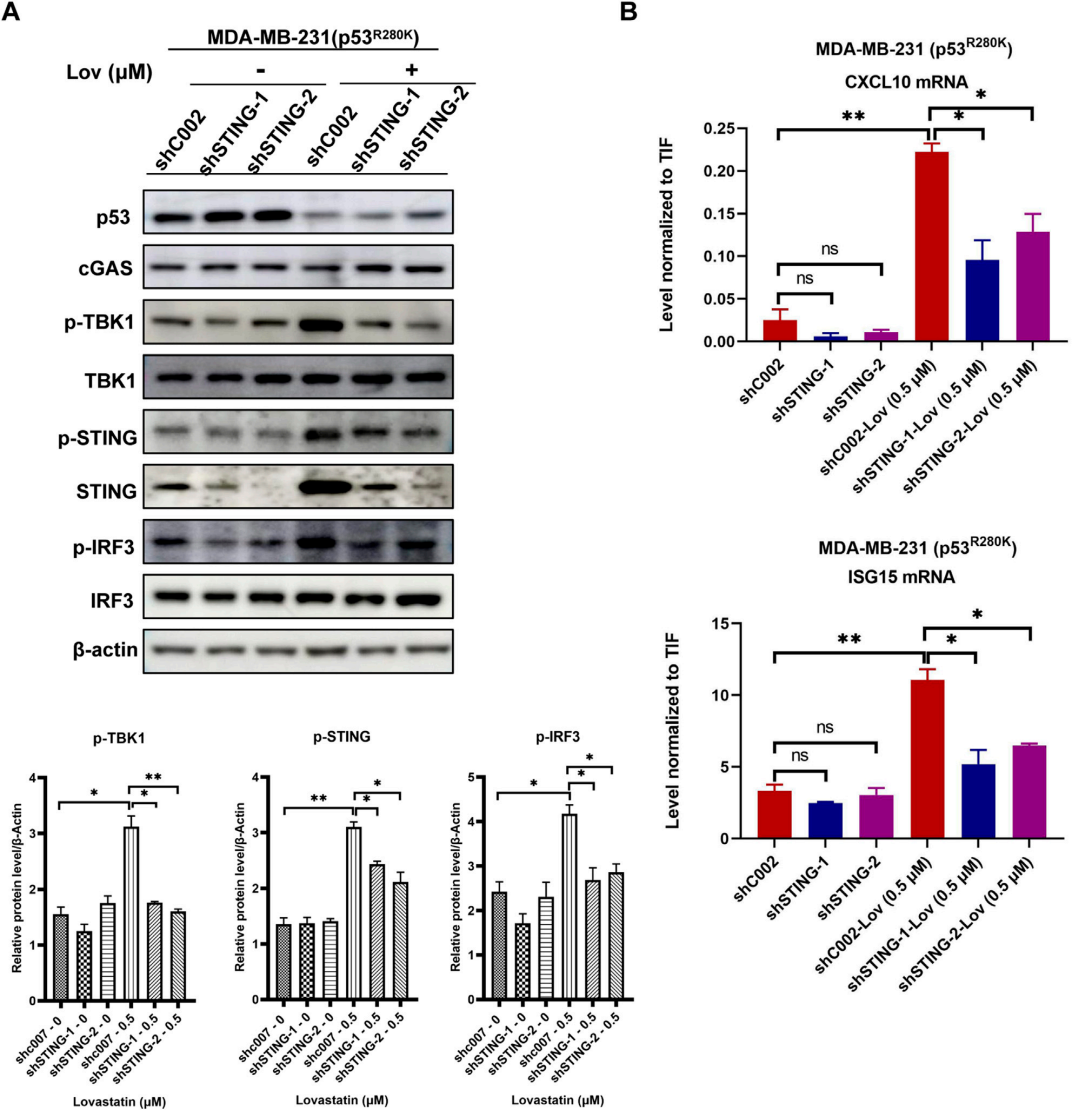
STING gene knockdown reduces low-dose statin-induced innate immunity activation. MDA-MB-231 cells transfected with shC002, shSTING-1, and shSTING-2 were treated with 0.5 μM lovastatin for 120 h followed by western blot experiments **(A)**. MDA-MB-231 cells transfected with shC002, shSTING-1, and shSTING-2 were treated with 0.5 μM lovastatin for 120 h followed by RT-PCR experiments. N = 3, *p < 0.05, **p < 0.01 **(B)**.

To investigate the role of the STING pathway in regulating innate immunity and verify its specificity in mediating the effects of low-dose statins, we employed lentivirus-mediated RNA interference to silence the STING genes in MDA-MB-231 cells. Specifically, both sequences targeting STING significantly reduced STING protein levels compared to the control (shC002) (Figure 4A). Upon 120 h treatment with 0.5 μM lovastatin, the control cells exhibited increased phosphorylation levels of TBK1, STING, and IRF3. Notably, silencing the STING gene effectively attenuated the enhanced phosphorylation levels of these proteins induced by low-dose lovastatin (Figure 4A). Subsequently, we evaluated the expression of interferon-stimulated genes in STING knockdown MDA-MB-231 cells after treatment with 0.5 μM fluvastatin or lovastatin for 120 h. Compared to the shC002 cells, the knockdown of STING genes partially reversed the upregulation of CXCL10 and ISG15 gene expression induced by low-dose fluvastatin and lovastatin (Figure 4B).

### 3.5 Statins elicit innate immune responses against breast cancer growth *in vivo*

**FIGURE 5:**
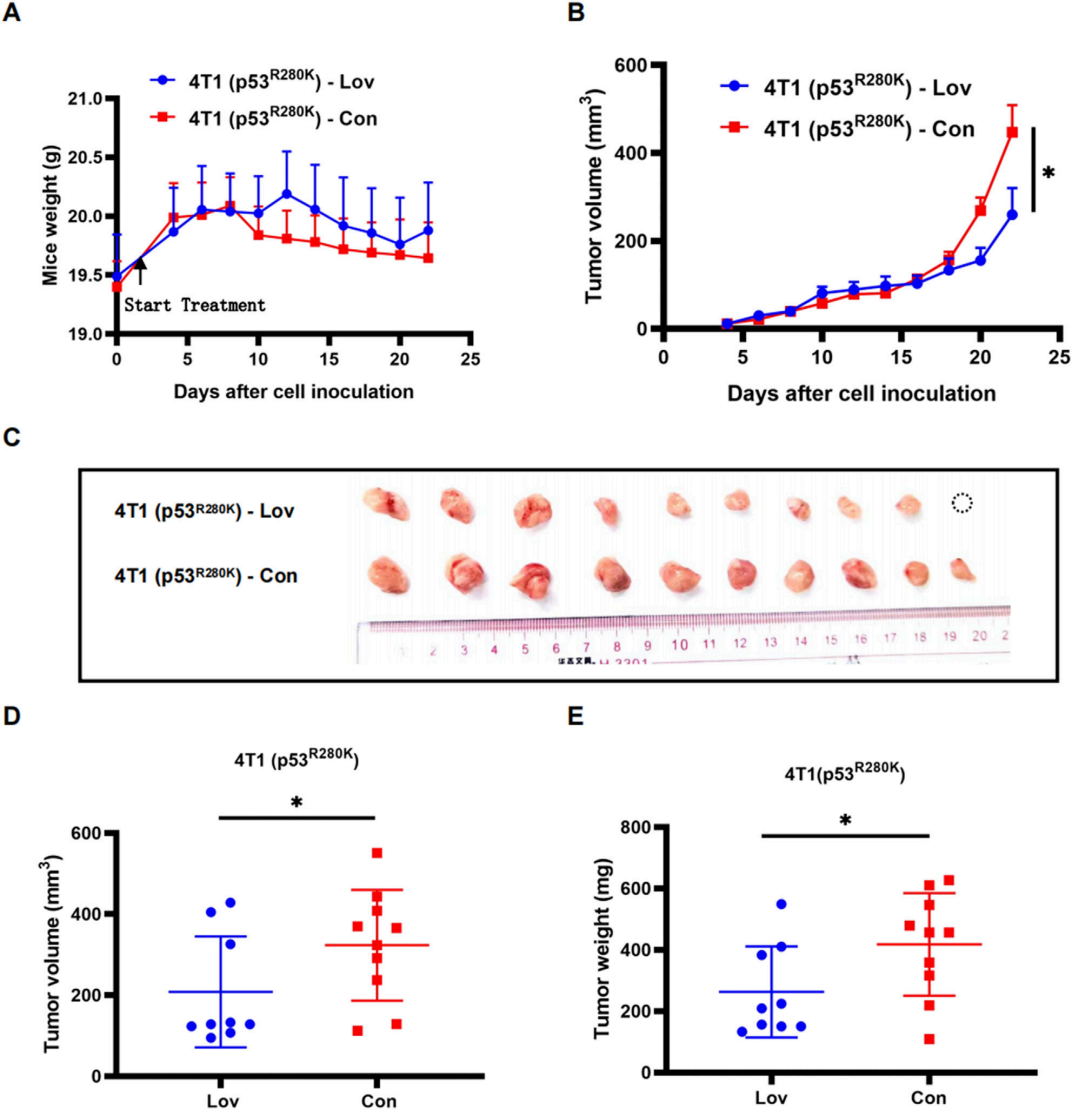
Statin inhibition of proliferation in mutant p53 breast tumors in an intact host immune system. Monitoring of body weight during a 3-week treatment period of 4T1 (p53^R280K^) lovastatin and control groups. 4T1 (p53^R280K^) treated group n = 9, 4T1 (p53^R280K^) control group n = 10. *p < 0.05 **(A)**. Monitoring of mammary tumor growth in the two groups mentioned in **(A)** over the 3-week treatment period. *p < 0.05 **(B)**. Photographic documentation of tumors from dissected BALB/c mice in the two groups mentioned in **(A)** following treatment termination **(C)**. Statistical analysis of tumor volumes from **(C)**, *p < 0.05 **(D)**. Statistical analysis of tumor weights from **(C)**, *p < 0.05 **(E)**.

Within the tumor microenvironment, the cGAS-STING pathway serves as a communication channel between tumor cells and immune cells, functioning in a non-cellular autonomous manner (Liu et al., 2015). To investigate the effect of statins on innate immunity and anti-tumor activity *in vivo*, we established an orthotopic allograft model using 4T1 (p53^R280K^) cells. Initially, there was no obvious difference in tumor volume between the lovastatin treatment group and the control group until 2 weeks after drug administration (Figure 5B). However, by day 22, the tumor volume in the lovastatin-treated group was significantly smaller than that in the control group (Figures 5B–E). Interestingly, starting from the second week, the average weight of the control group was lower than that of the treatment group (Figure 5A). This discrepancy could be attributed to active tumor proliferation in the control group, necessitating a substantial nutrient supply, while potential metabolic changes might have led to reduced appetite in mice, resulting in weight loss.

**FIGURE 6:**
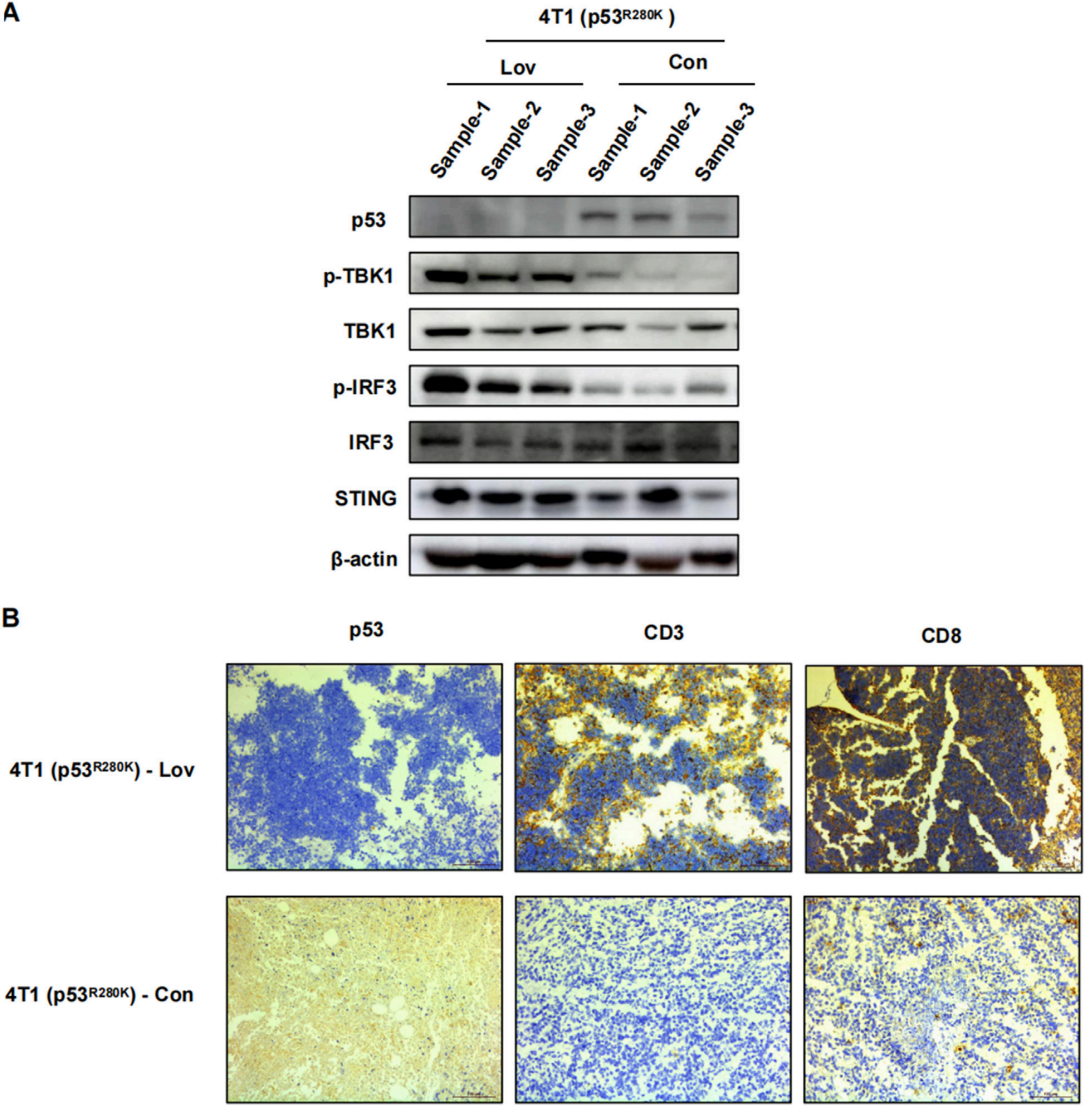
Statin activation of innate immune suppression in mutant p53 breast cancer tumor growth. 4T1 (p53^R280K^) BALB/c mice were euthanized on day 23, tumors were excised, sectioned, and subjected to immunoblot analysis of tumor tissue **(A)**. Representative immunohistochemistry images of p53, CD3^+^CD8^+^ T lymphocyte infiltration. Magnification is 200* **(B)**.

To establish a mechanistic link between the *in vivo* and *in vitro* findings, we performed Western blot analysis on 4T1 (p53^R280K^) tumor lysates to evaluate innate immunity proteins. Lovastatin treatment significantly increased the levels of phosphorylated TBK1 and IRF3 (Figure 6A). Furthermore, in line with enhanced innate immunity promoting the recruitment of T lymphocytes into tumors (Zhang et al., 2021), we observed abundant CD8^+^ T lymphocyte infiltration in lovastatin-treated 4T1 (p53^R280K^) tumors (Figure 6B).

## 4 Discussion

Statins, commonly used to lower cholesterol, are increasingly recognized for their potential anticancer properties (Walker et al., 2015; Beckwitt et al., 2018; Nowakowska et al., 2021). High-dose statins can degrade mutant p53 protein, potentially suppressing innate immunity activation (Parrales et al., 2016; Ghosh et al., 2021). This finding led us to investigate the relationship between statins and breast cancer immunity. We used various cell lines and methodologies to confirm statins’ role in reducing mutant p53 levels and activating innate immunity. Our study highlights a mechanism where low-dose statins may activate the cGAS-STING pathway with antitumor activity by selectively lowering mutant p53 protein levels. This activation may occur through various pathways, including modulating immune-related gene expression and influencing immune cell infiltration within the TME. This expands statin applications and provides a new perspective on their potential role in cancer therapy.

Recent findings suggest that p53 may significantly impact tumor immunity beyond its canonical anti-tumor activities. Wild-type p53 enhances NK cell-mediated cytotoxicity by upregulating ULBP1 and ULBP2 expression (Textor et al., 2011). It can also reduce the suppression of T cells by tumor cells and strengthen the anti-tumor immune response by inhibiting PD-L1 expression (Cha et al., 2016; Biton et al., 2018). In contrast, mutant p53 may lose the tumor-suppressive functions of wild-type p53 and acquire new activities that promote tumor growth and immune evasion. For instance, mutant p53 can enhance NF-κB signaling, leading to increased nuclear localization of p65 and upregulated expression of pro-inflammatory mediators (Weisz et al., 2007; Cooks et al., 2013). Mutant p53 can also influence macrophage behavior by secreting miR-1246 within exosomes, thereby supporting tumor progression (Cooks et al., 2018). Within the TME, the loss of wild-type p53 promotes the recruitment of tumor-supportive myeloid cells, while the presence of mutant p53 leads to the development of highly suppressive Treg populations (Bezzi et al., 2018; Togashi et al., 2019; Blagih et al., 2020). Therefore, p53-targeting immunotherapies could offer new cancer treatment strategies. Our study confirms that statins, particularly at low doses, can promote mutant p53 protein degradation, thereby activating the cGAS-STING pathway to enhance immune responses and facilitate immune infiltration. This research provides crucial insights for developing more effective immunotherapeutic strategies and introduces new perspectives for personalized tumor treatments.

Despite promising findings from various studies, the impact of statin on cancer managements remains a topic of debate within the scientific community (Walker et al., 2015; Beckwitt et al., 2018; Nowakowska et al., 2021). A large-scale simulated randomized trial involving 17, 372 cancer patients diagnosed with colorectal, breast, prostate, and bladder cancers did not demonstrate a significant influence of statins on either cancer-specific survival or overall survival outcomes (Emilsson et al., 2018). A study by Peltomaa et al. focusing on a cohort of prostate cancer patients found that statins did not reduce the risk of prostate cancer recurrence or prostate cancer-related mortality in individuals not undergoing androgen deprivation therapy (Peltomaa et al., 2021). These findings underscore the need for a more nuanced understanding of how statins interact with specific types of cancer. Our research delves deeper into the potential anti-tumor effects of statins, shedding light on statins’ ability to modulate mutant p53 protein levels and activate the natural immune response in breast cancer cells. Conversely, when examining MCF7 cells containing wild type p53, statin treatment did not significantly alter p53 protein expression levels nor activate the natural immune response. This distinction underscores the importance of understanding the molecular characteristics of tumors and their response to statin therapy, suggesting the possibility of personalized treatment strategies based on the genetic profile of the tumor, such as targeting patients with p53 mutations for statin therapy.

Experimental models typically employ statin concentrations in the range of 2–20 μM (Parrales et al., 2016; Ingallina et al., 2018; Huang et al., 2021). Clinical pharmacokinetic analyses demonstrate that circulating statin levels in patients generally maintain concentrations between 10–200 ng/mL (equivalent to 25–500 nM), representing an order-of-magnitude reduction compared to *in vitro* experimental conditions (Mysore et al., 2021; Anderson et al., 2022). To bridge this translational gap, we implemented a physiologically relevant dosing regimen approximating 0.5 μM (202 ng/mL lovastatin and 207 ng/mL fluvastatin, calculated based on molecular weights of 404.5 g/mol and 414.5 g/mol respectively). While supraphysiological statin concentrations (>1 μM) demonstrate potent pro-apoptotic effects in neoplastic cells through pleiotropic mechanisms (Huang et al., 2021; 2024), such pharmacological effects exceed clinically attainable plasma levels by 5–10 fold. This concentration-dependent disparity underscores the importance of distinguishing between *in vitro* cytotoxicity and *in vivo* therapeutic efficacy. Our experimental design therefore utilizes statin concentrations within the clinically observable range, thereby offering enhanced translational value for evaluating statins' therapeutic potential in oncology while controlling for hypertherapeutic artifact. This approach emphasizes the regulation of the innate anti-tumor immunity rather than the direct induction of cell death.

In addition to the orthotopic allograft 4T1 (p53^R280K^) model, we tested lovastatin’s effect on a p53 mutant mice model with germline R172H mutation, a homolog of the human TP53 R175H. However, lovastatin did not significantly alter spontaneous tumorigenesis or extend the median survival time of approximately 24–26 weeks (data not shown). This discrepancy between the effect of statins on established tumors and spontaneous tumorigenesis underscores the complexity of mutant p53 proteins in tumor development and progression. It may also suggest that mutant p53 proteins in different types of cells within the tumor microenvironment may respond differently to statin treatment, contributing uniquely to anti-tumor immunity. Notably, we observed that lovastatin treatment significantly alleviated cachexia symptoms induced by the spontaneous tumors in p53^R172H/R172H^ mice, including hepatosplenomegaly, weight loss, and ascites, and effectively reduced the propensity for tumor metastasis (data not shown). These findings suggest that while statins may not be suitable as a sole anticancer agent, they hold potential for combination therapy and for improving cachexia in patients with advanced tumors.

Our research highlights the potential of low-dose statins to enhance the immune response in p53 mutant breast cancer. However, it’s important to recognize certain limitations of our study. Primarily, our findings are based on *in vitro* cell experiments. Therefore, they require further validation through animal models or clinical trials to confirm the effectiveness and safety of low-dose statins in real-world settings. Another constraint is our exclusive focus on the response of p53 mutant breast cancer cells. We did not consider other types of cancer cells or cells with different immune statuses. As a result, our conclusions may not be applicable to other types of cancer or cells with varied immune conditions. Future research should expand the scope to assess the effects of low-dose statins across different cancer types and immune statuses. In conclusion, while our study suggests that low-dose statins could enhance the immune response in p53 mutant breast cancer, these findings should be interpreted with caution. Further comprehensive research is indispensable to address current limitations and furnish robust evidence for the clinical utilization of statins in cancer therapy.

## 5 Conclusion

Our study examined the role of statins in decreasing mutant p53 protein levels in breast cancer and in activating the innate immune response. We specifically investigated the potential of low-dose statins to stimulate the innate immune response in p53 mutant breast cancer. Our findings suggest that low-dose statins may have the ability to enhance the restoration of innate immune responses. These findings may help to elucidate the role of statins in tumorigenesis and innate immune response, and can provide a reference for the realization of more precise and personalized immunotherapy for the use of statins in breast cancer treatment in the future.

## Data Availability

The original contributions presented in the study are included in the article/Supplementary Material, further inquiries can be directed to the corresponding author.
