# Supplementary material for: Low-dose statins restore innate immune response in breast cancer cells via suppression of mutant p53
Source: Front Pharmacol. 2025 May 2;16:1492305. doi: 10.3389/fphar.2025.1492305 (PMC12081456; doi:10.3389/fphar.2025.1492305)
Supplement: Supplementary file 1 [file Table1.docx]

***Supplementary information***


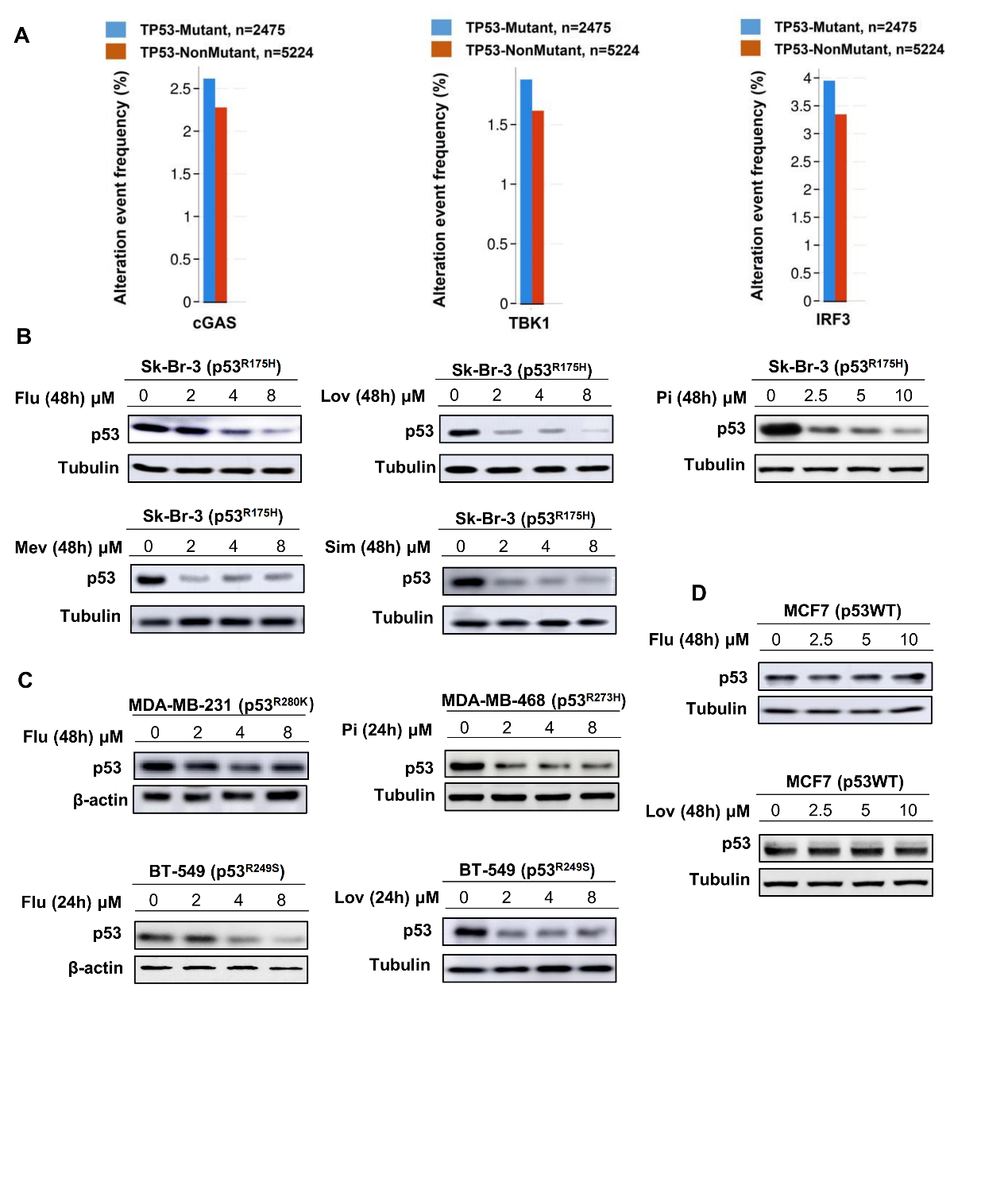


**Figure S1.** High-Dose statins reduce mutant p53 in breast cancer cells.

Expression of cGAS, TBK1 and IRF3 in breast cancer with TP53-Mutant group versus TP53-NonMutant group (A). SK-BR-3 (P53^R175H^) cells were treated with specified concentrations of fluvastatin (0, 2, 4, 8 μM), lovastatin (0, 2, 4, 8 μM), pitavastatin (0, 2.5, 5, 10 μM), mevastatin (0, 2, 4, 8 μM), and simvastatin (0, 2, 4, 8 μM) for 48 h, followed by collection of cell samples for western blot analysis (B). MDA-MB-231 (P53^R280K^) cells were treated with specified concentrations of fluvastatin (0, 2, 4, 8 μM) for 48 hours, MDA-MB-468 (P53^R273H^) cells with pivastatin (0, 2, 4, 8 μM) for 24 h, BT-549 (P53^R249S^) cells with fluvastatin (0, 2, 4, 8 μM) and lovastatin (0, 2, 4, 8 μM) for 24 h, followed by collection of cell samples for western blot analysis (C). MCF7 (P53^WT^) cells were treated with specified concentrations of fluvastatin (0, 2.5, 5, 10 μM) and lovastatin (0, 2.5, 5, 10 μM) for 48 h, followed by collection of cell samples for western blot analysis (D). All data are from at least three independent experiments.


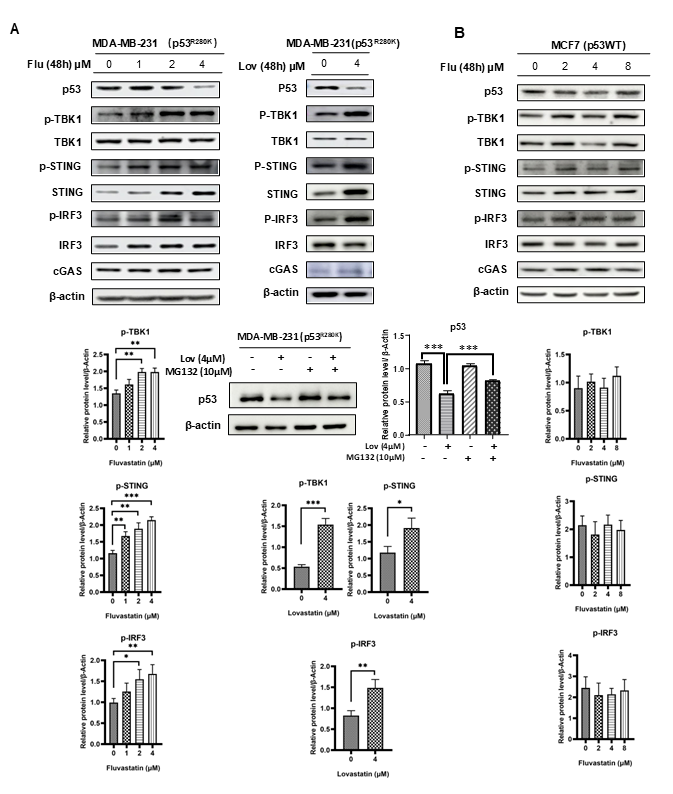


**Figure S2.** High-Dose statins suppress mutant p53 mediated activation of innate immunity in human breast cancer.

MDA-MB-231 (p53^R280K^) cells were treated with specified concentrations of fluvastatin (0, 1, 2, 4 μM) or lovastatin (0, 4 μM) for 48 h, followed by the collection of cell samples for western blot analysis. Cells were pretreated for 48 h with DMSO (control) or lovastatin (4 μM), and MG132 (10 μM) was added during the final 9 h to block proteasome function. Representative western blots with the indicated antibodies are shown (A). MCF7 (p53^WT^) cells were treated with specified concentrations of fluvastatin (0, 2, 4, 8 μM) for 48 h, followed by the collection of cell samples for western blot analysis (B). All data are from at least three independent experiments.
