# Supplementary material for: Low-dose statins restore innate immune response in breast cancer cells via suppression of mutant p53
Source: Front Pharmacol. 2025 May 2;16:1492305. doi: 10.3389/fphar.2025.1492305 (PMC12081456; doi:10.3389/fphar.2025.1492305)
Supplement: Supplementary file 2 [file Presentation1.pptx]

## Slide 1
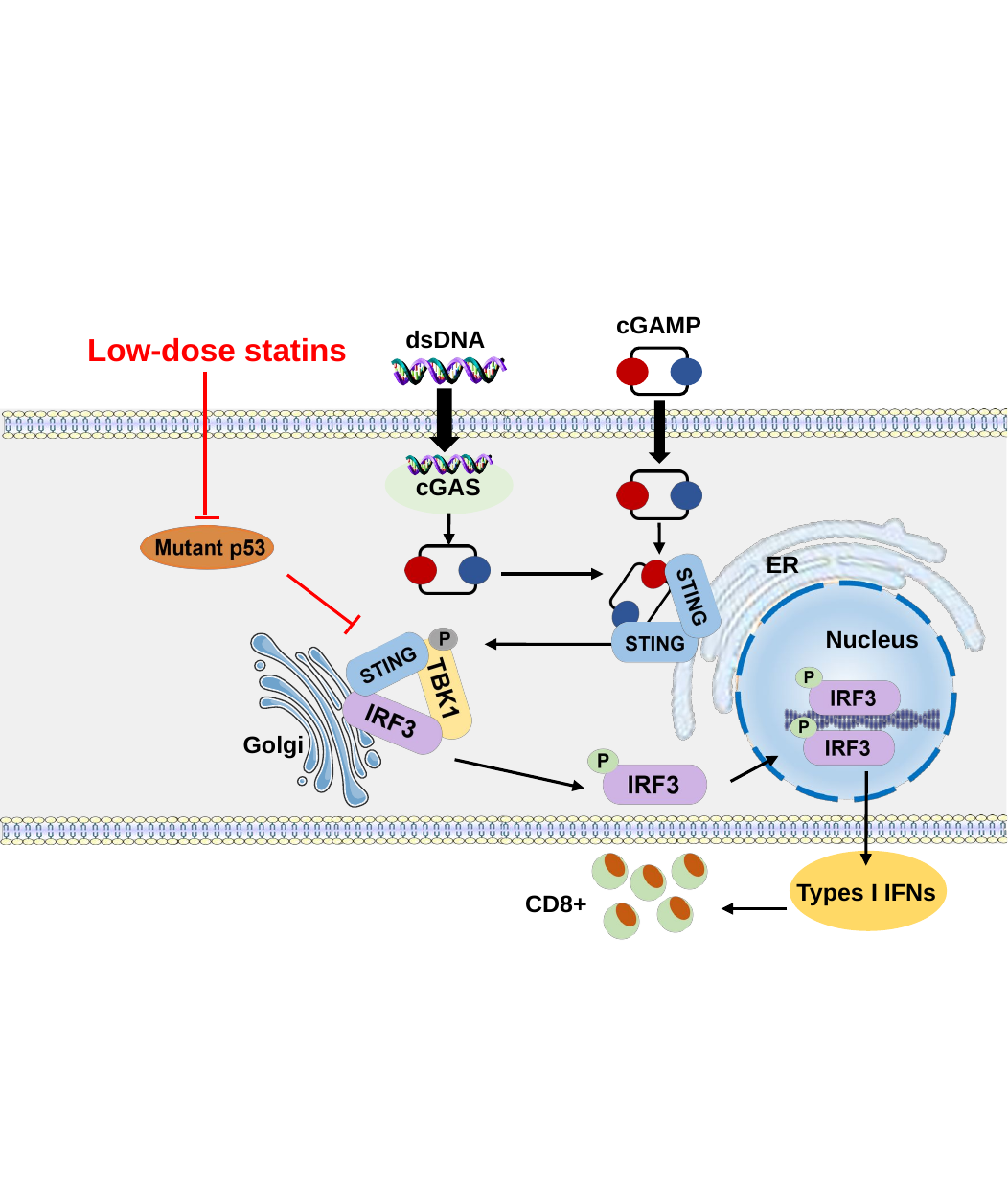

cGAMP
dsDNA
Low-dose statins
cGAS
ER
Nucleus
Golgi
Types I IFNs
CD8+

## Slide 2
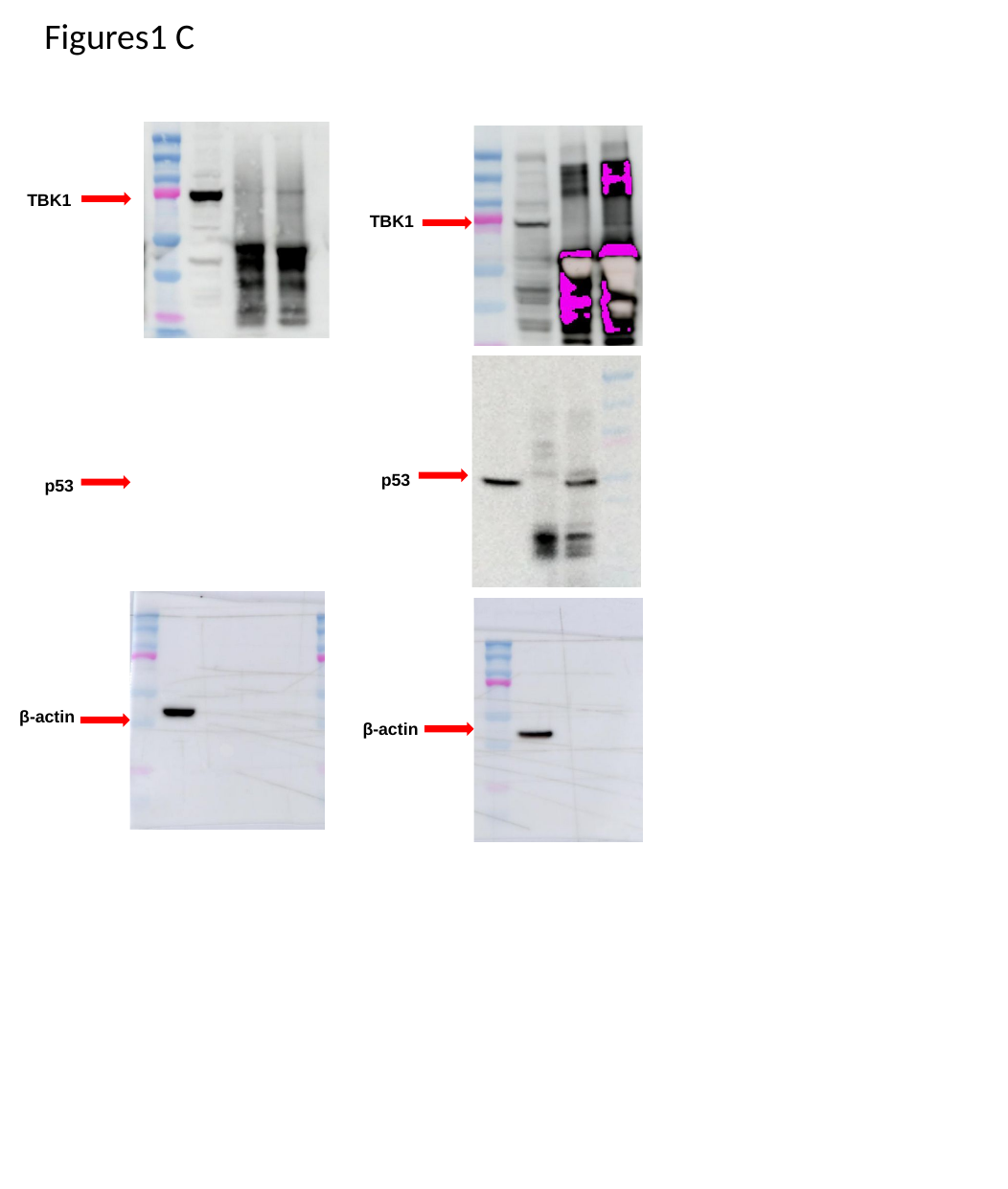

Figures1 C
TBK1
TBK1
p53
p53
 β-actin
 β-actin

## Slide 3
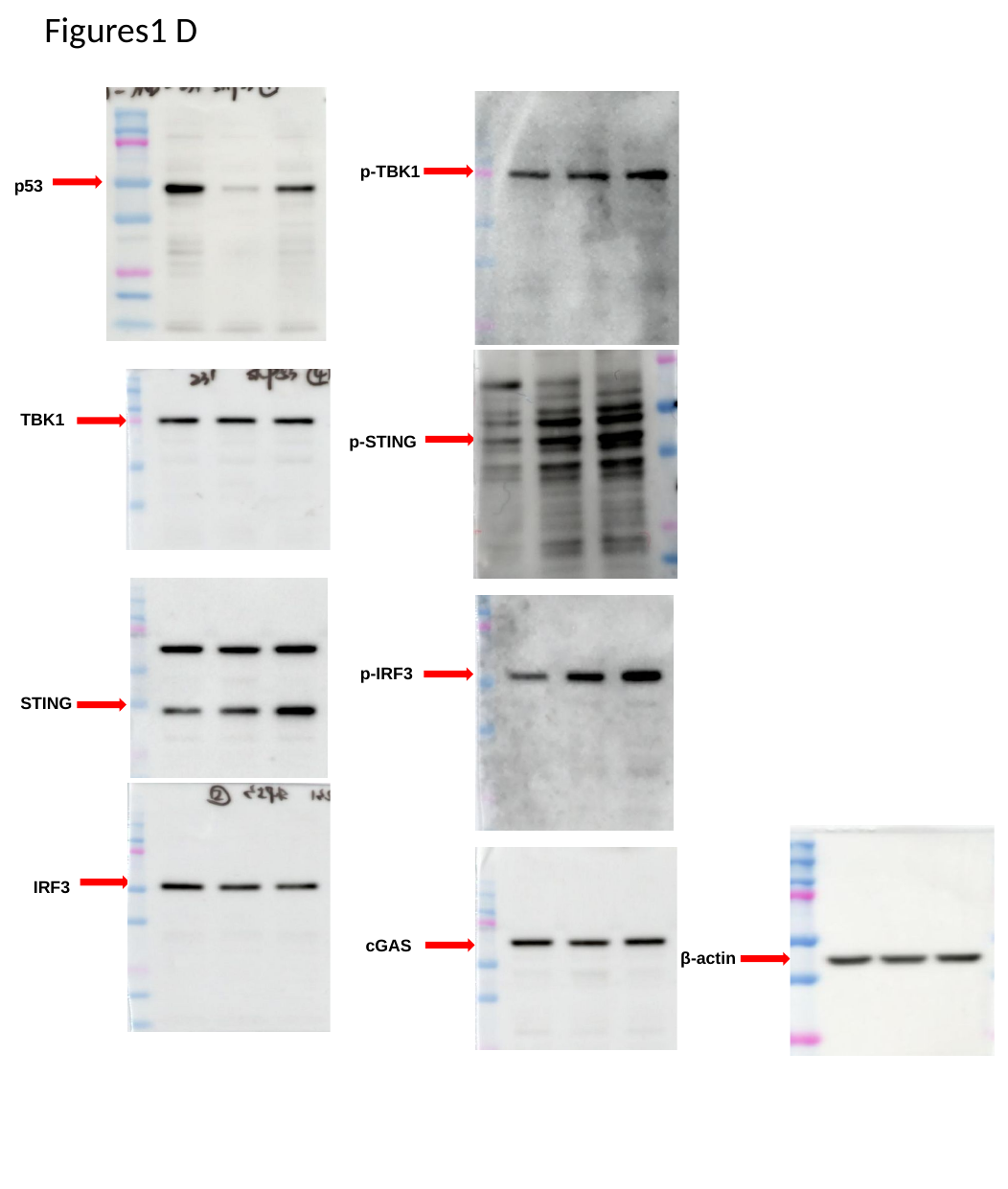

Figures1 D
 p53
p-TBK1
TBK1
p-STING
p-IRF3
STING
 IRF3
 cGAS
 β-actin

## Slide 4
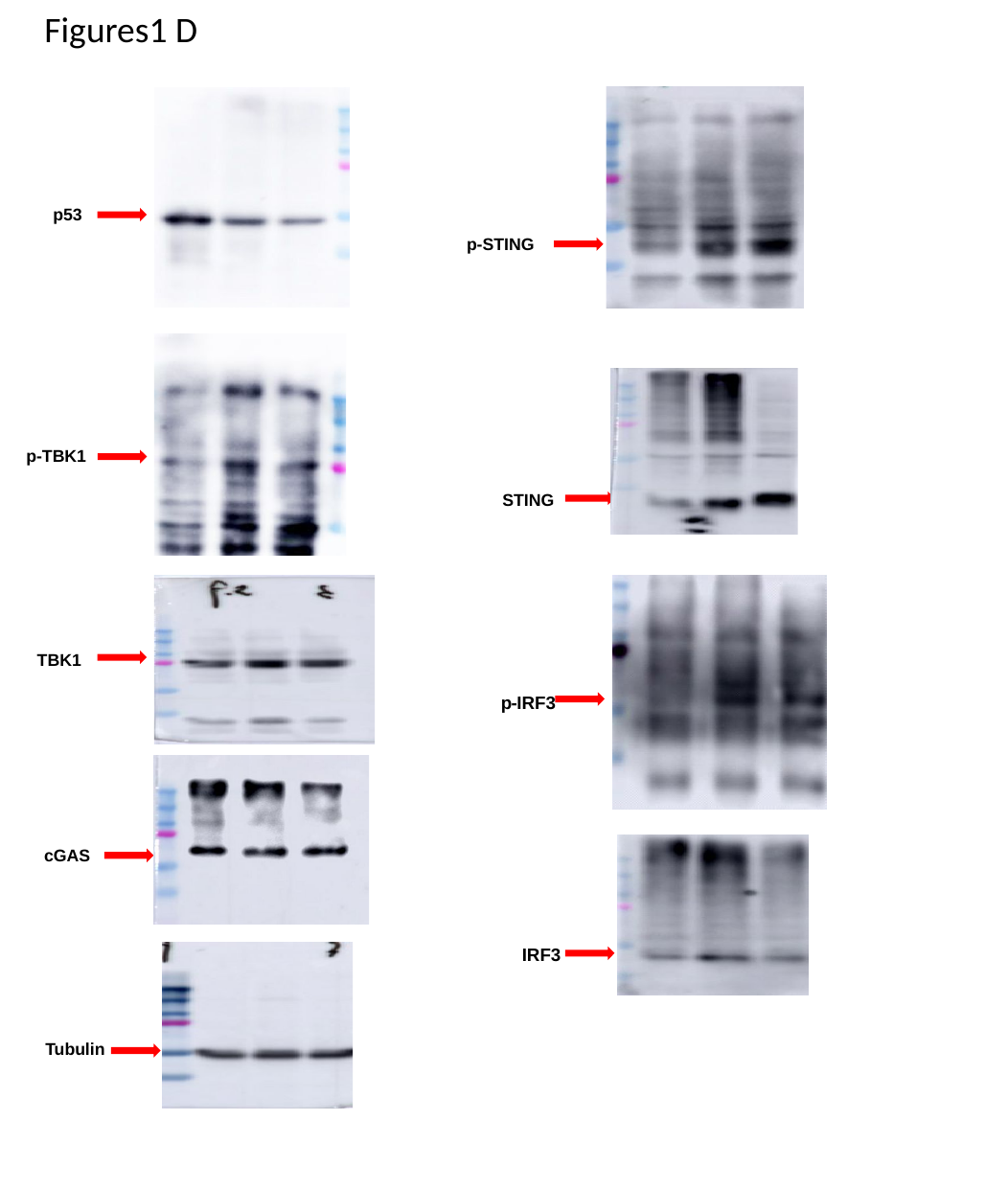

Figures1 D
 p53
p-STING
p-TBK1
STING
TBK1
p
-
IRF3
 cGAS
IRF3
 Tubulin

## Slide 5
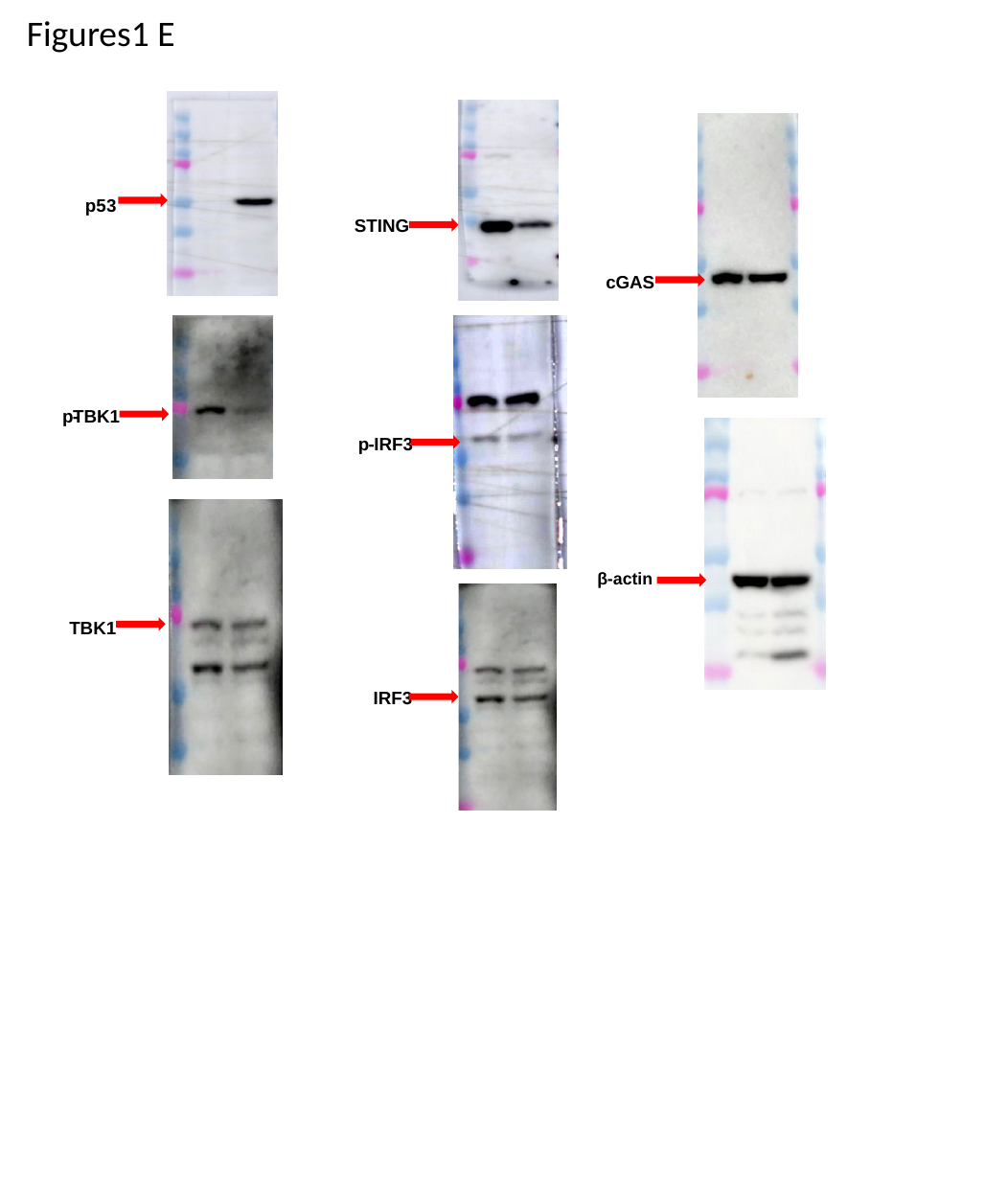

Figures1 E
p53
STING
cGAS
p
-
TBK1
p
-
IRF3
 β-actin
TBK1
IRF3

## Slide 6
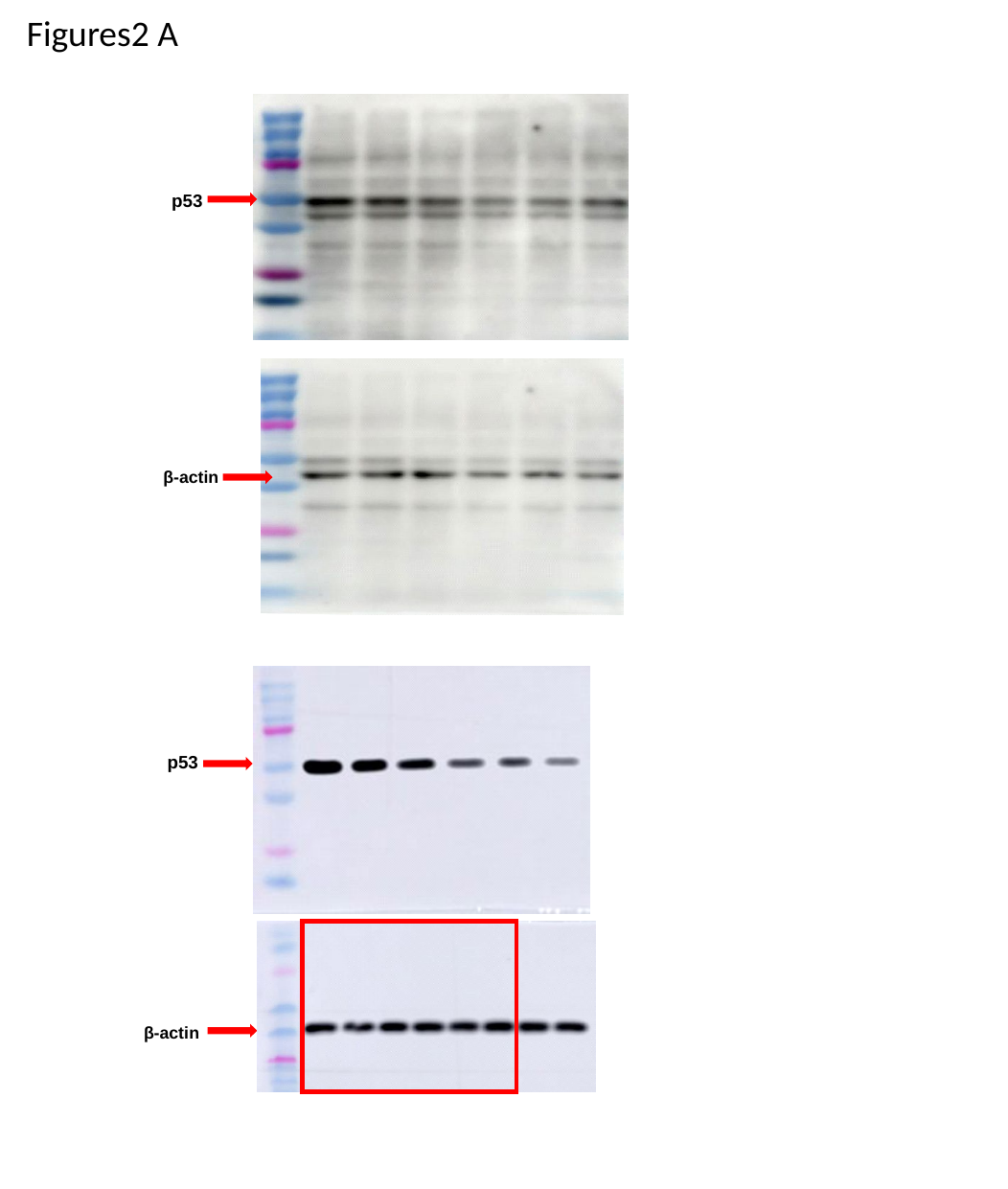

Figures2 A
p53
β-actin
p53
β-actin

## Slide 7
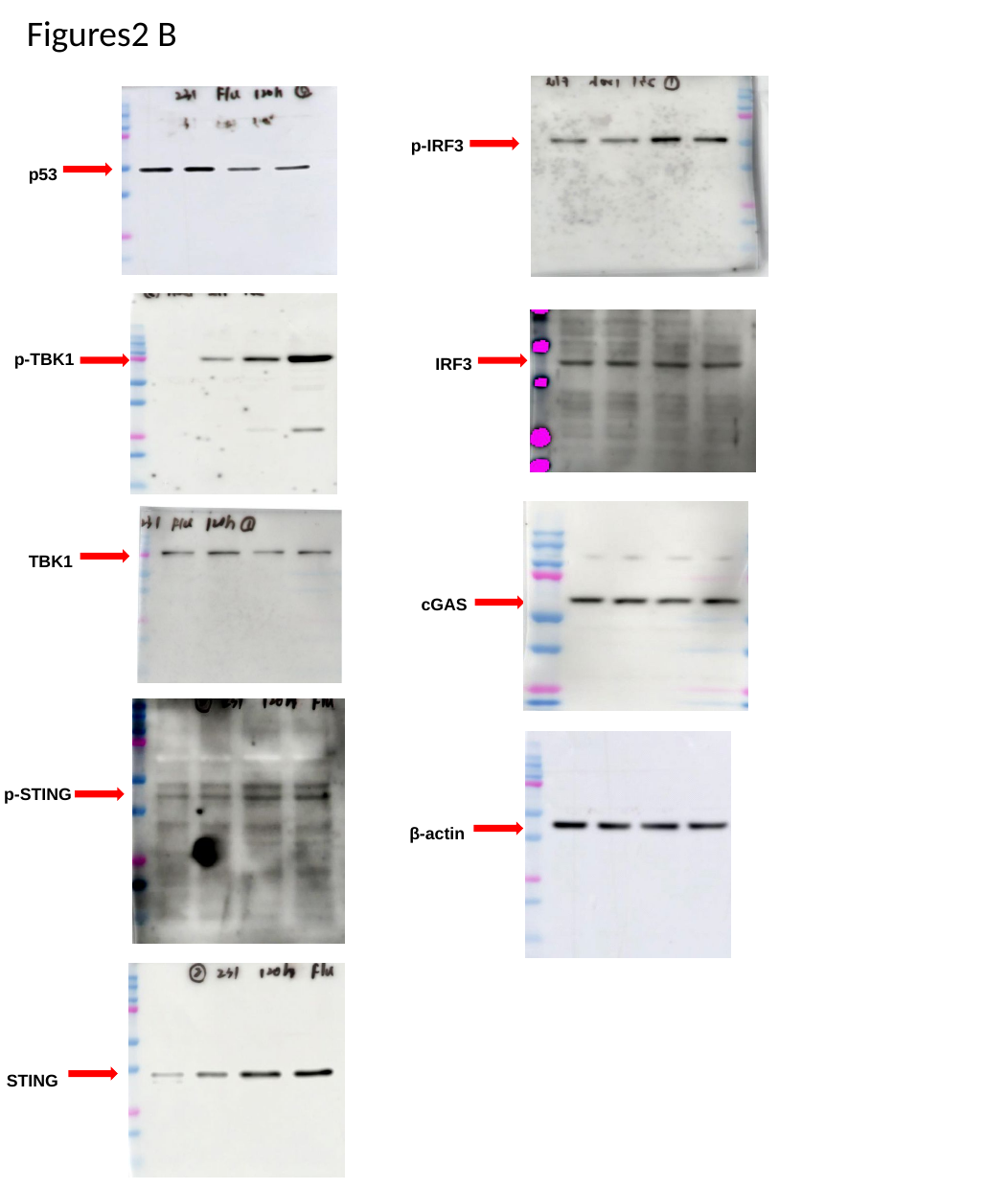

Figures2 B
p-IRF3
p53
IRF3
p-TBK1
TBK1
cGAS
p-STING
β-actin
STING

## Slide 8
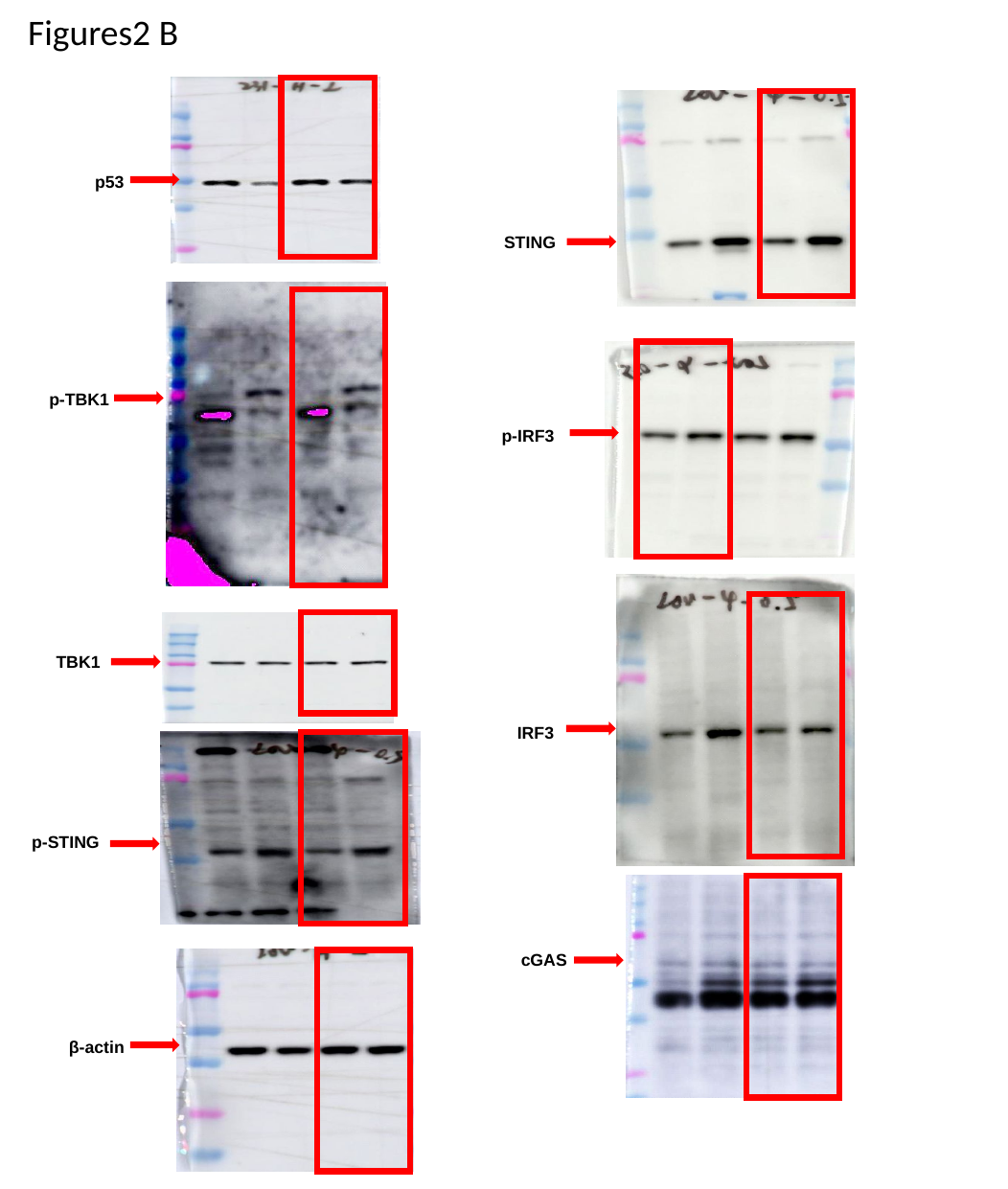

Figures2 B
p53
STING
p-TBK1
p-IRF3
TBK1
IRF3
p-STING
cGAS
β-actin

## Slide 9
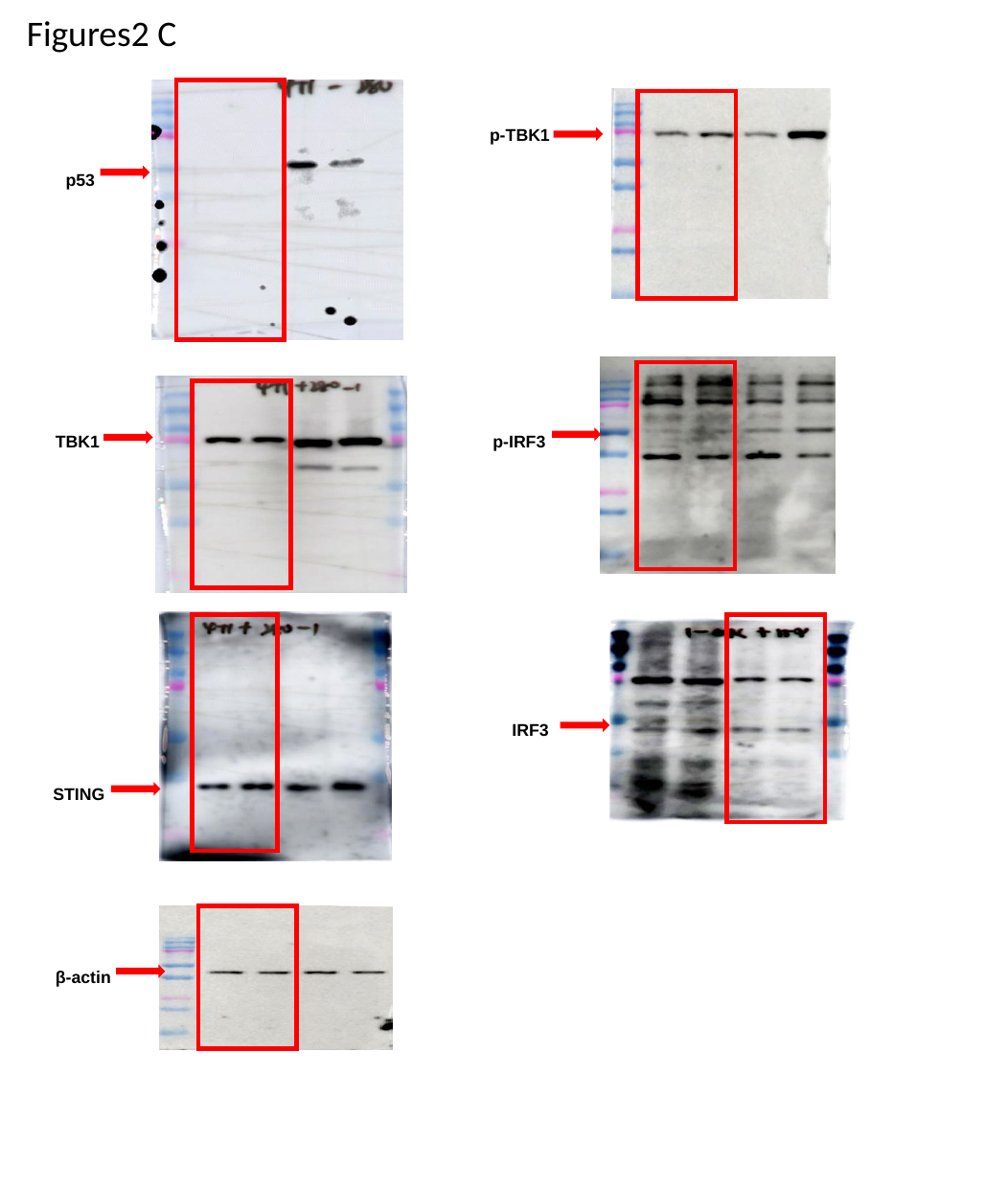

Figures2 C
p-TBK1
p53
TBK1
p-IRF3
IRF3
STING
β-actin

## Slide 10
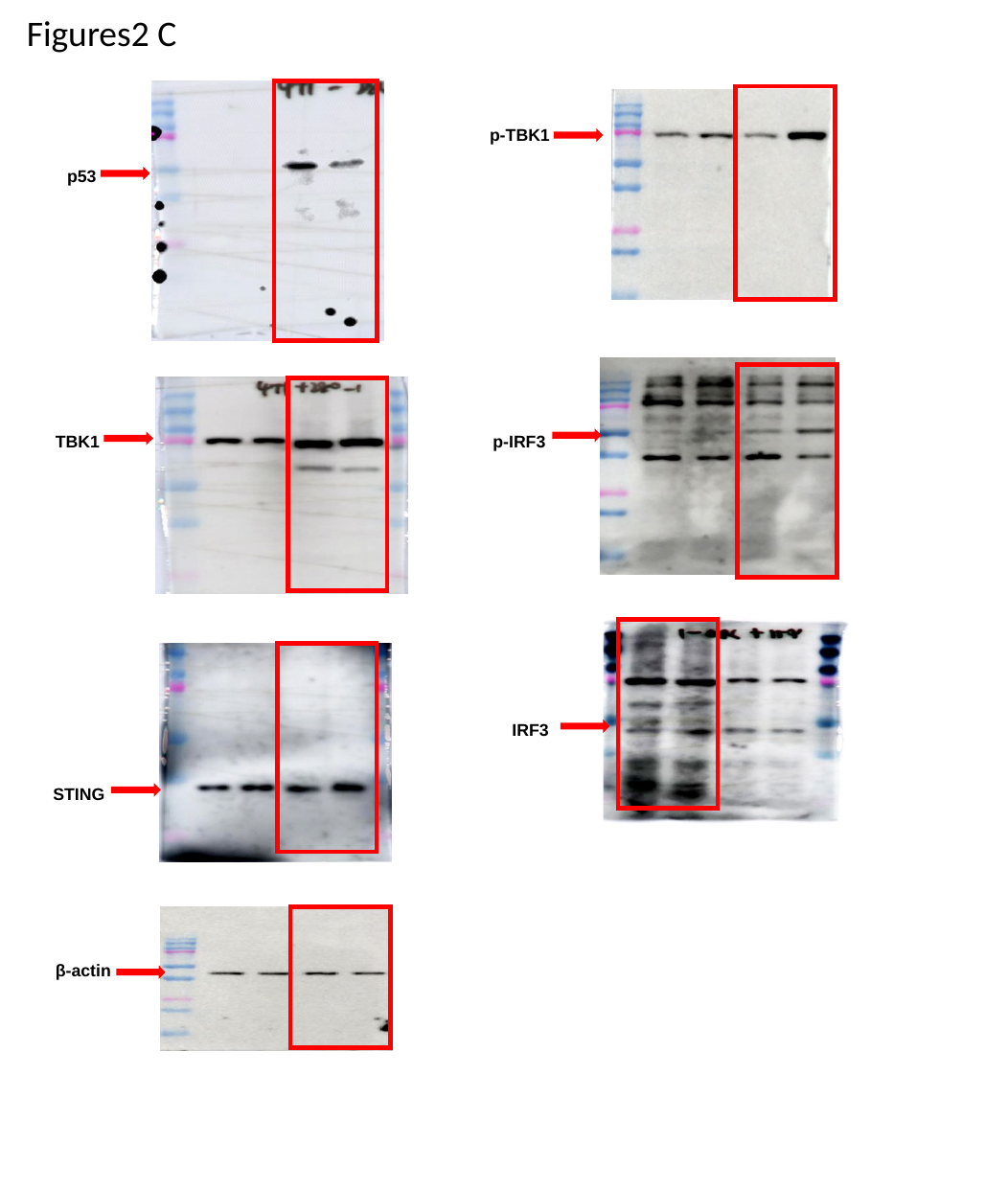

Figures2 C
p-TBK1
p53
TBK1
p-IRF3
IRF3
STING
β-actin

## Slide 11
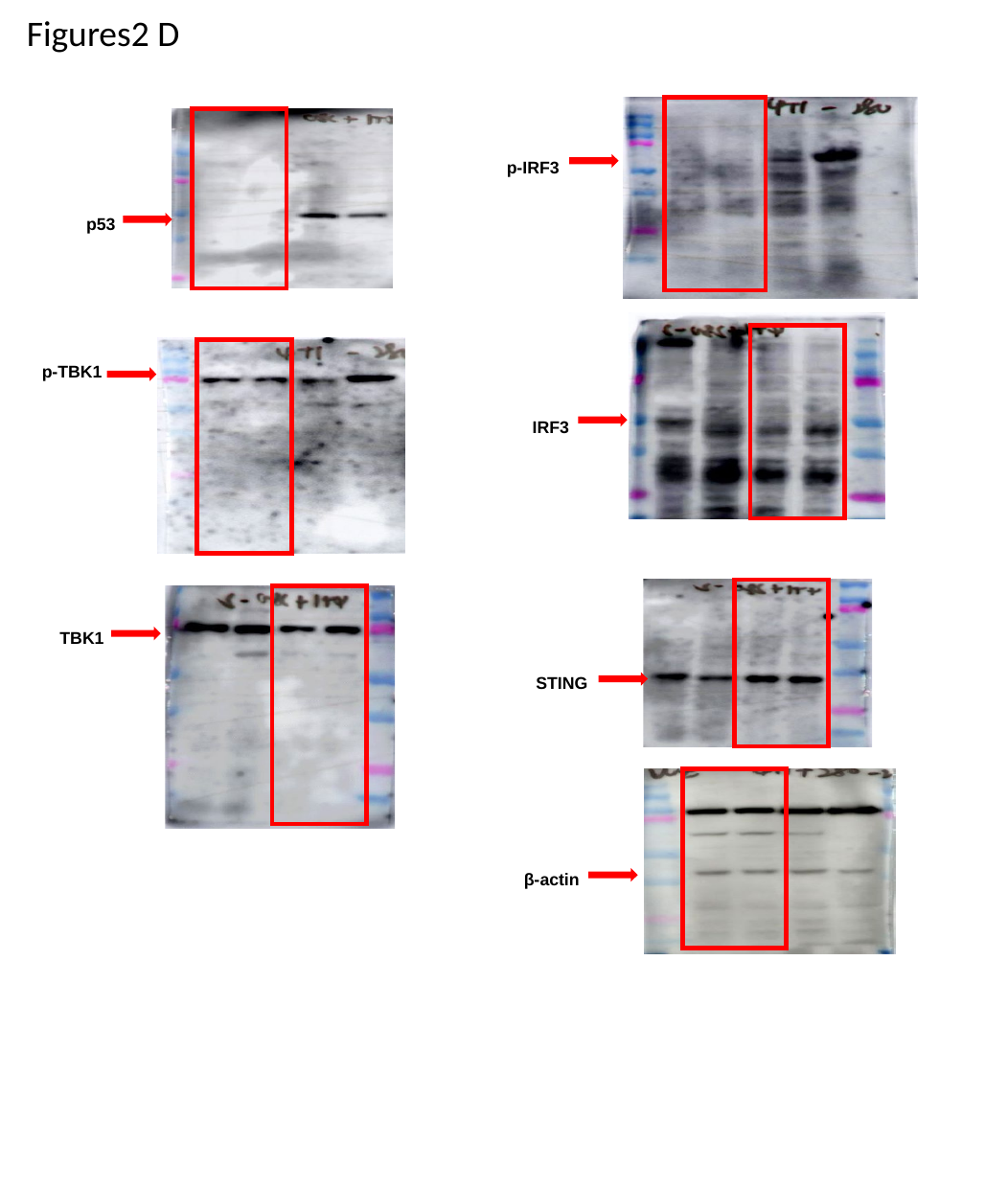

Figures2 D
p-IRF3
p53
p-TBK1
IRF3
TBK1
STING
β-actin

## Slide 12
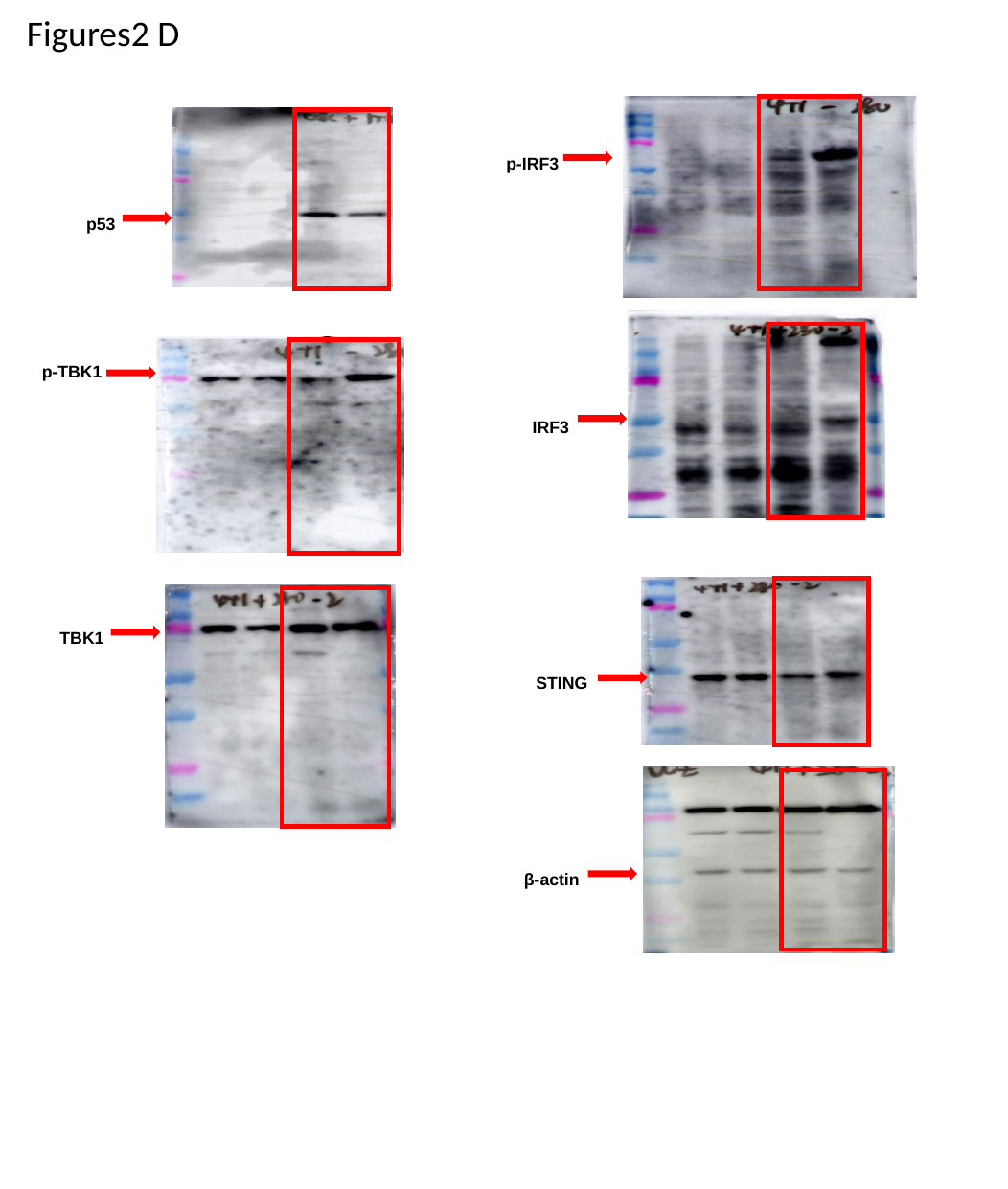

Figures2 D
p-IRF3
p53
p-TBK1
IRF3
TBK1
STING
β-actin

## Slide 13
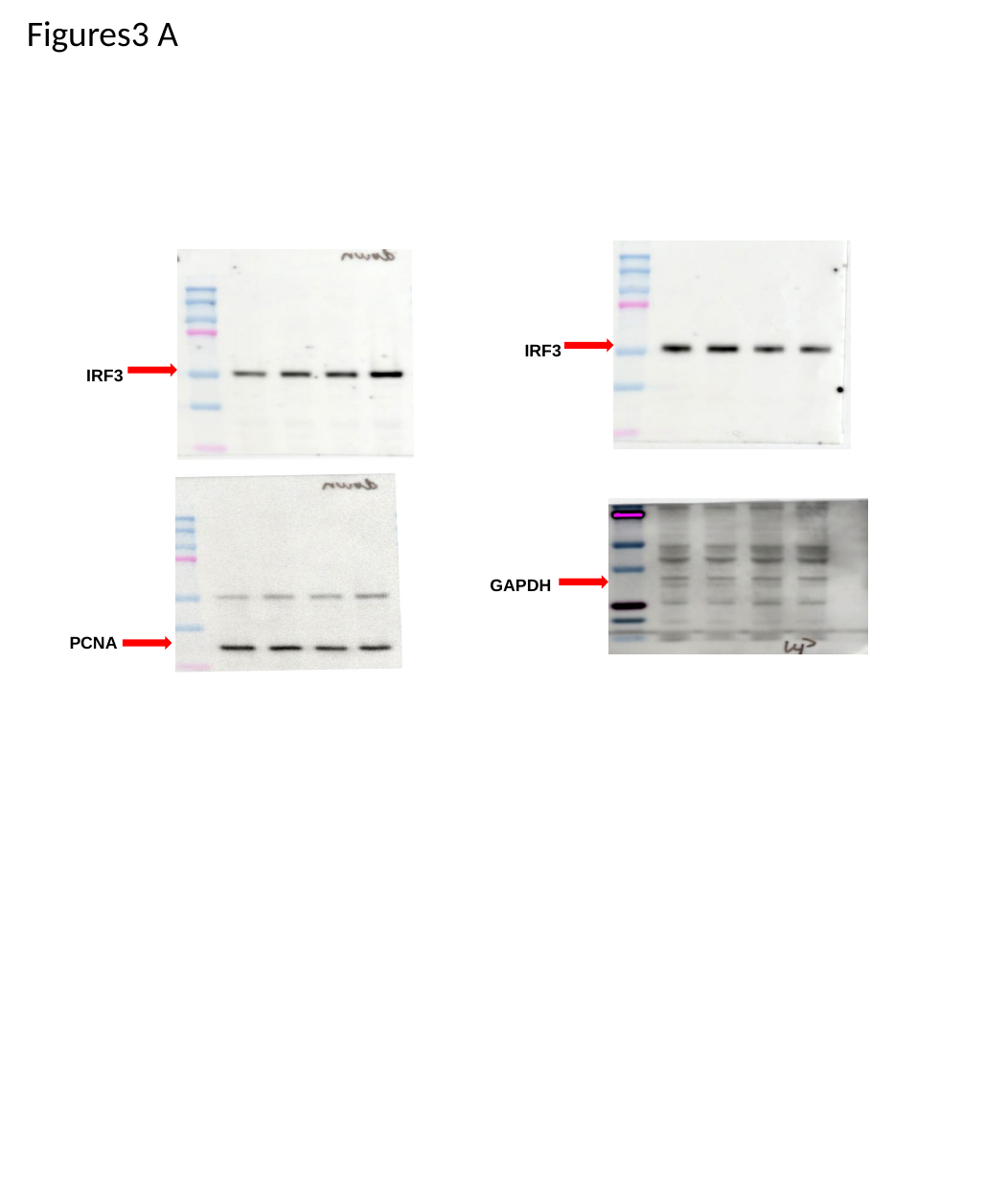

Figures3 A
IRF3
IRF3
GAPDH
PCNA

## Slide 14
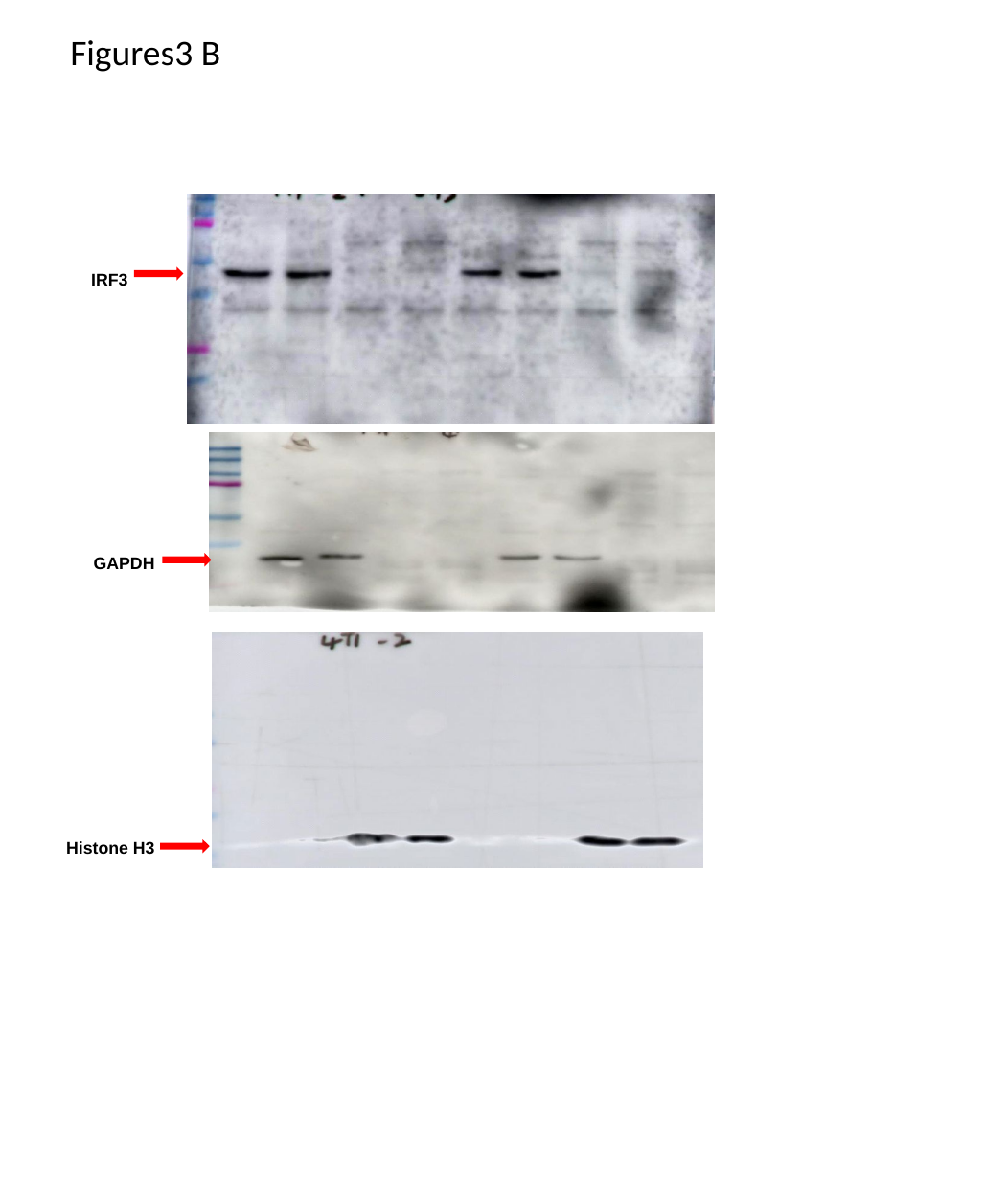

Figures3 B
IRF3
GAPDH
Histone H3

## Slide 15
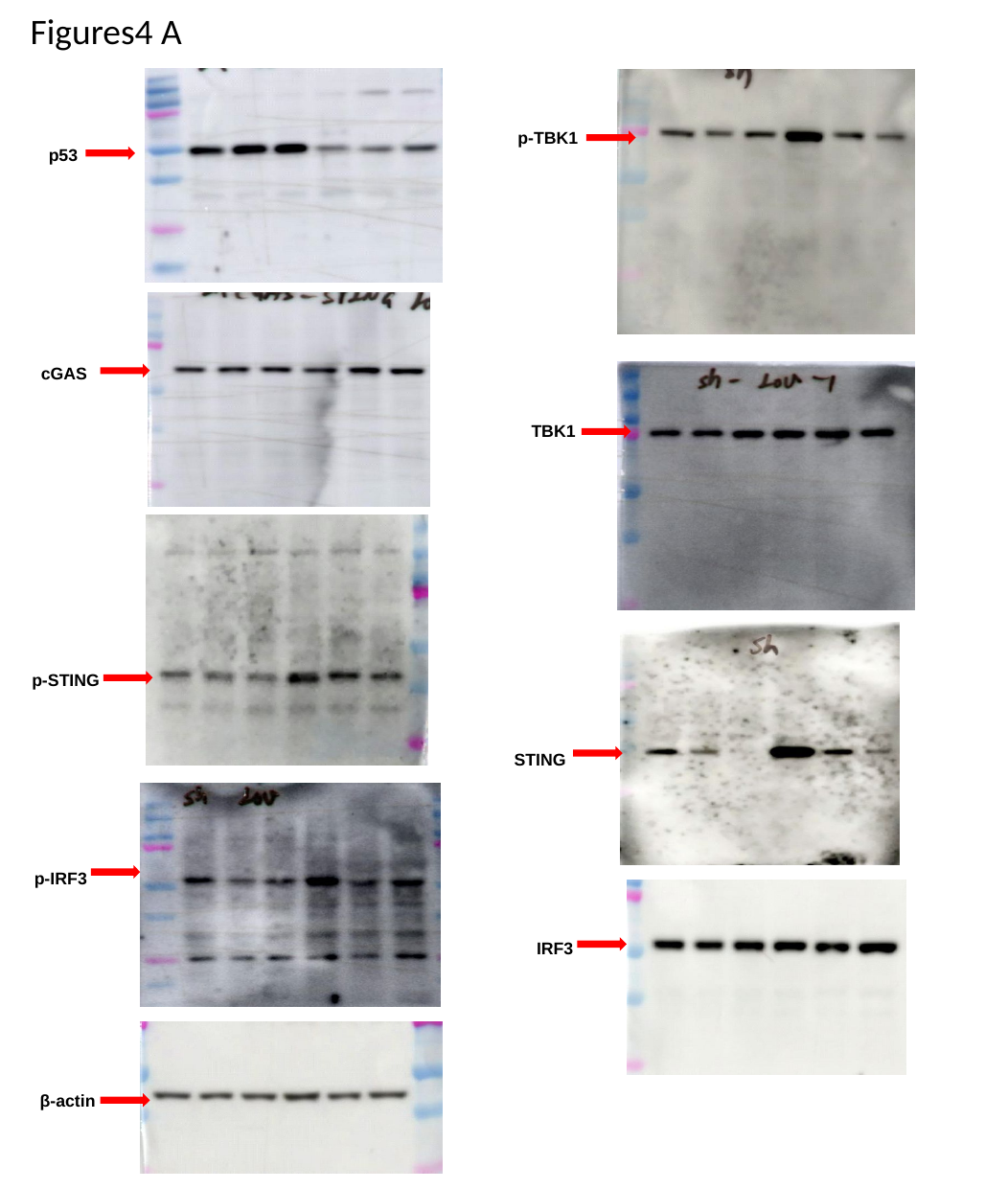

Figures4 A
p-TBK1
p53
cGAS
TBK1
p-STING
STING
p-IRF3
IRF3
β-actin

## Slide 16
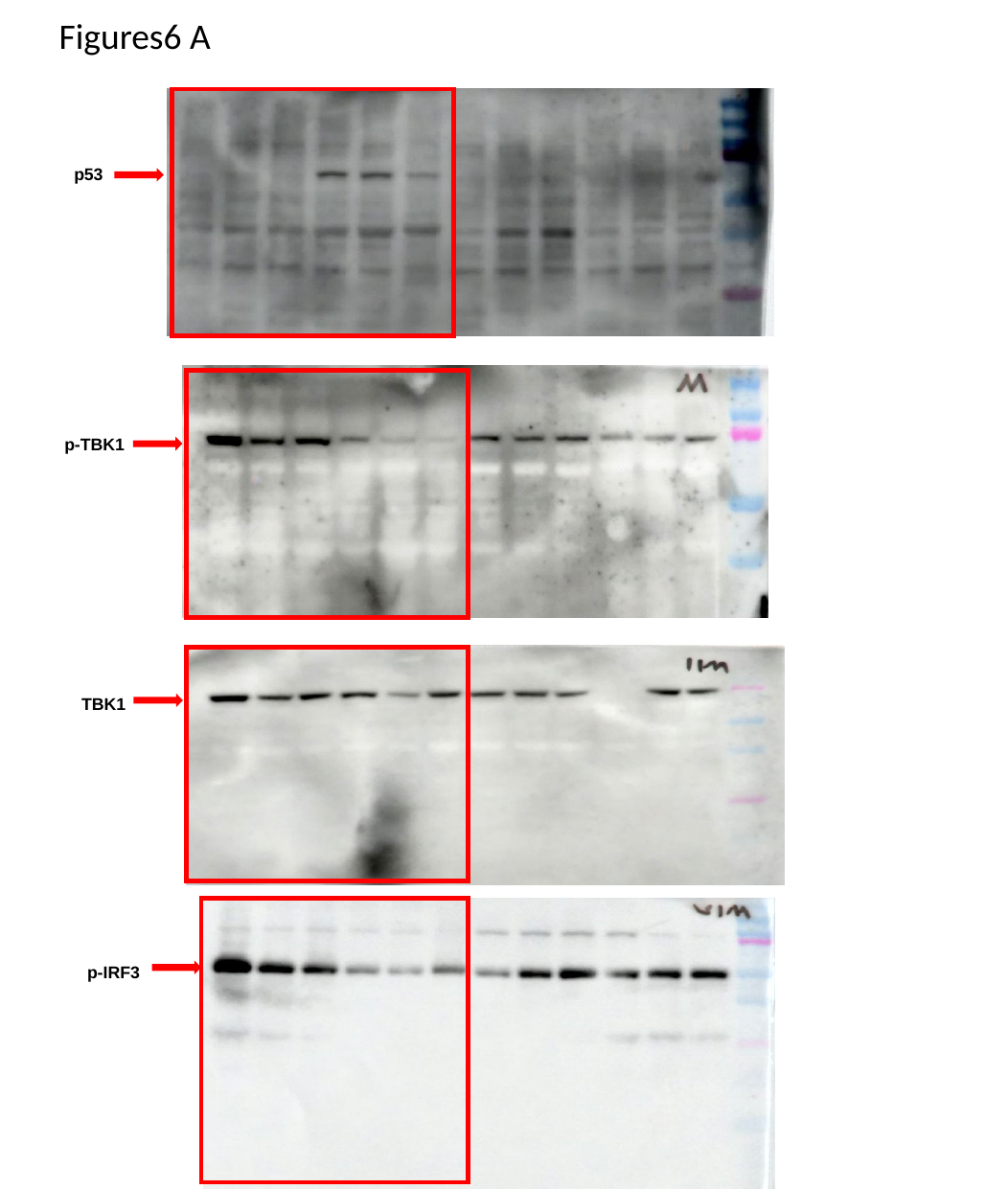

Figures6 A
p53
p-TBK1
TBK1
p-IRF3

## Slide 17
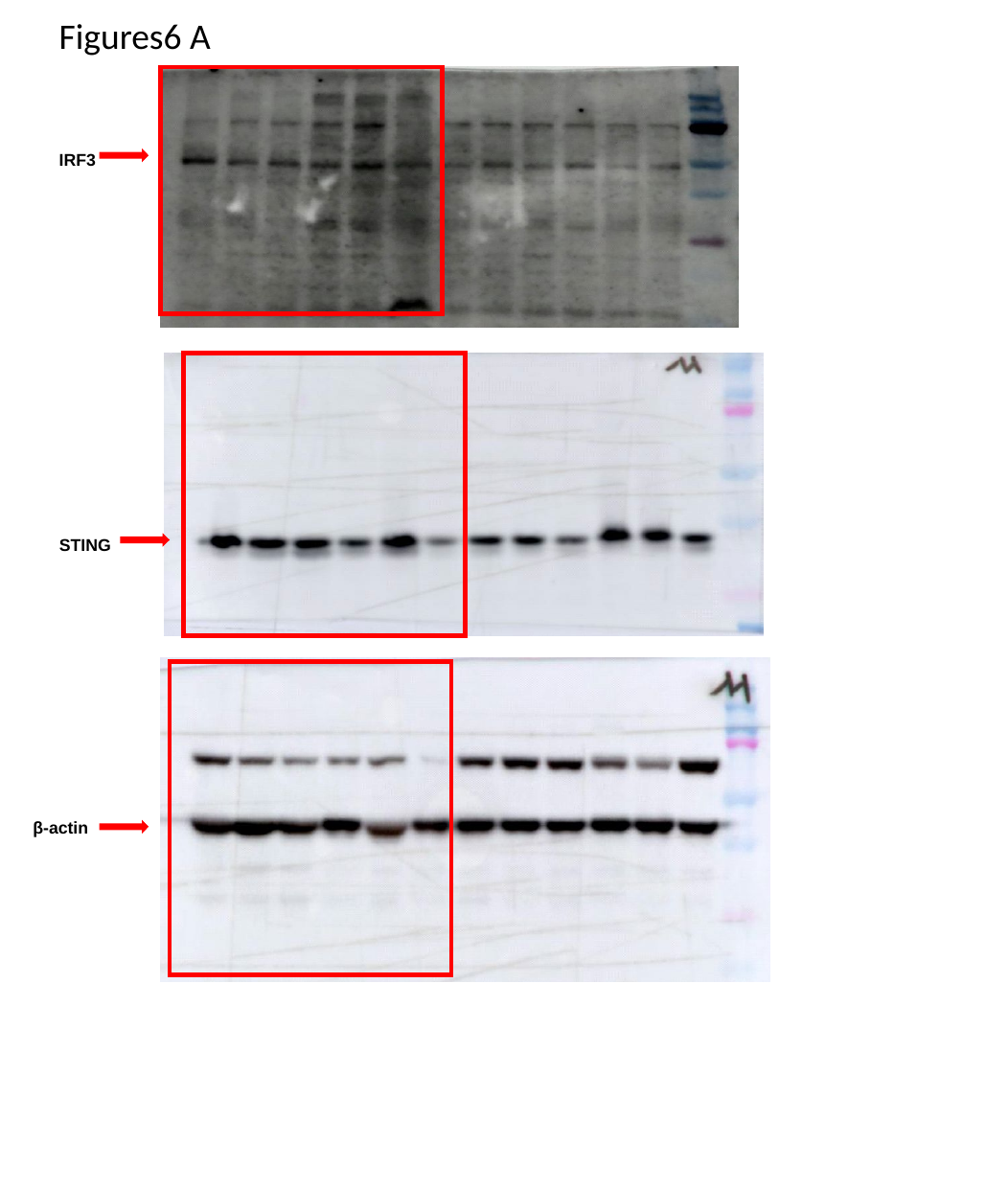

Figures6 A
IRF3
STING
β-actin

## Slide 18
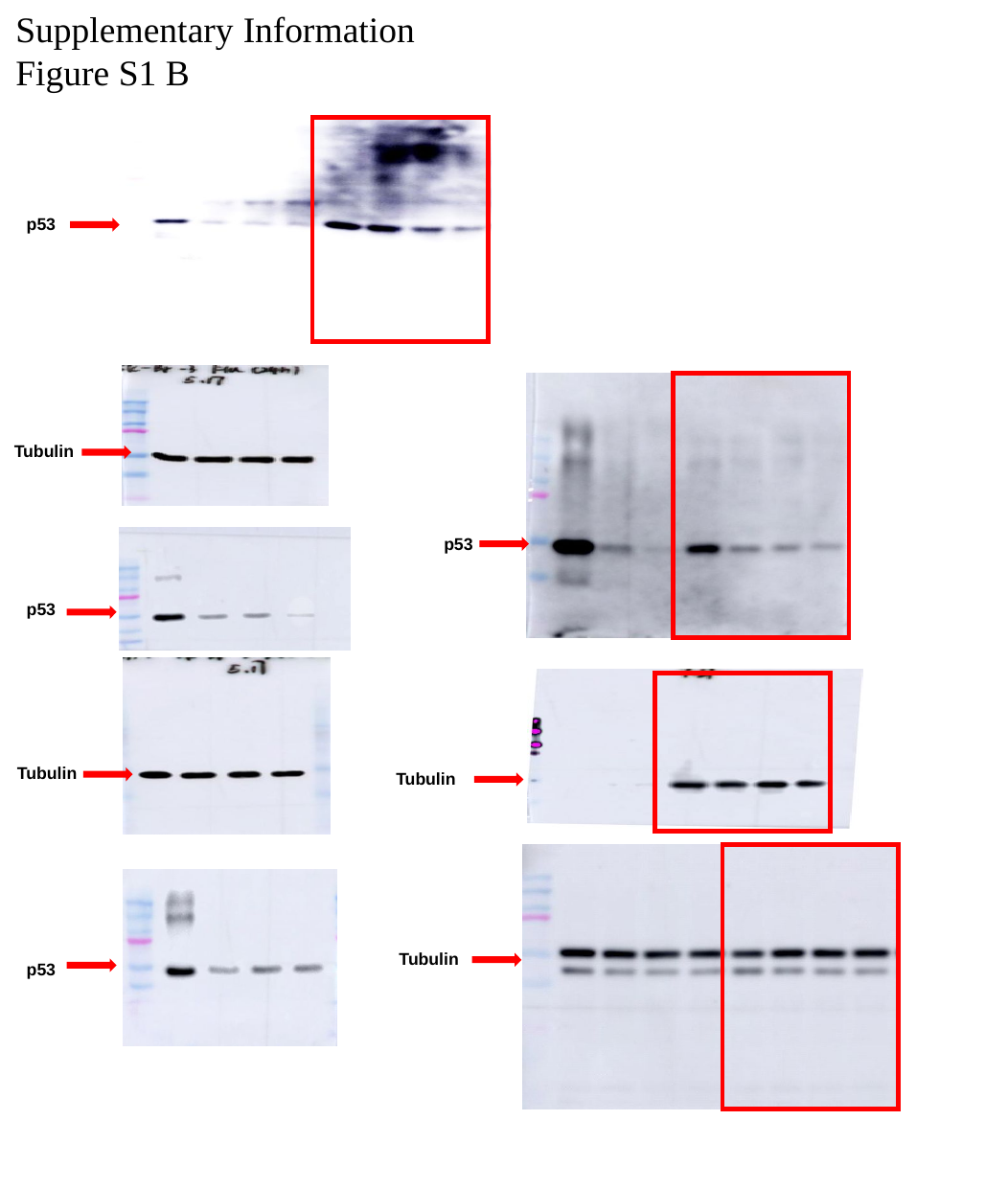

Supplementary Information
Figure S1 B
p53
Tubulin
p53
p53
Tubulin
Tubulin
Tubulin
p53

## Slide 19
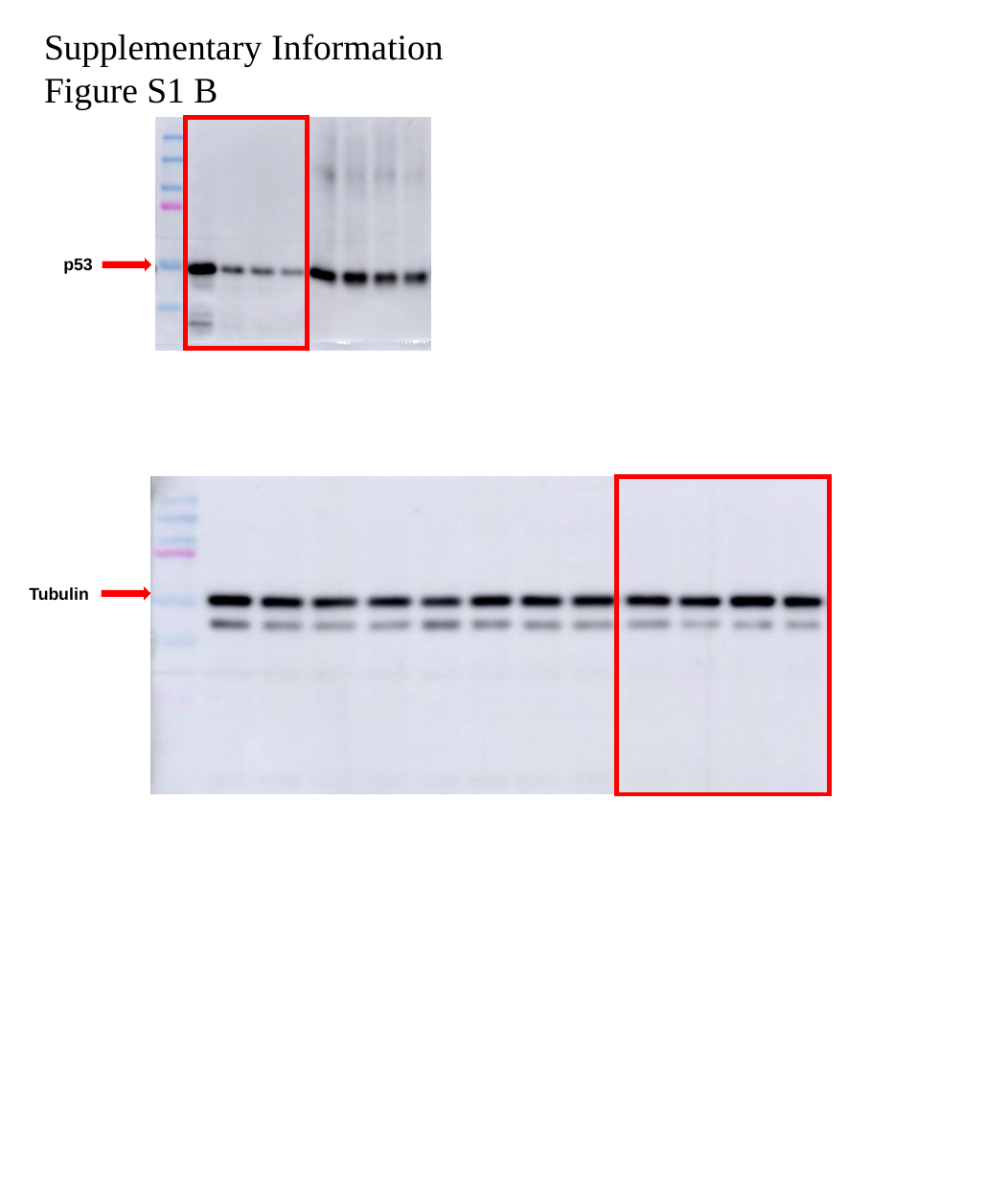

Supplementary Information
Figure S1 B
p53
Tubulin

## Slide 20
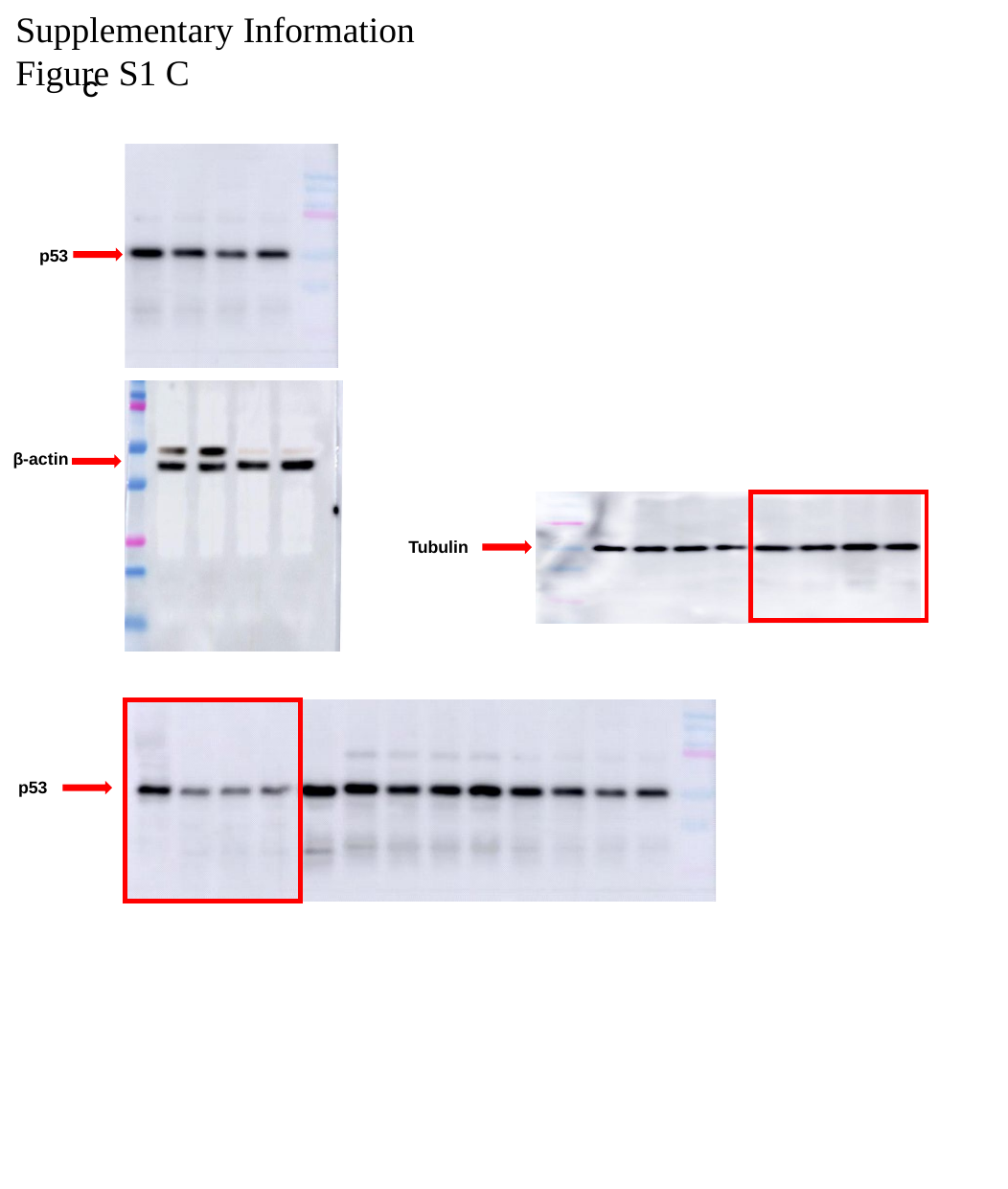

Supplementary Information
Figure S1 C
C
p53
β-actin
Tubulin
p53

## Slide 21
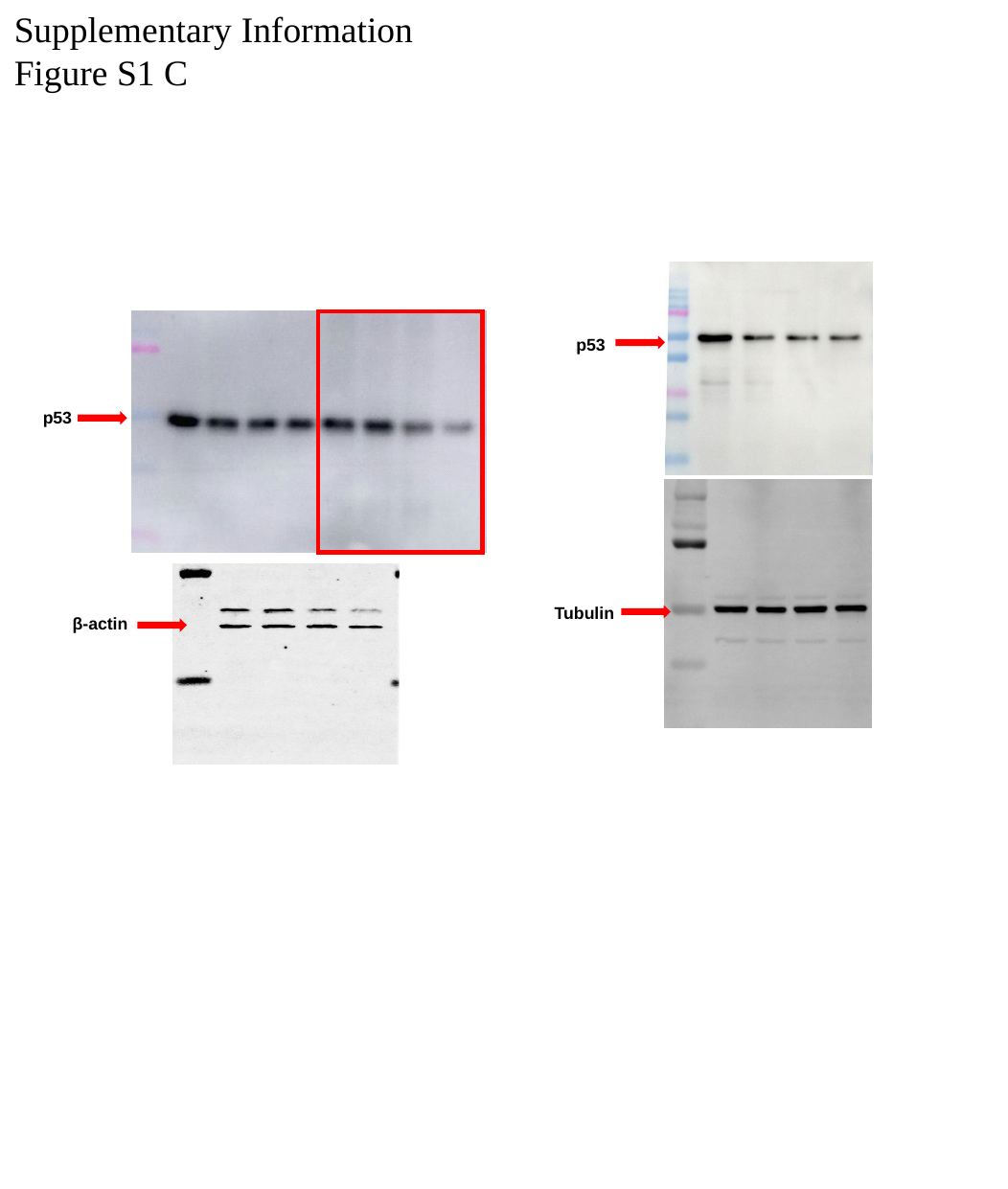

Supplementary Information
Figure S1 C
p53
p53
Tubulin
β-actin

## Slide 22
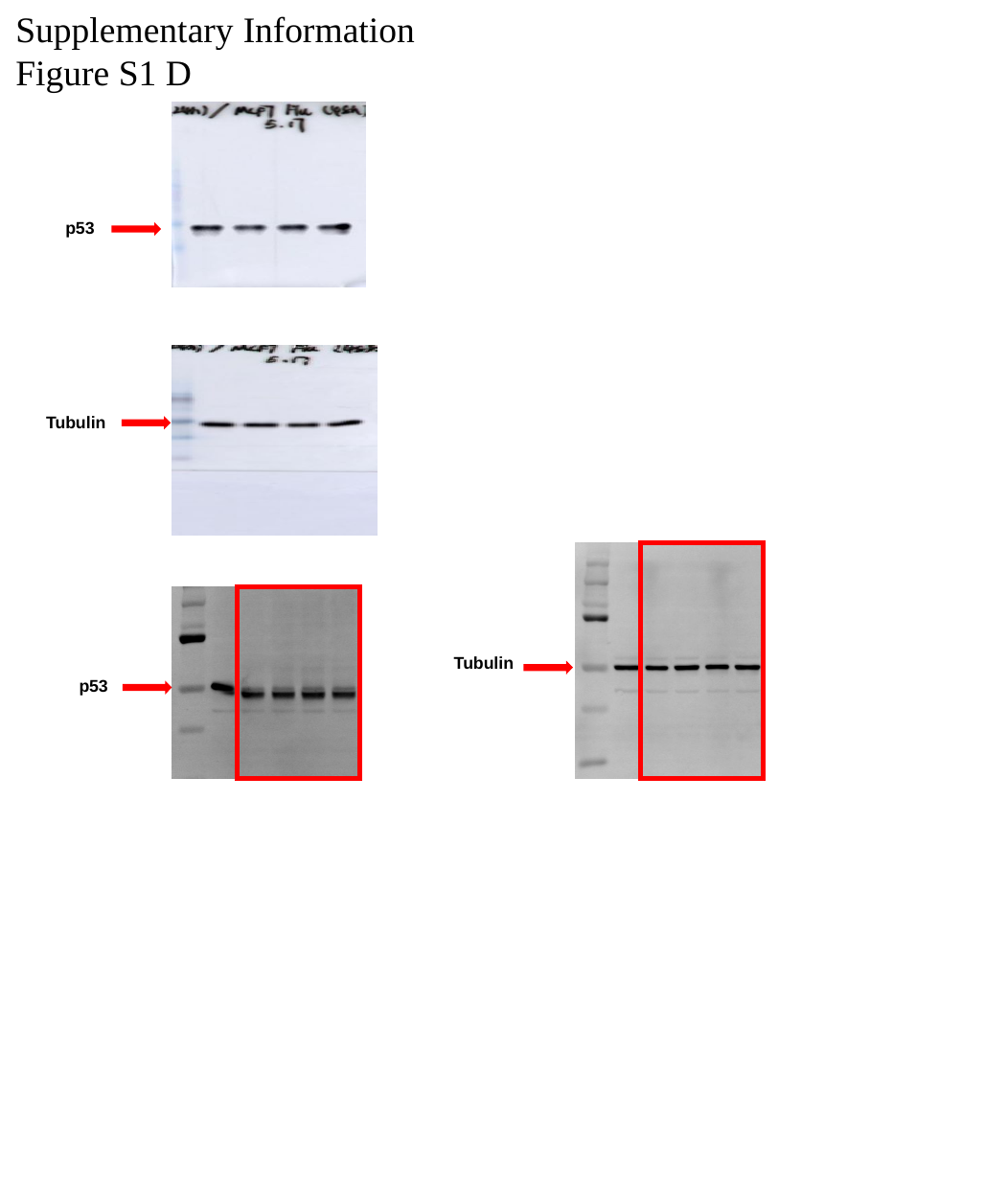

Supplementary Information
Figure S1 D
p53
Tubulin
Tubulin
p53

## Slide 23
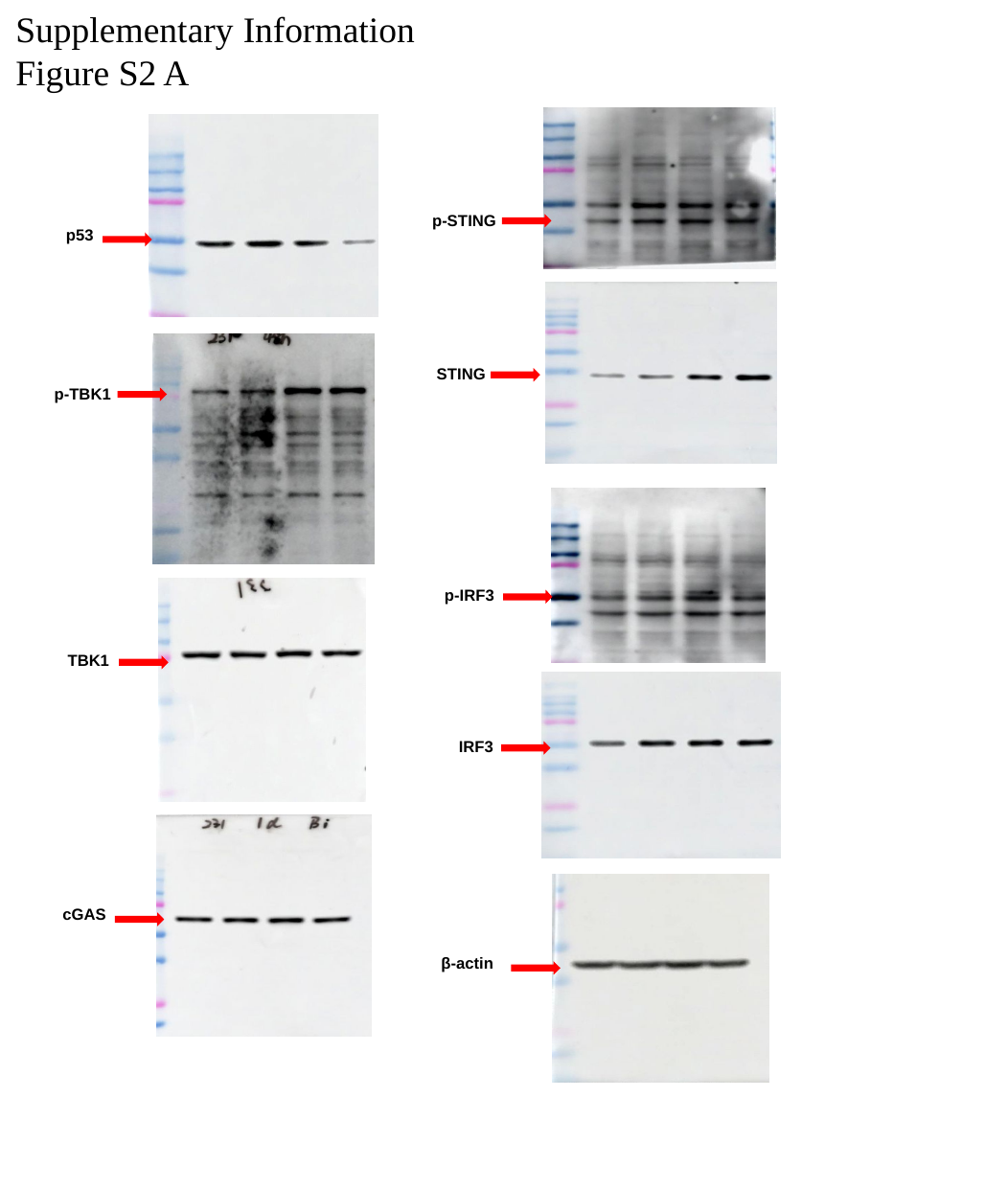

Supplementary Information
Figure S2 A
p-STING
 p53
STING
p-TBK1
p-IRF3
TBK1
 IRF3
 cGAS
 β-actin

## Slide 24
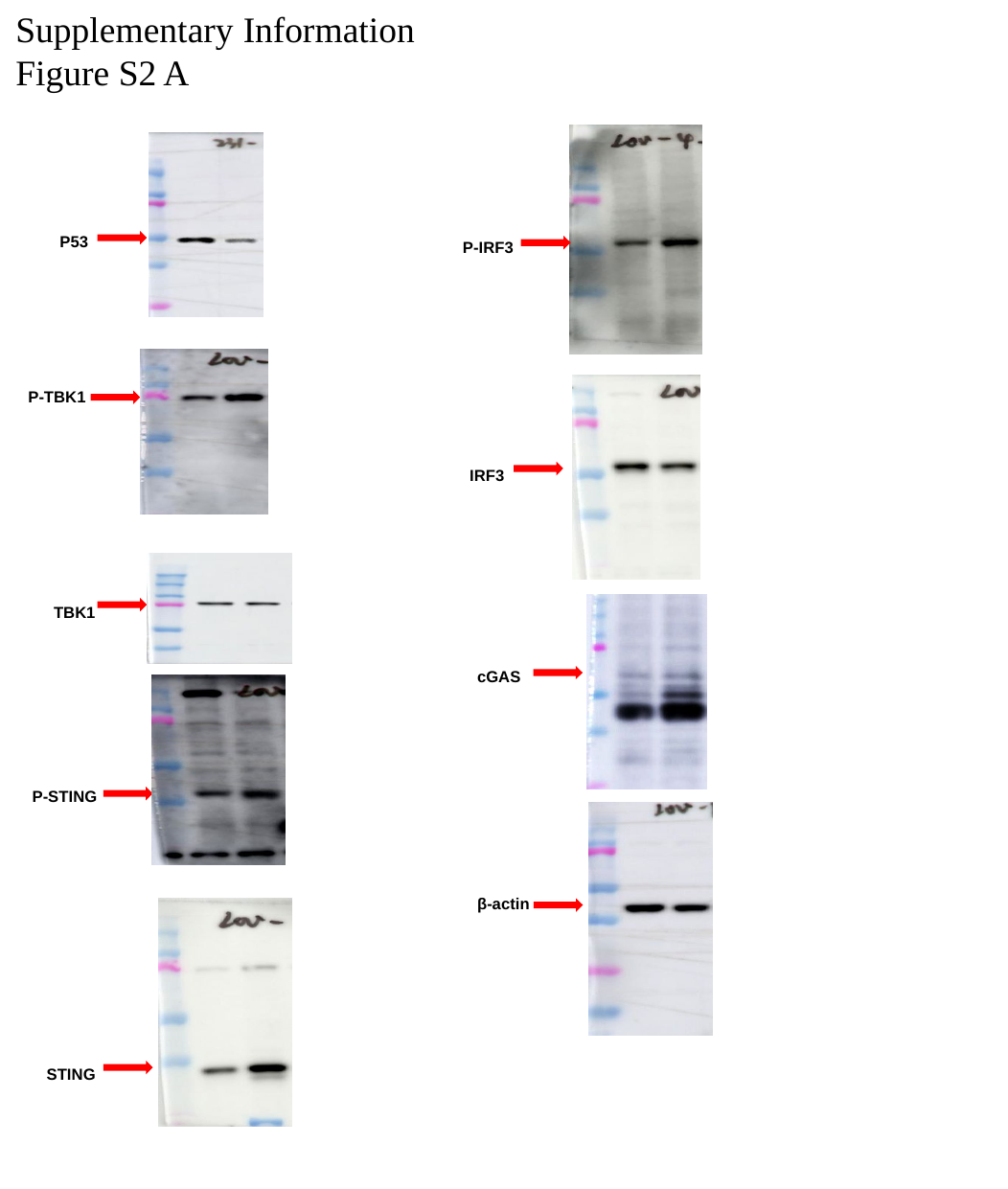

Supplementary Information
Figure S2 A
P53
P-IRF3
P-TBK1
IRF3
TBK1
cGAS
P-STING
β-actin
STING

## Slide 25
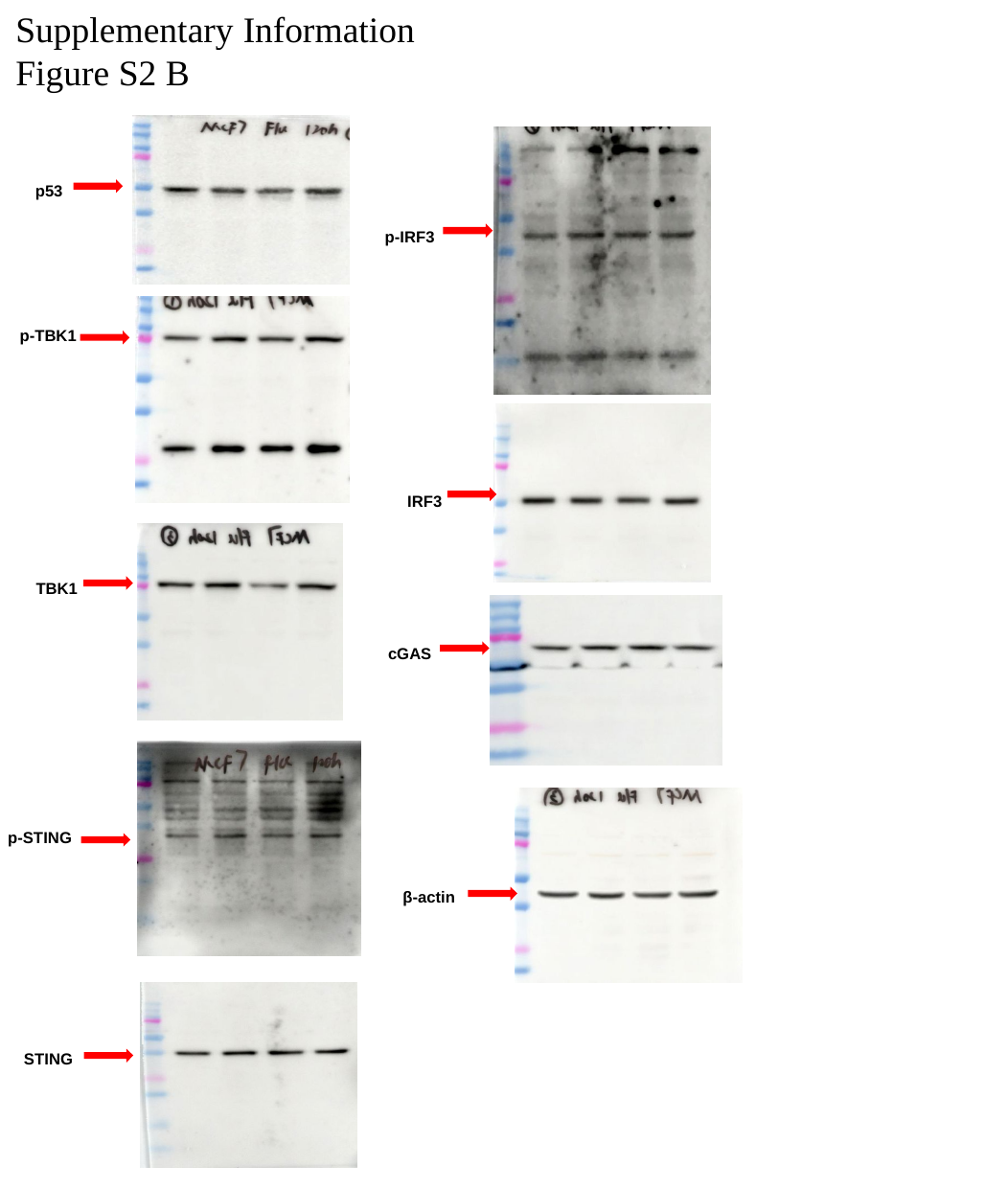

Supplementary Information
Figure S2 B
p53
p-IRF3
p-TBK1
IRF3
TBK1
cGAS
p-STING
β-actin
STING
