# Supplementary material for: Low-dose statins restore innate immune response in breast cancer cells via suppression of mutant p53
Source: Front Pharmacol. 2025 May 2;16:1492305. doi: 10.3389/fphar.2025.1492305 (PMC12081456; doi:10.3389/fphar.2025.1492305)

Figures1 C

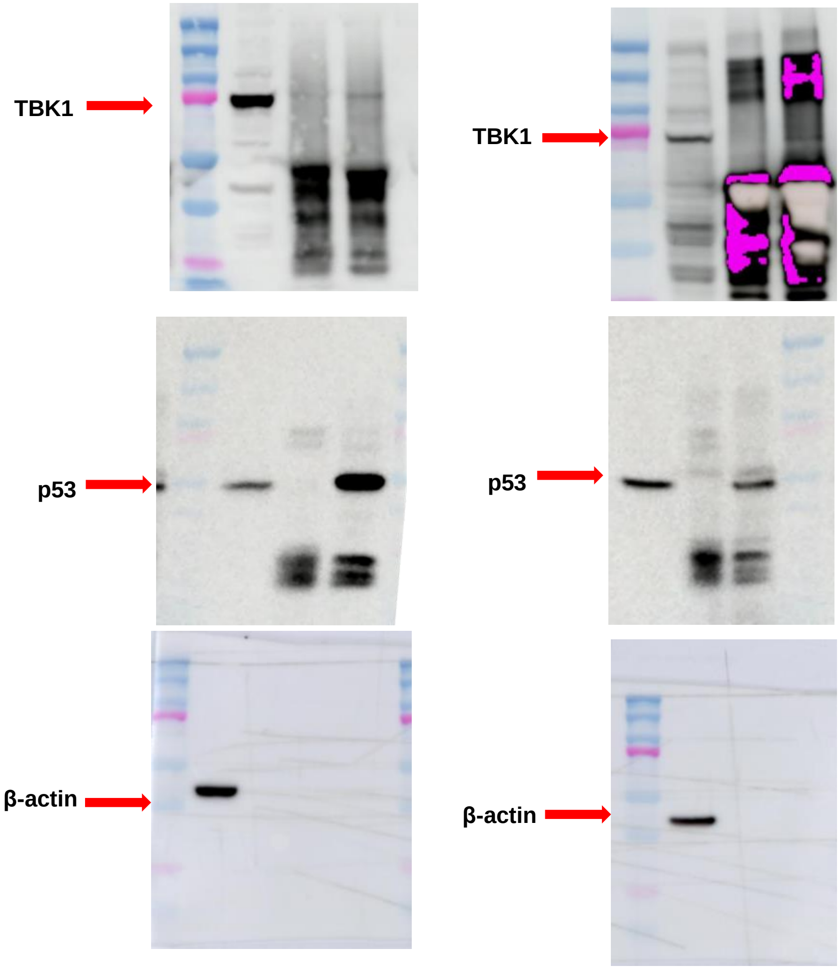

Figures1 D

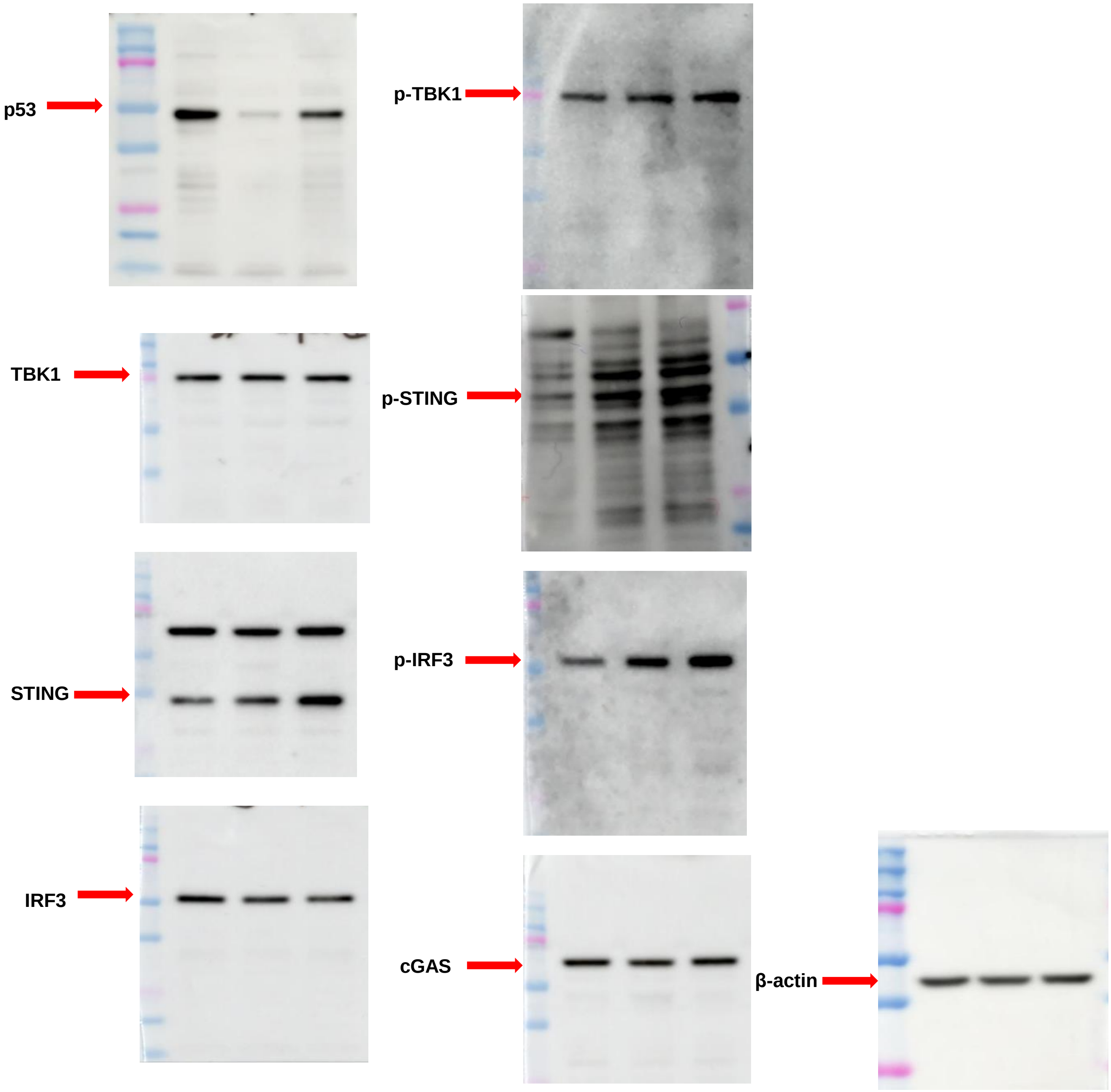

Figures1 D

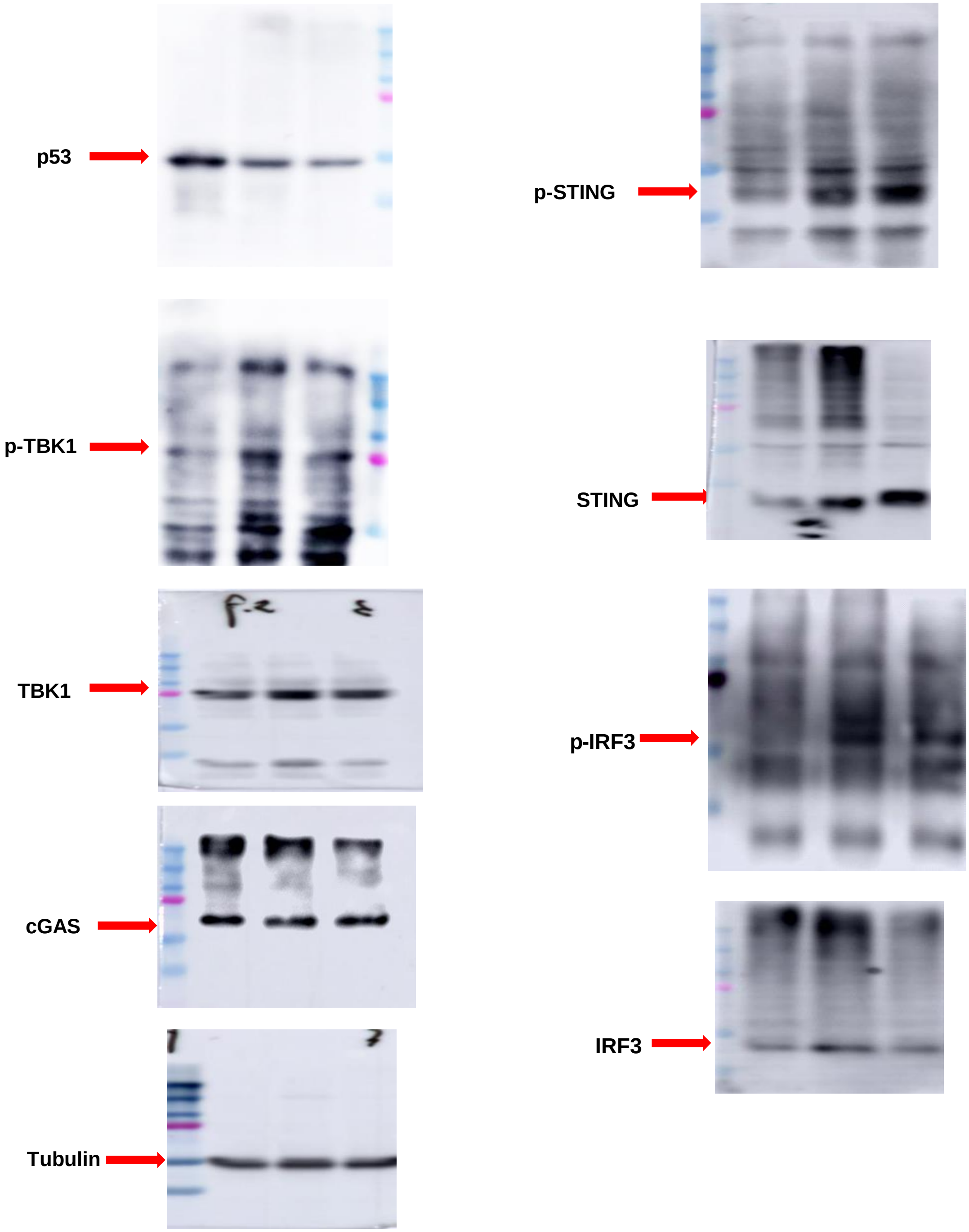

Figures1 E

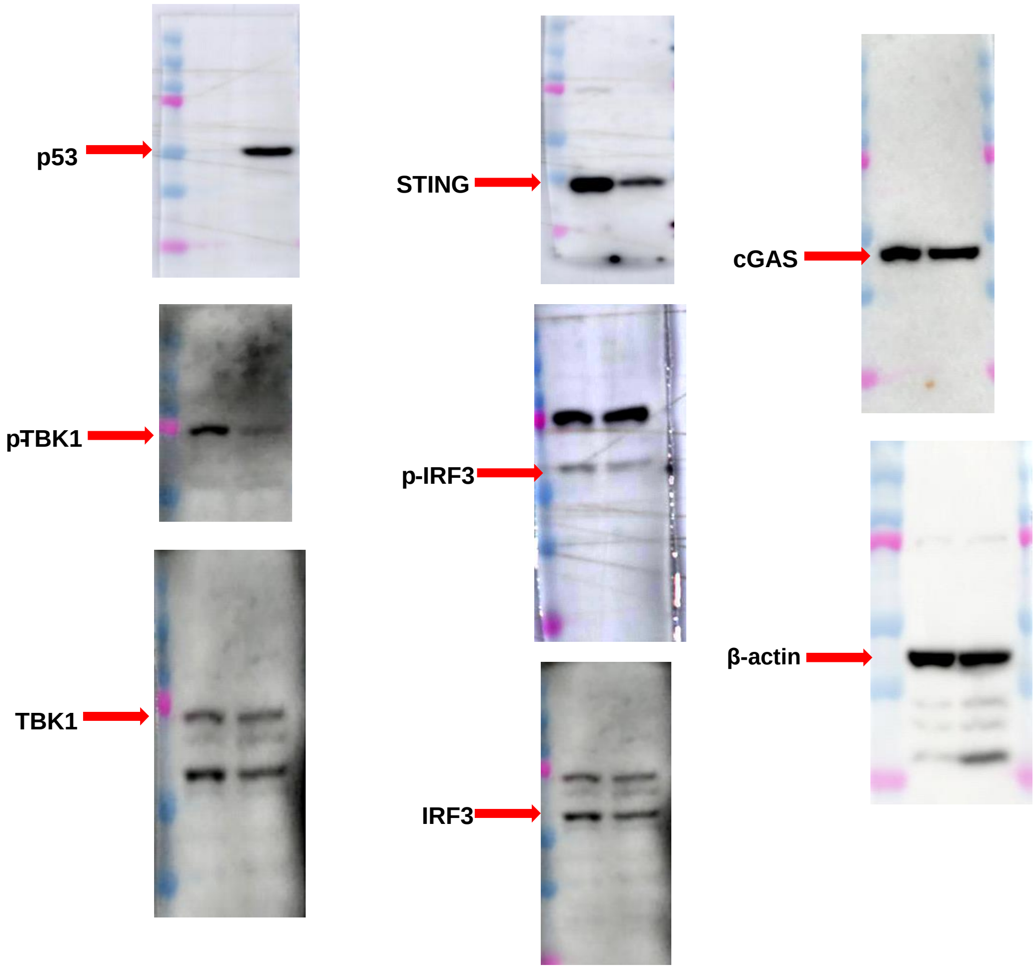

Figures2 A

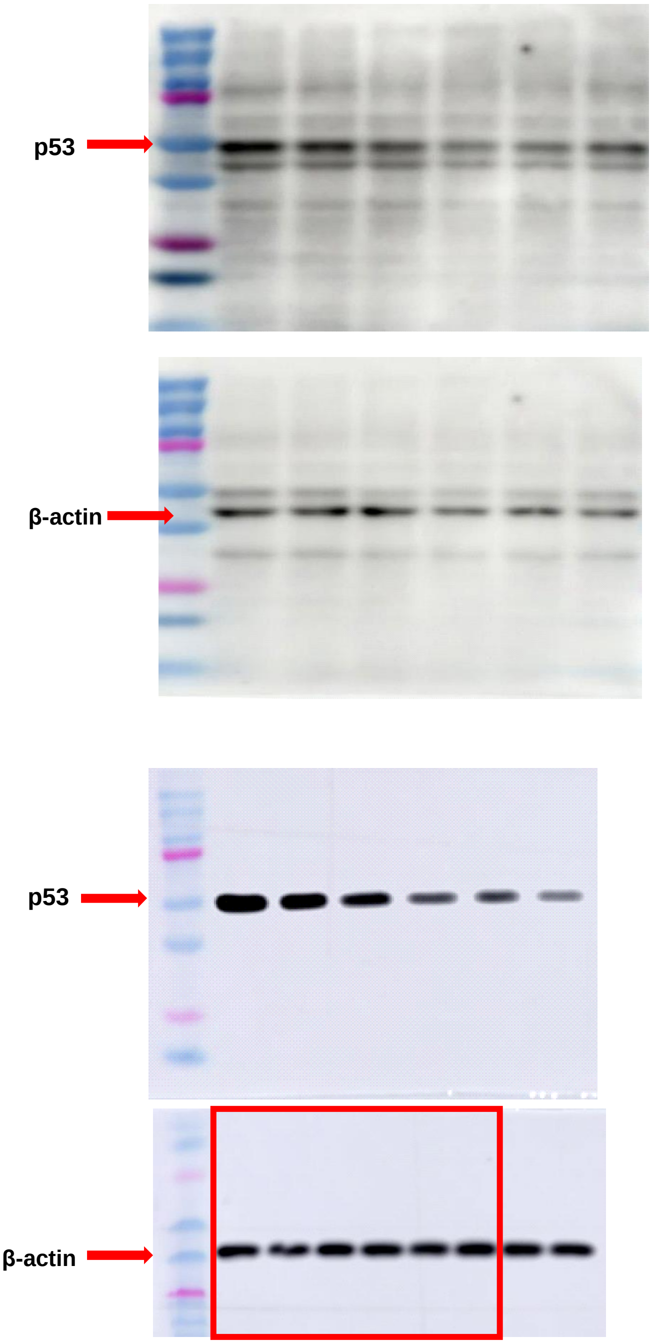

Figures2 B

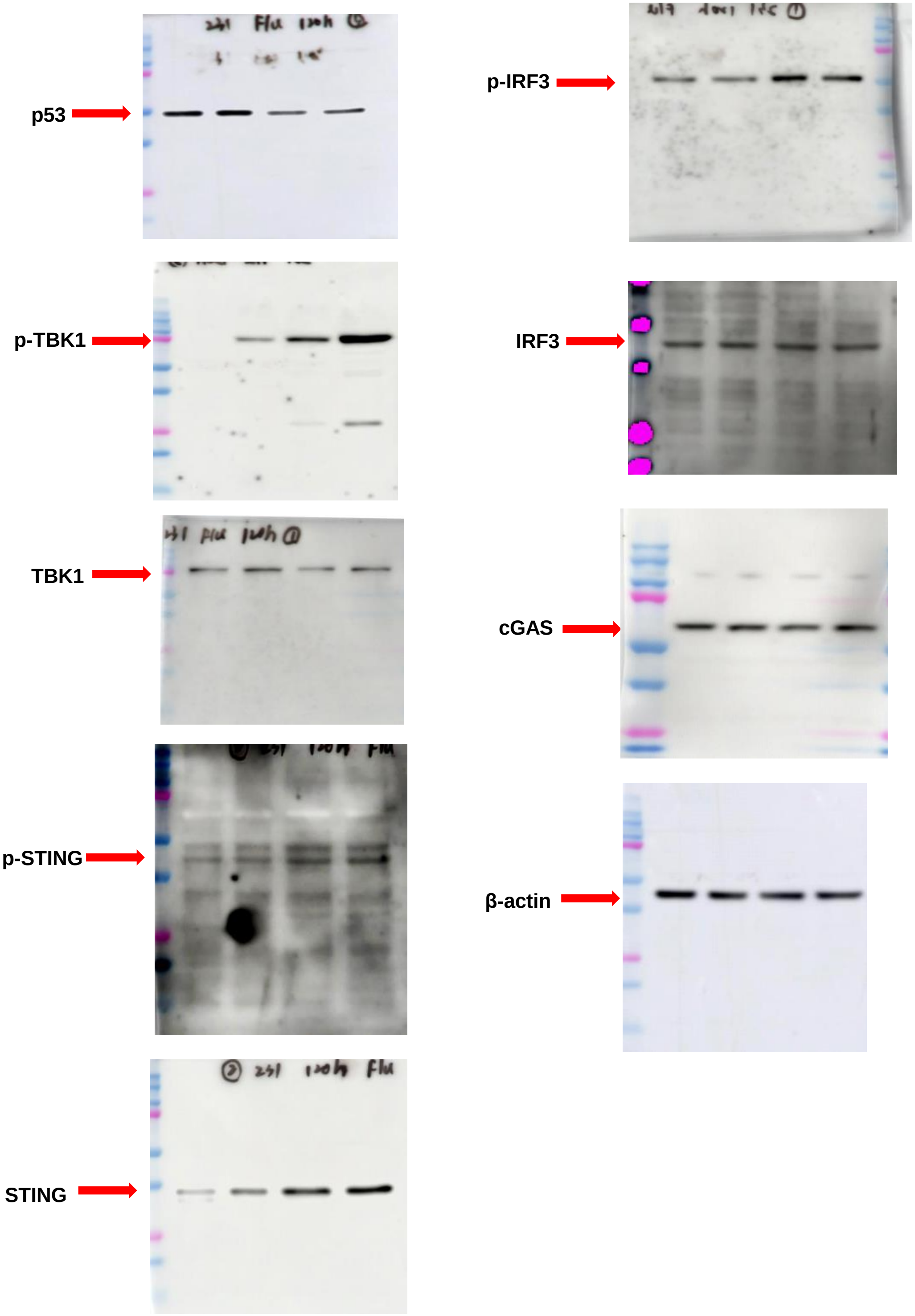

Figures2 B

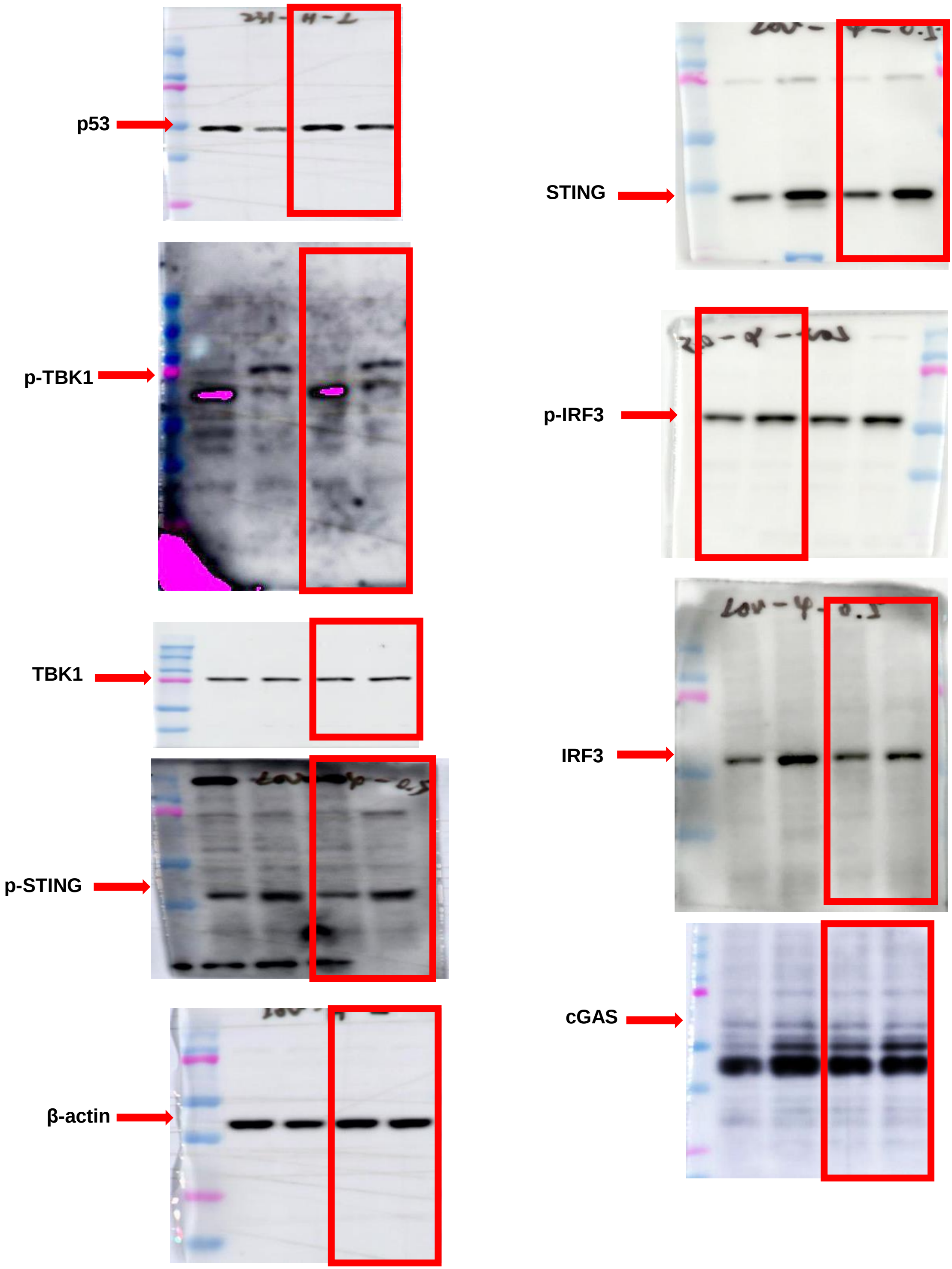

Figures2 C

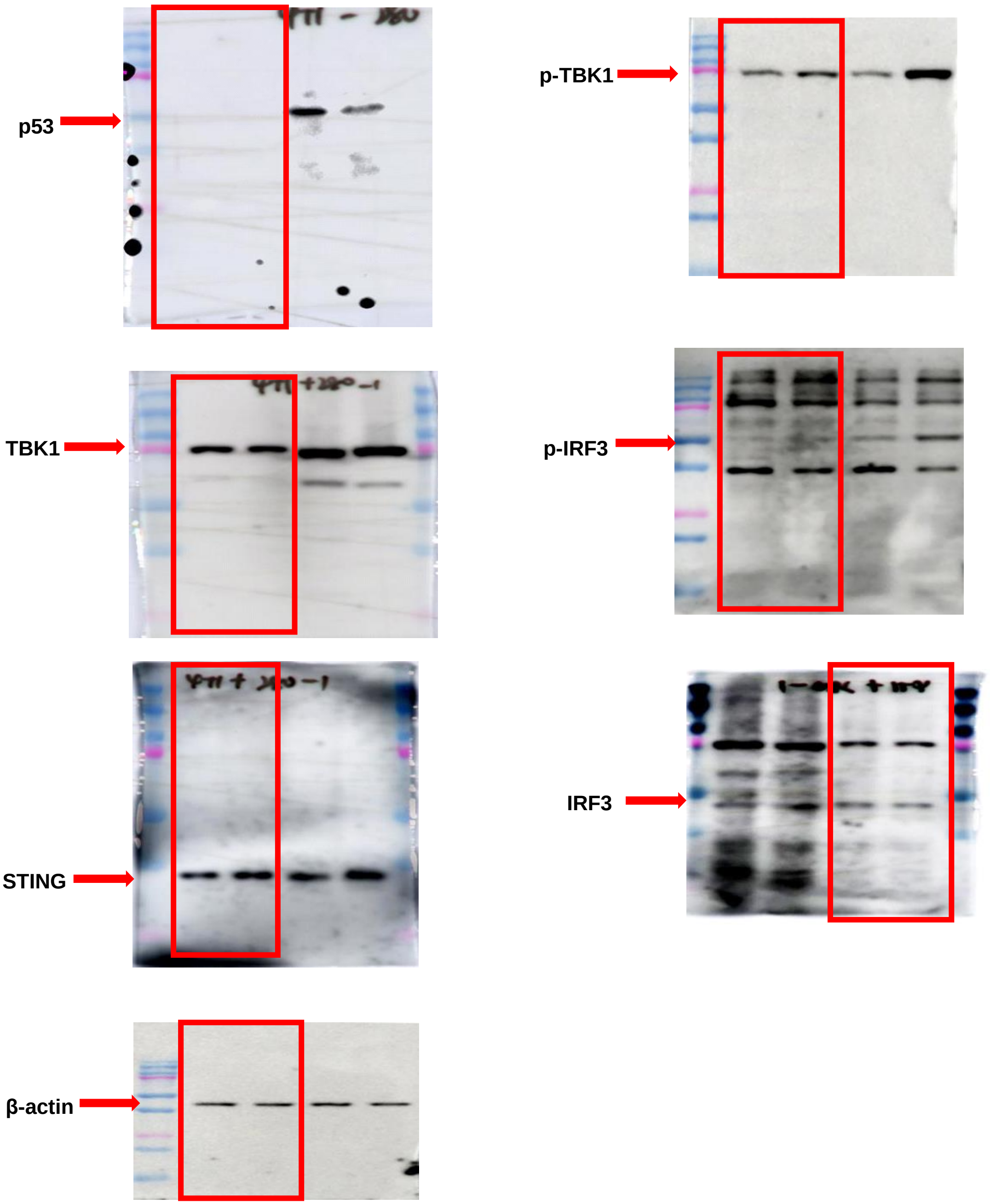

Figures2 C

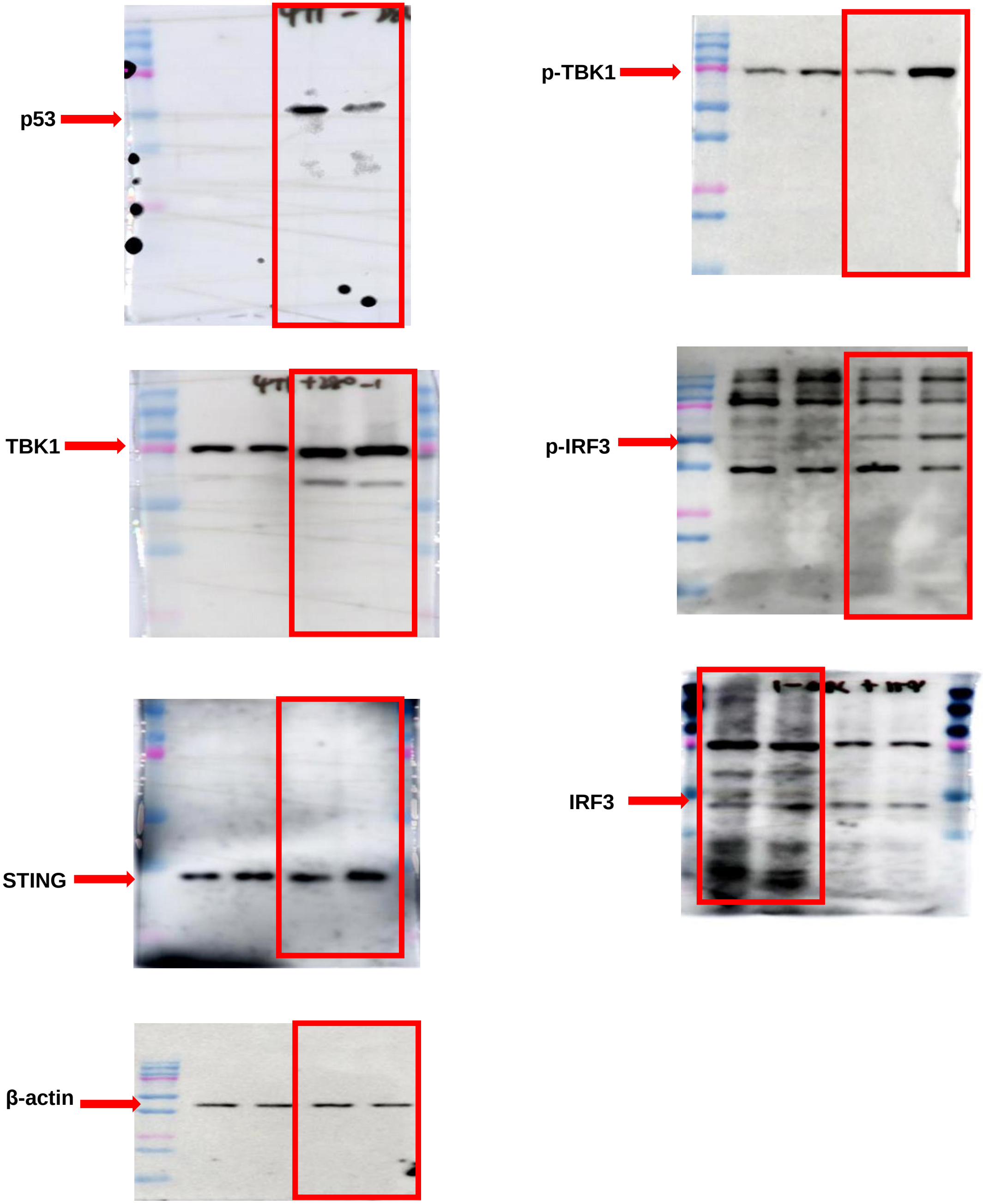

Figures2 D

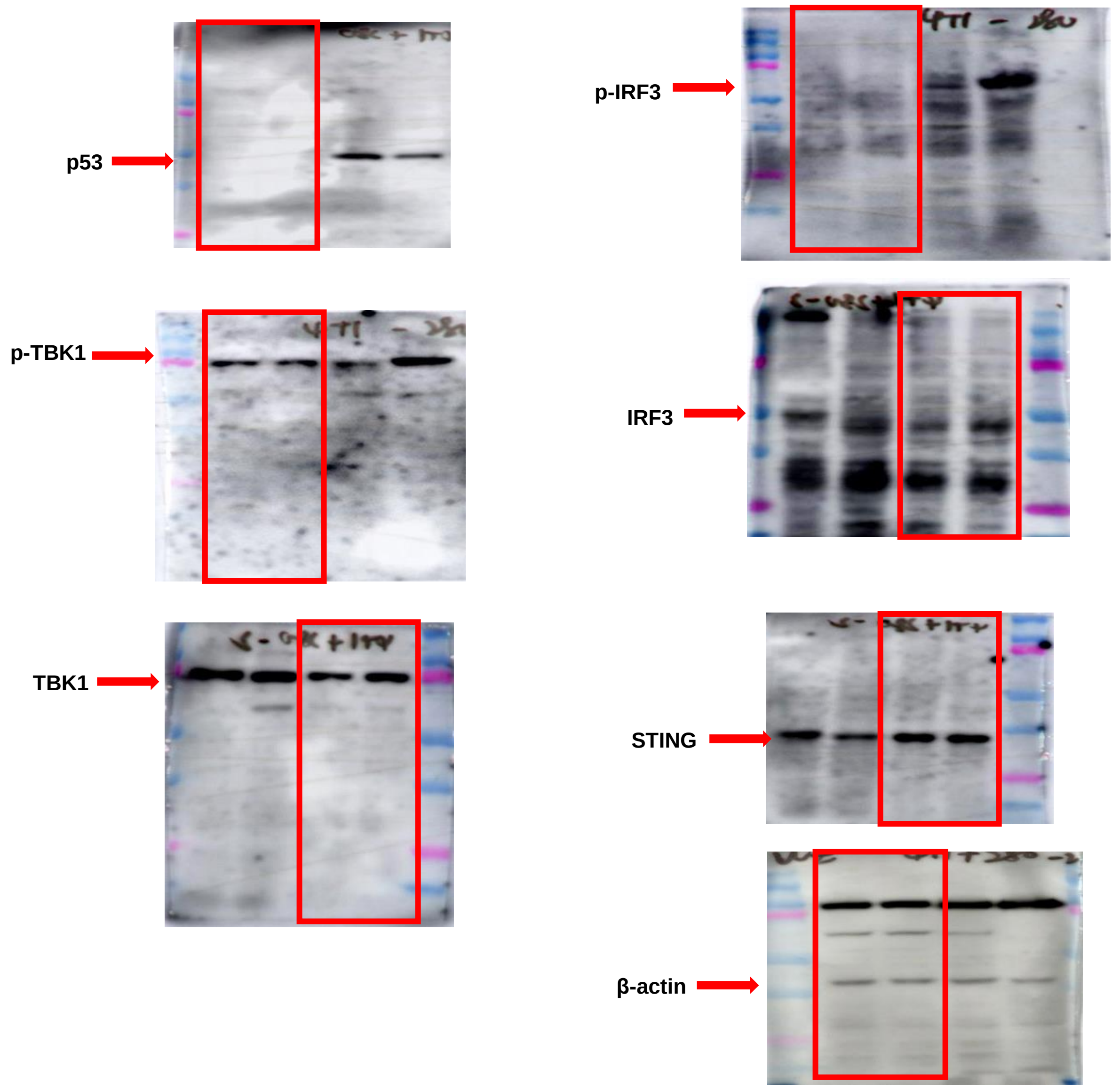

Figures2 D

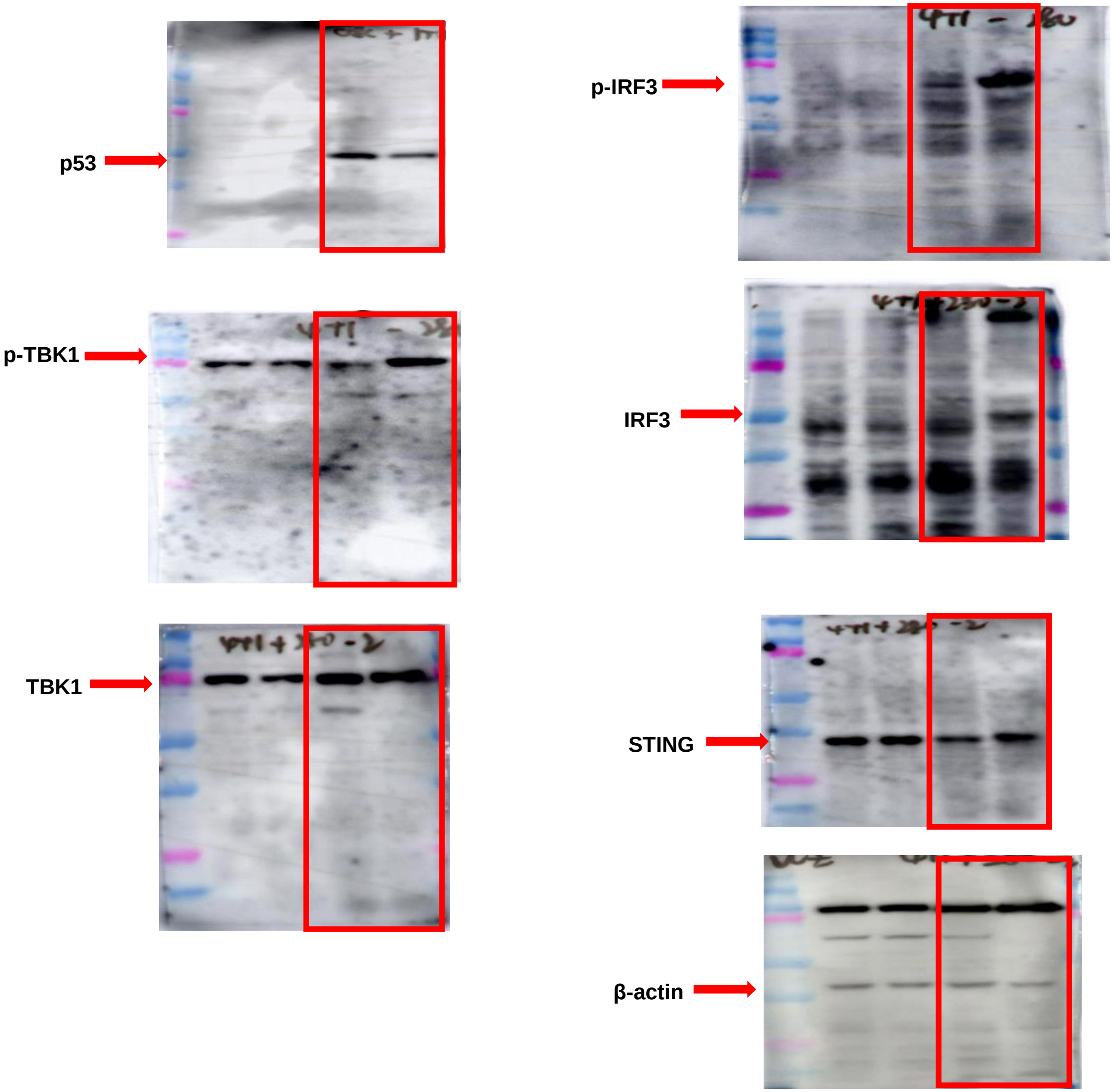

Figures3 A

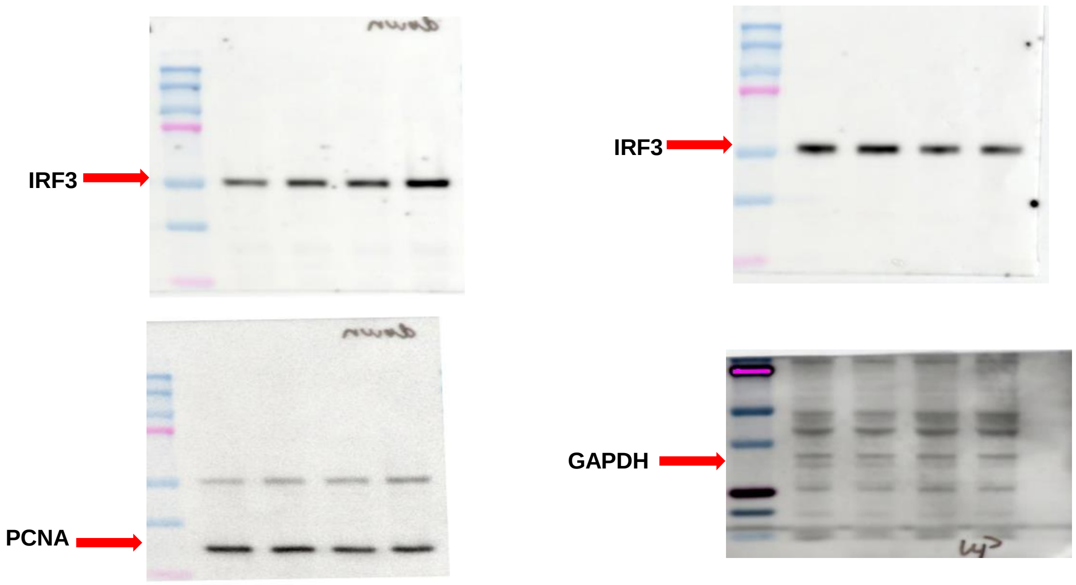

Figures3 B

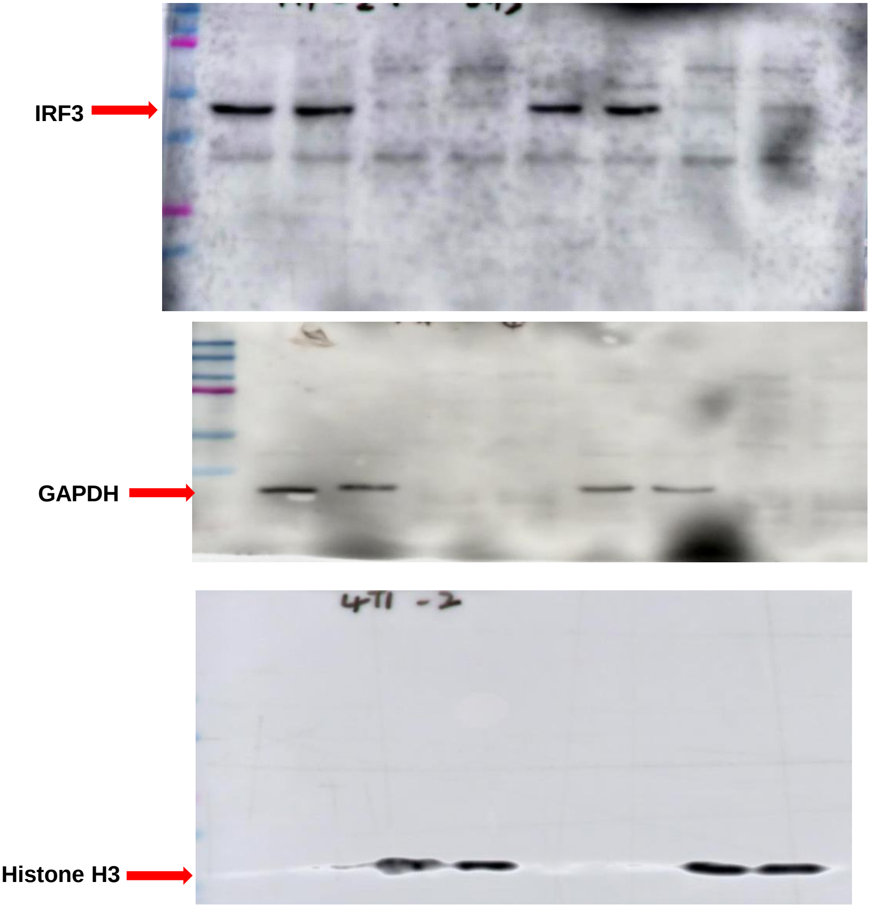

Figures4 A

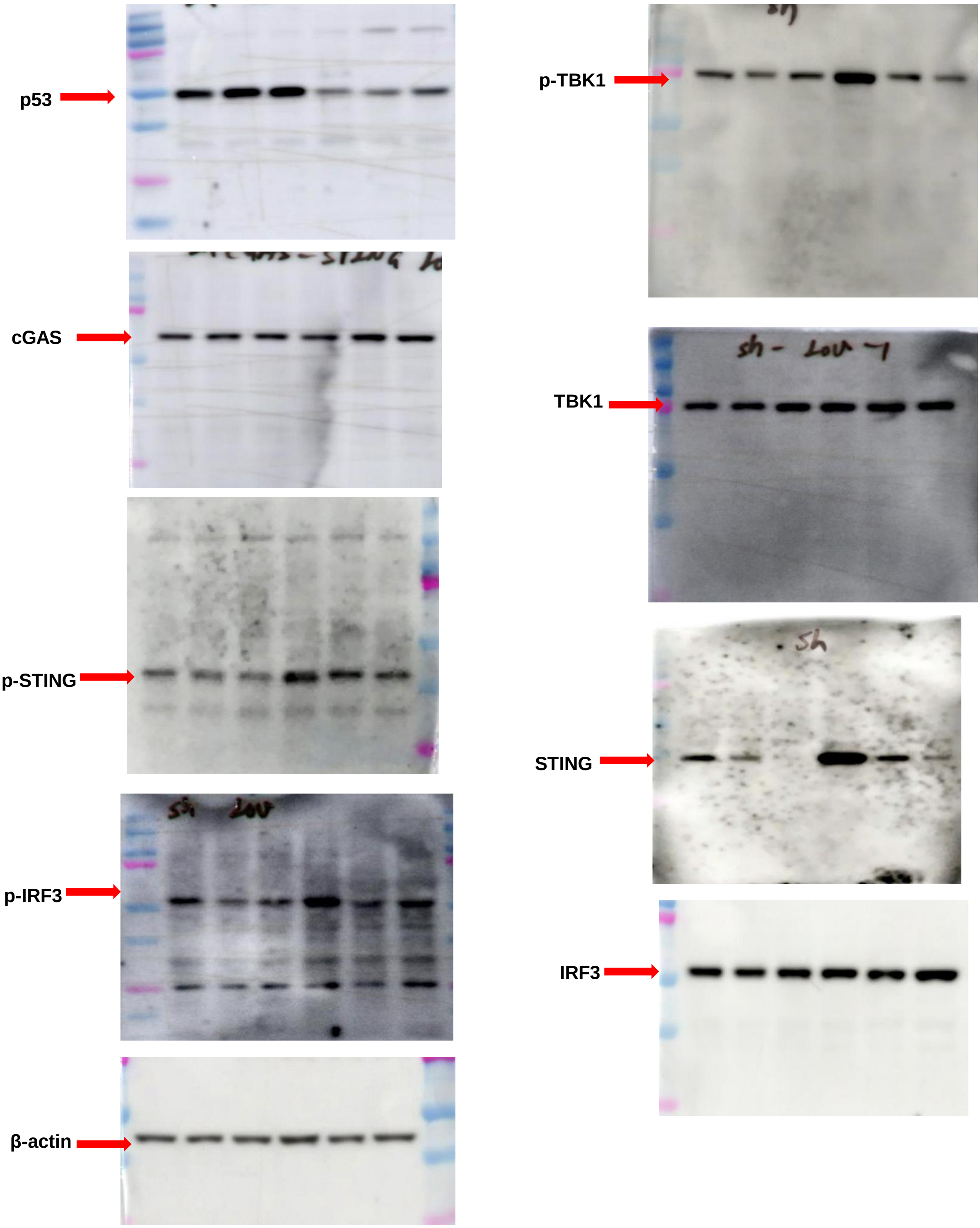

Figures6 A

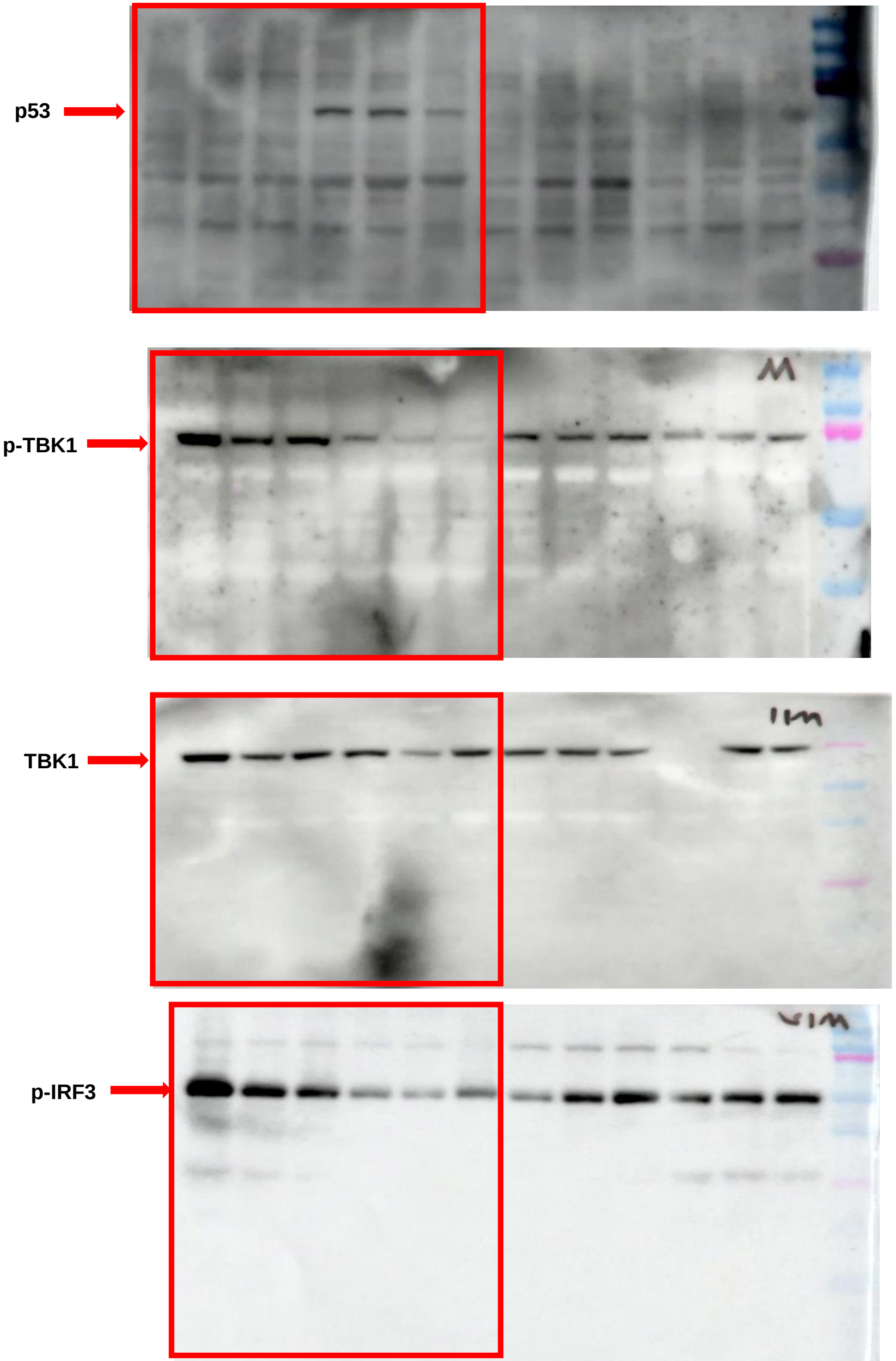

Figures6 A

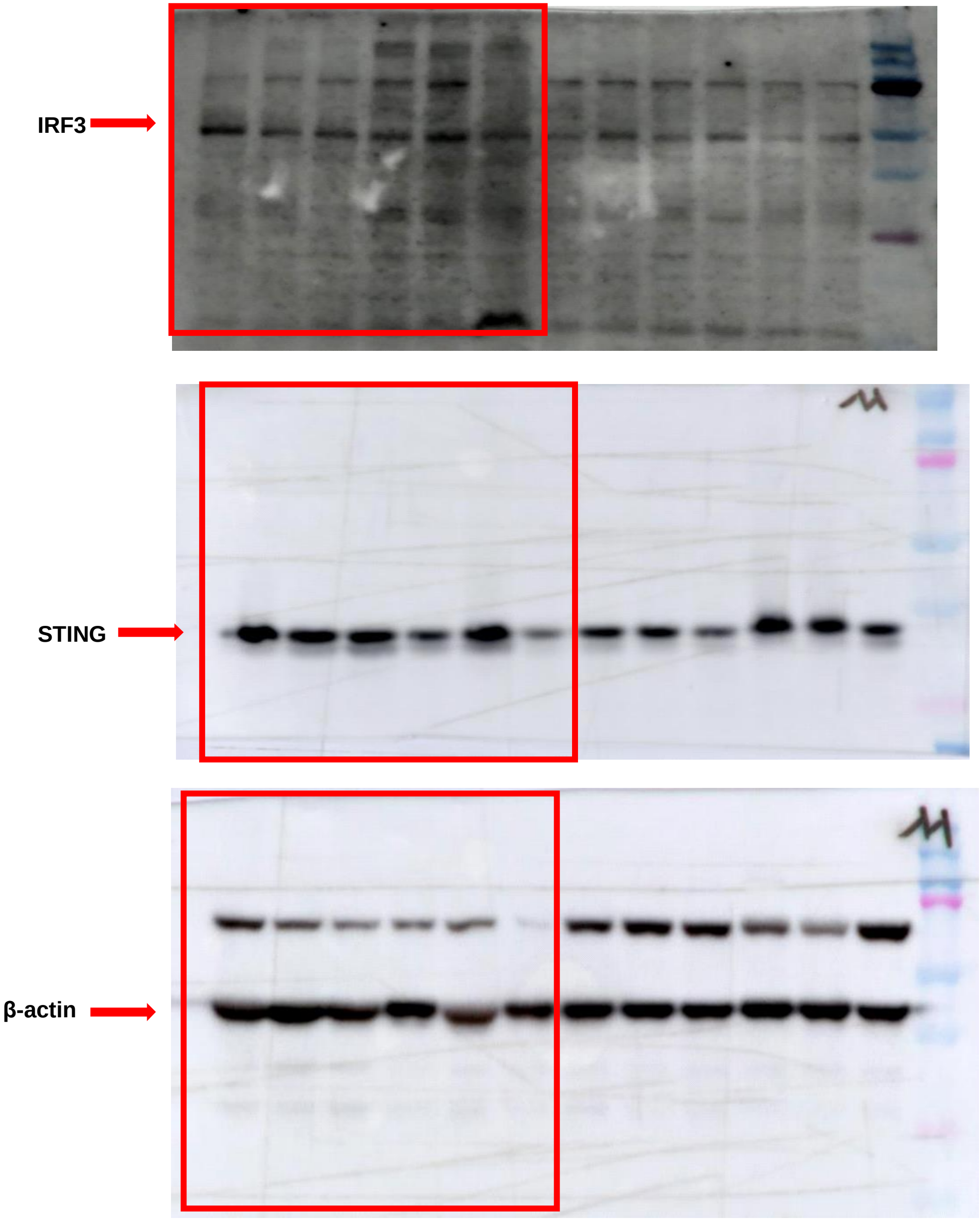

Supplementary Information

Figure S1 B

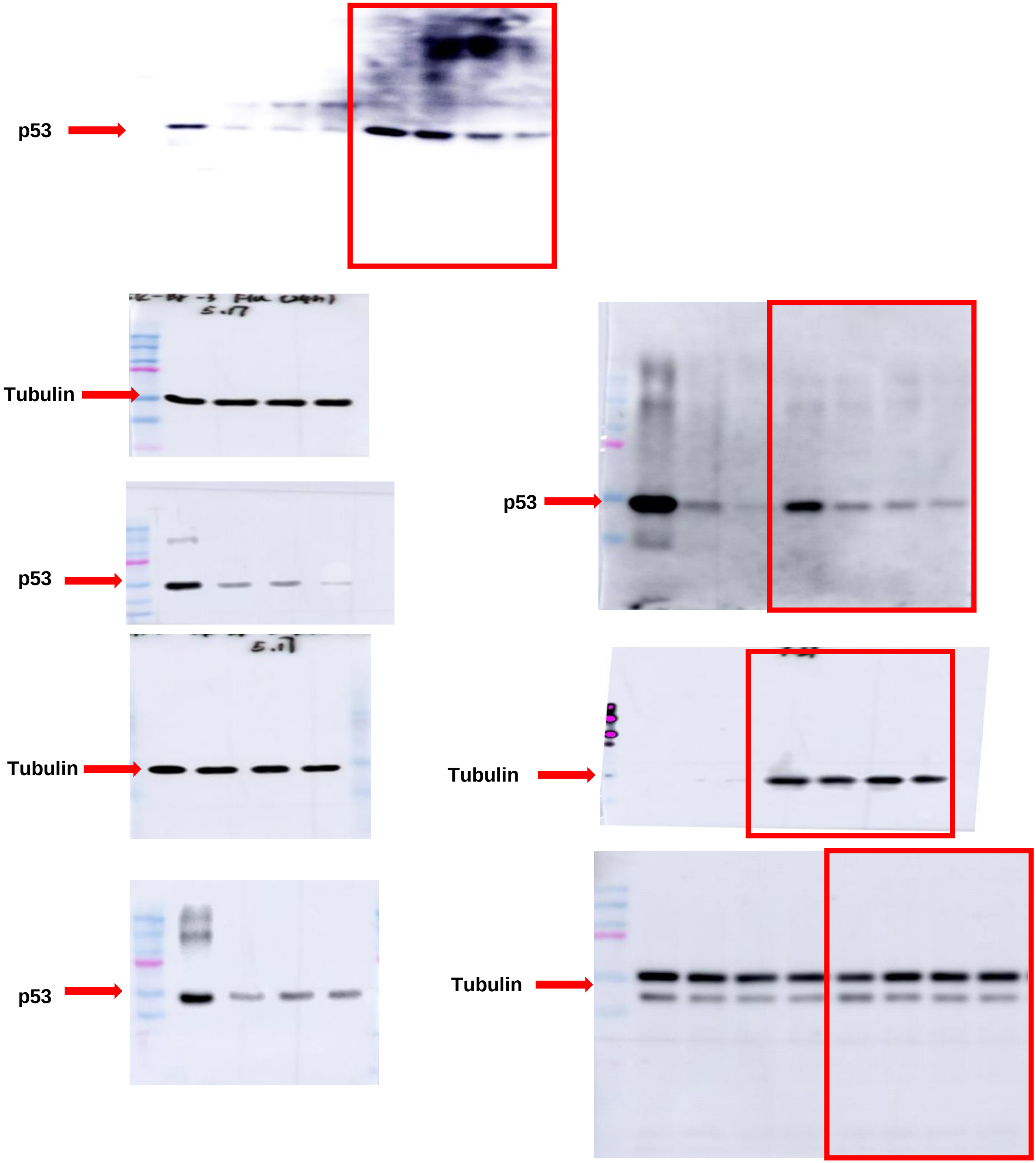

Supplementary Information
Figure S1 B

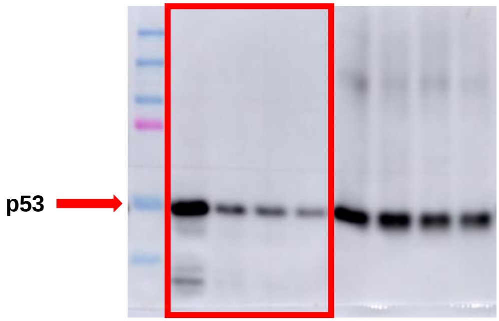

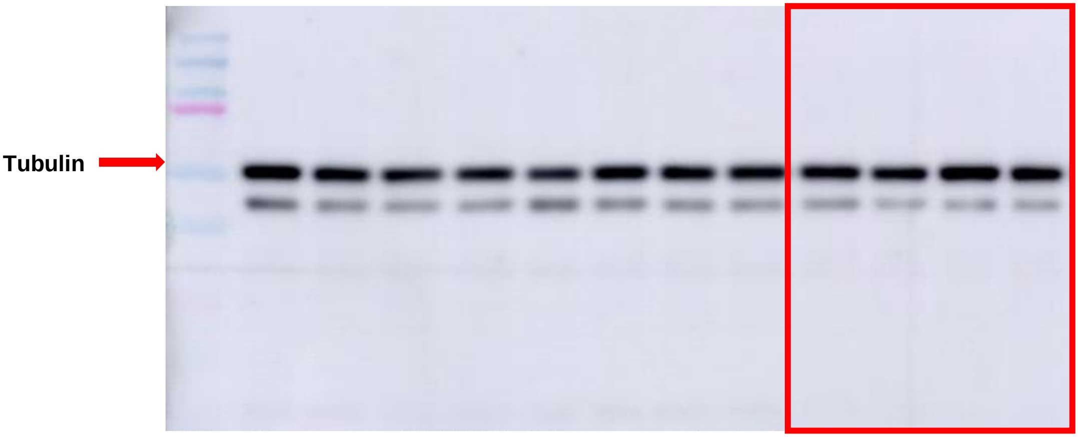

Supplementary Information
Figure S1 C

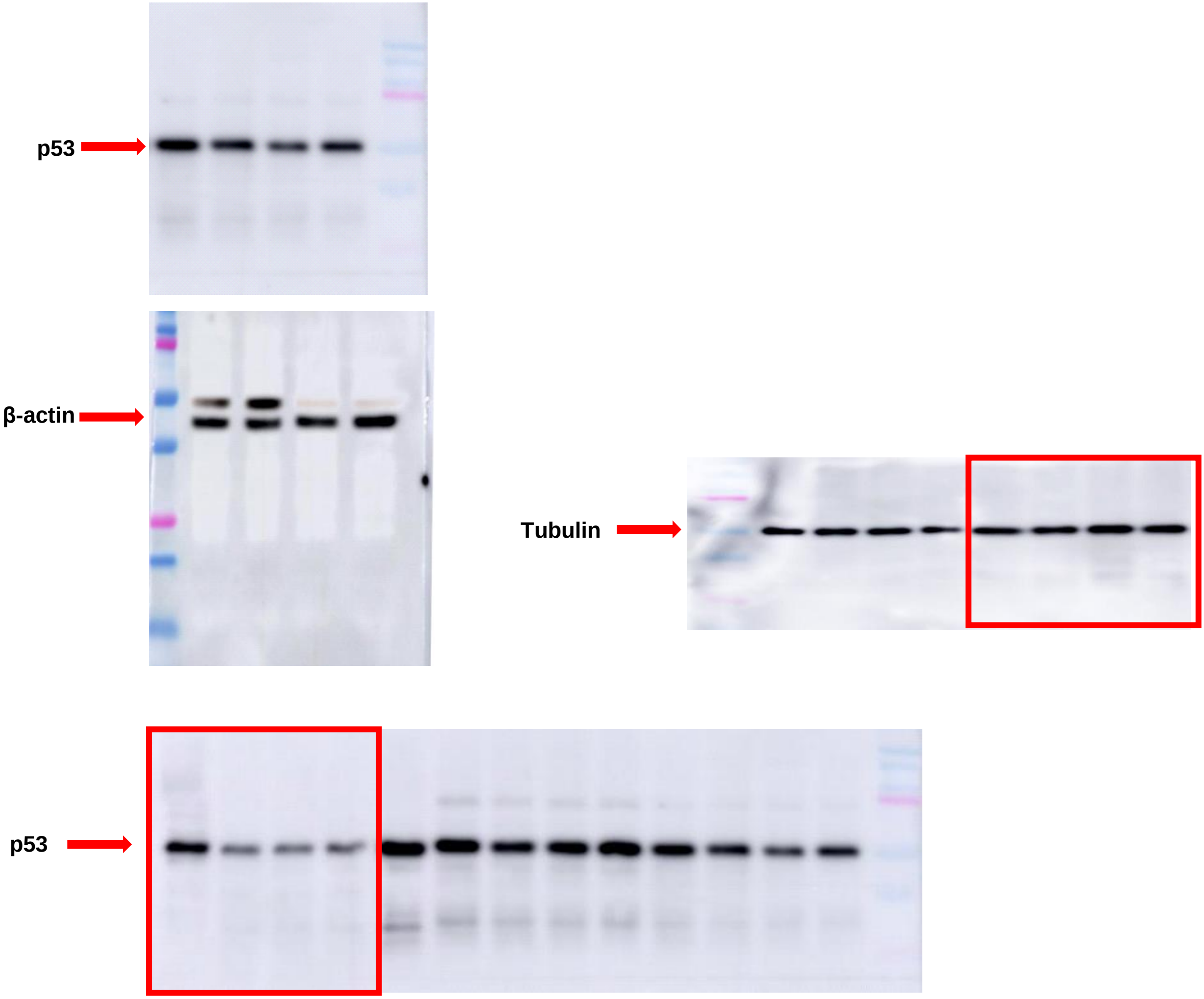

Supplementary Information
Figure S1 C

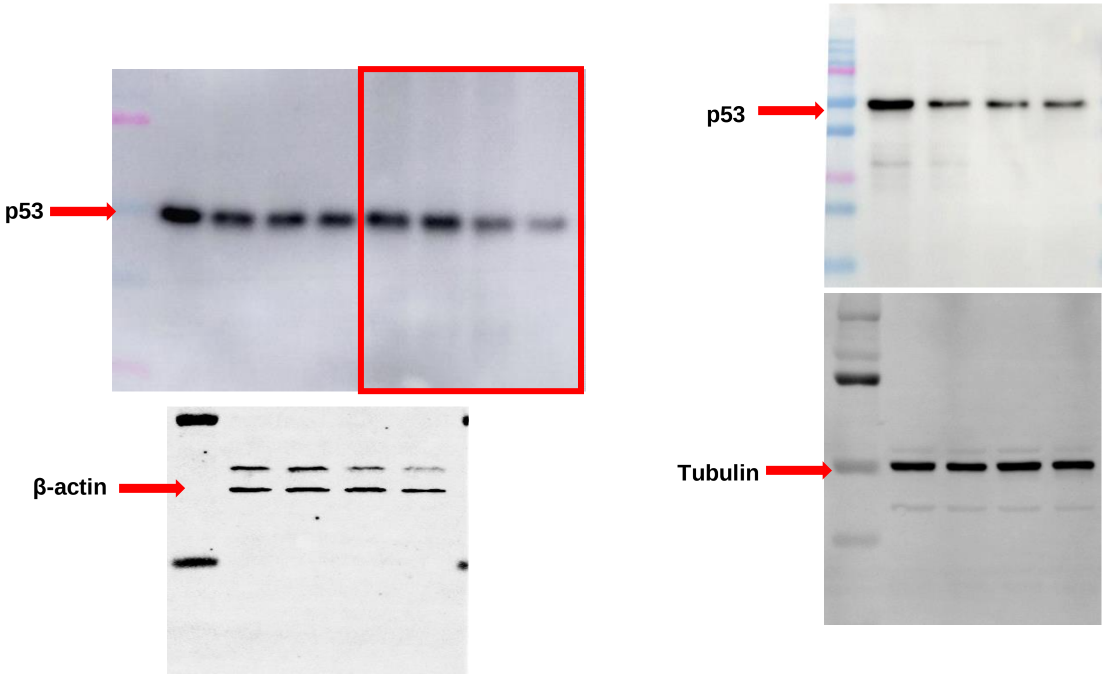

Supplementary Information

Figure S1 D

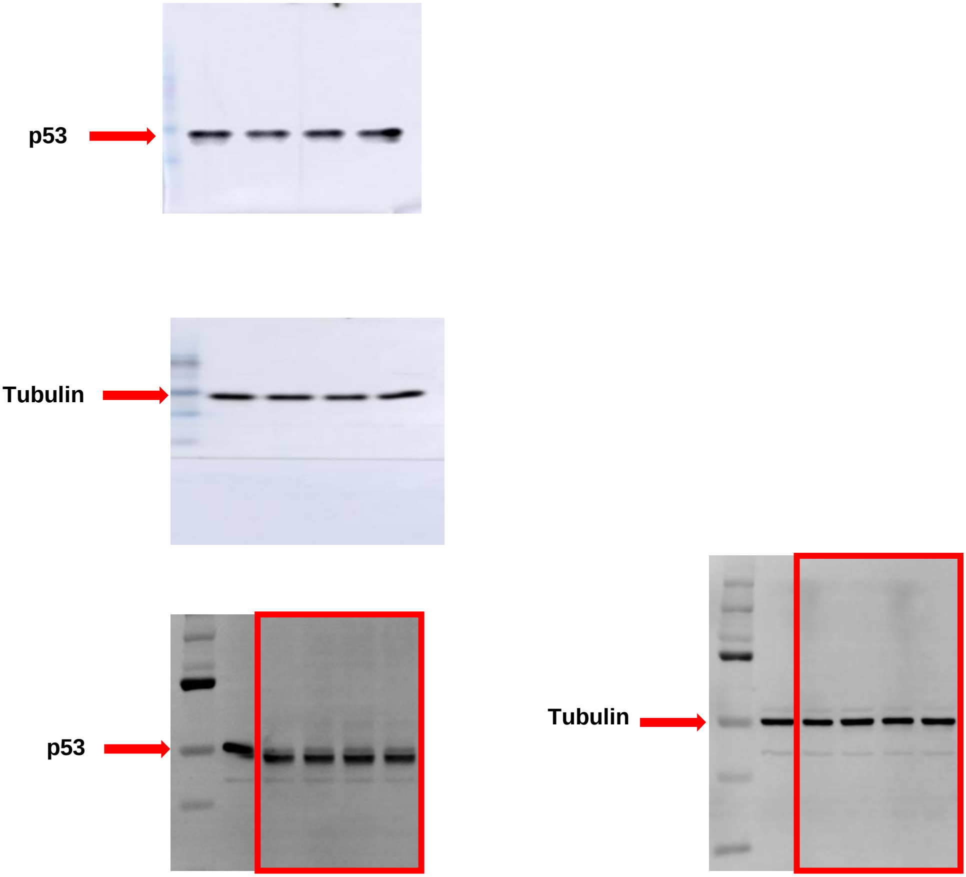

Supplementary Information

Figure S2 A

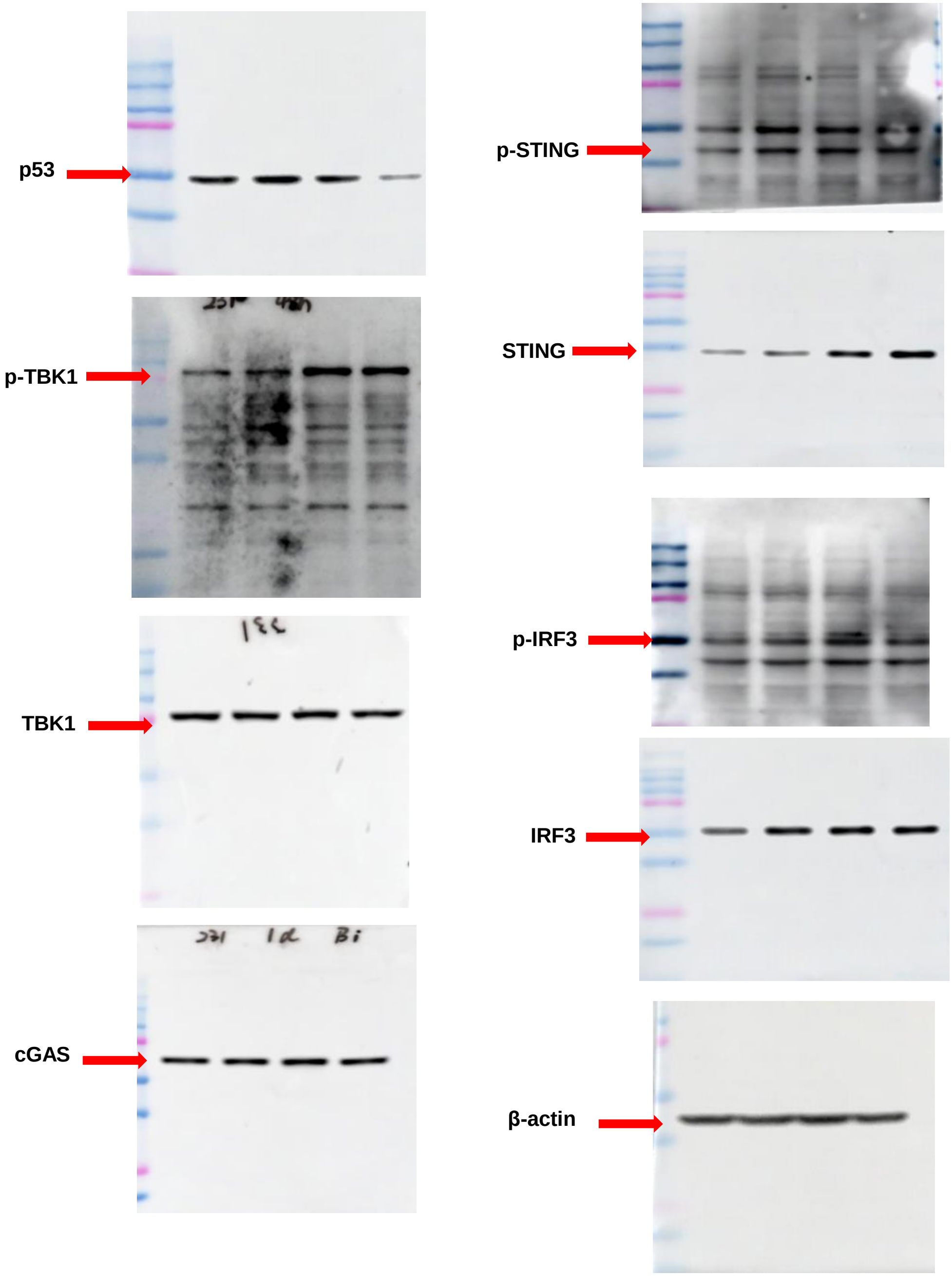

Supplementary Information

Figure S2 A

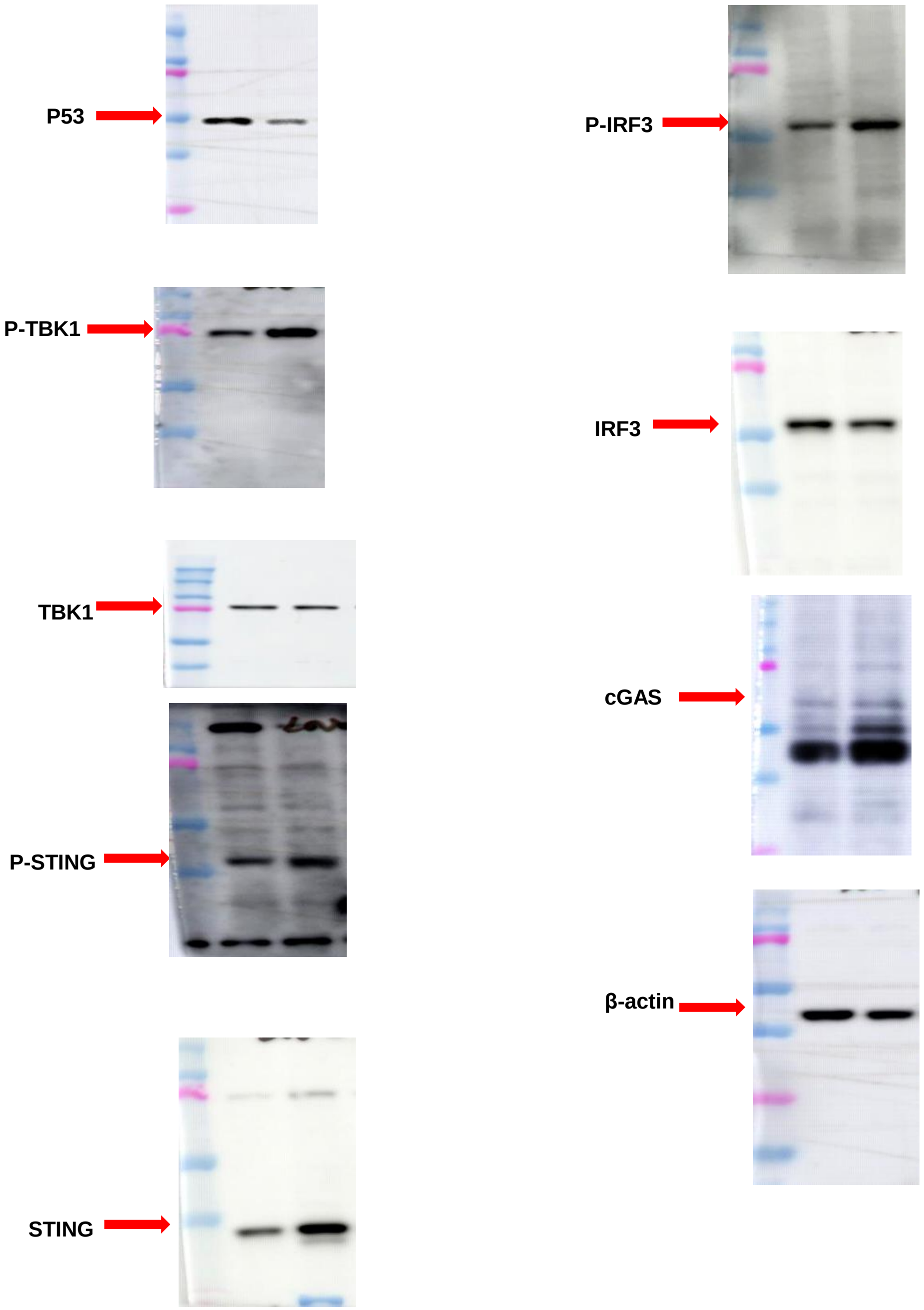

Supplementary Information
Figure S2 B

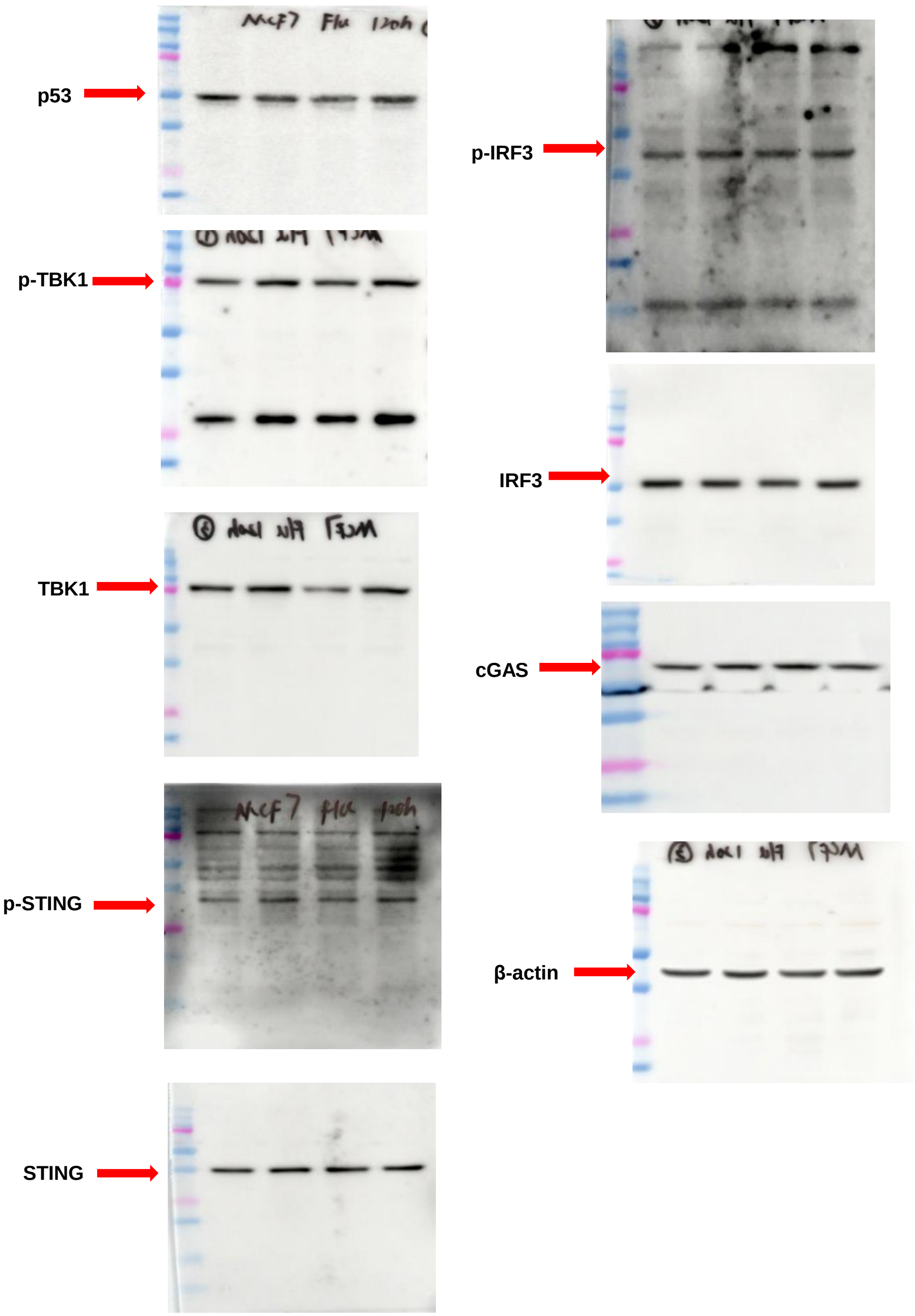

Supplement: Supplementary file 3 [file DataSheet1.pdf]
